# Pyruvate Kinase M2‐Responsive Release of Paclitaxel and Indoleamine 2,3‐Dioxygenase Inhibitor for Immuno‐Chemotherapy of Nonsmall Cell Lung Cancer

**DOI:** 10.1002/advs.202409790

**Published:** 2024-12-24

**Authors:** Haisi Wu, Xianbao Sun, Kaiming Li, Jinyu Li, Hui Jiang, Dan Yan, Ya Lin, Yan Ding, Yawen Lu, Xiaole Zhu, Xufeng Chen, Xiaolin Li, Gaolin Liang, Huae Xu

**Affiliations:** ^1^ Department of Pharmaceutics School of Pharmacy Nanjing Medical University Nanjing 211166 China; ^2^ The Affiliated Suzhou Hospital of Nanjing Medical University Suzhou Municipal Hospital Gusu School Nanjing Medical University Suzhou 215002 China; ^3^ State Key Laboratory of Digital Medical Engineering School of Biological Science and Medical Engineering Southeast University Nanjing 211189 China; ^4^ Department of Emergency The First Affiliated Hospital of Nanjing Medical University Nanjing 210029 China; ^5^ Department of Geriatric Gastroenterology The First Affiliated Hospital of Nanjing Medical University Nanjing 210029 China

**Keywords:** immuno‐chemotherapy, non‐small cell lung cancer, paclitaxel, peptide hydrogel, pyruvate kinase M2

## Abstract

Paclitaxel (PTX) is a first‐line chemotherapeutic drug for non‐small cell lung cancer (NSCLC) but it can induce indoleamine 2,3‐dioxygenase (IDO) activation, which severely lowers down its immuno‐chemotherapeutic effect. To address this issue, a smart peptide hydrogelator Nap–Phe–Phe–Phe–Lys–Ser–Thr–Gly–Gly–Lys–Ala–Pro–Arg–OH (**Nap‐T**), which co‐assembles with PTX and an IDO inhibitor GDC0919 to form a hydrogel **GP@Gel Nap‐T,** is rationally designed. Upon specific phosphorylation by pyruvate kinase M2 (PKM2), an overexpressed biomarker of NSCLC, **Nap‐T** is gradually converted to Nap–Phe–Phe–Phe–Lys–Ser–Thr(H_2_PO_3_)–Gly–Gly–Lys–Ala–Pro–Arg–OH (**Nap‐Tp**), leading to dehydrogelation and sustained release of PTX and GDC0919 within NSCLC tissues. The released PTX exerts chemotherapy on NSCLC cells as well as immunogenic cell death induction, while GDC0919 promotes the immuno‐chemotherapeutic effect of PTX through IDO inhibition. We find that **GP@Gel Nap‐T** enhances the infiltration of tumor‐infiltrating immune cells and reduces the number of immunosuppressive cells in either tumor tissues or tumor‐draining lymph nodes, thus enhancing the immuno‐chemotherapy of PTX toward NSCLC. With this PKM2‐responsive drug release strategy, the smart peptide hydrogel platform might be applied for NSCLC treatment in clinic in near future.

## Graphical abstract

A smart peptide hydrogelator **Nap‐T** is designed to co‐deliver paclitaxel and indoleamine 2,3‐dioxygenase inhibitor GDC0919, achieving sustained drug release upon pyruvate kinase M2 phosphorylation for non‐small cell lung cancer treatment. **GP@Gel Nap‐T** enhances immuno‐chemotherapeutic effect by promoting cell apoptosis, immunogenic cell death effect, and immune cell infiltration, as well as reducing immunosuppression.

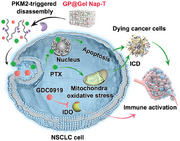

## 1 Introduction

Non‐small cell lung cancer (NSCLC) accounts for nearly 85% of lung cancer cases with fatal outcomes.^[^ ^1^ ^]^ Chemotherapy remains the primary treatment for NSCLC and paclitaxel (PTX) serves as a first‐line chemotherapeutic agent.^[^ ^2^ ^]^ PTX exerts its anticancer effects by inducing chromosome misalignment on the multipolar spindle of cancer cells, leading to increased chromosomal instability.^[^ ^3^ ^]^ Interestingly, PTX has also demonstrated the ability to provoke immunogenic cell death (ICD).^[^ ^4^ ^]^ In brief, it causes the release of various “danger signals” known as damage‐associated molecular patterns (DAMPs), such as high mobility group box 1 (HMGB1), calreticulin (CRT), adenosine triphosphate (ATP), and heat shock protein 70 kDa (HSP70).^[^ ^5^ ^]^ These DAMPs play a crucial role in recruiting antigen‐presenting cells, initiating an amplified host response to tumor cells, and activating immune cells such as dendritic cells (DCs) and T cells to fight against tumors.^[^ ^6^ ^]^

However, the immunogenic effect of PTX is severely suppressed inside tumors, particularly by indoleamine 2,3‐dioxygenase (IDO)‐mediated immunosuppressiveness,^[^ ^7^ ^]^ which lowers down the immune‐chemotherapeutic effect of PTX.^[^ ^8^ ^]^ IDO is a negative feedback enzyme overexpressed in some cancer cells. It catalyzes the degradation of tryptophan (Trp) into kynurenine (Kyn), which increases the numbers of regulatory T cells (Tregs), myeloid‐derived suppressor cells (MDSCs), and M2‐type tumor‐associated macrophages (M2‐TAMs) in tumor microenvironment (TME).^[^ ^9^ ^]^ These together suppress the activation and infiltration of effector T (CD8^+^ T) cells, natural killer (NK) cells, and M1‐TAMs, creating a favorable microenvironment for tumor growth.^[^ ^10^ ^]^

In recent years, researchers have explored to use IDO inhibitors (e.g., GDC0919/NLG919) as an adjuvant of PTX to realize the synergistic immuno‐chemotherapy of tumors.^[^ ^11^ ^]^ Of note, owing to their different physical (e.g., solubility) and pharmacokinetic properties, IDO inhibitors and PTX were encapsulated in nanoparticles in those works for efficient codelivery to tumors. However, nanoparticle‐based delivery platforms may suffer from poor bioavailability (only a median of 0.7% of the administered nanoparticles successfully reached the solid tumors^[^ ^12^ ^]^), which necessitates high injection dosages for therapeutic effectiveness thus leads to non‐negligible adverse effects,^[^ ^13^ ^]^ such as allergic reactions and bone marrow suppression.^[^ ^14^ ^]^ Therefore, efficient and safe codelivery of PTX and GDC0919 to tumor tissues still remains as a crucial yet unresolved challenge.

Currently, injected hydrogels have been considered as a highly promising platform for localized delivery and controlled release of drugs. They provide significantly increased local drug concentrations, rendering improved bioavailability and safety, as well as other advantages that benefit cancer treatments.^[^ ^15^ ^]^ Among various hydrogels, supramolecular peptide hydrogels, which were assembled by peptide gelators through noncovalent interactions (e.g., hydrogen bonds, π–π stacking, hydrophobic or electrostatic interactions), have shown advantages in drug delivery.^[^ ^16^ ^]^ They possess inherent biocompatibility and biodegradability, facile synthesis of peptide gelators, exceptional injectability, and efficient drug loading capabilities.^[^ ^17^ ^]^ More importantly, on‐demand tailoring of the peptide gelators is allowed, which endows the hydrogels with “smart” responsiveness toward pathological cues of interest (e.g., enzymes, pH).^[^ ^18^ ^]^ Through direct injection on the pathological focus, these “smart” peptide hydrogels undergo disassembly of the gelators and therefore dehydrogelation in response to the cues, releasing the drug cargoes in a precise and sustainable manner. However, to the best of our knowledge, such advantageous peptide hydrogels that enable efficient and smart codelivery of PTX and GDC0919 for immuno‐chemotherapy of cancers have not been reported.

**Scheme 1:**
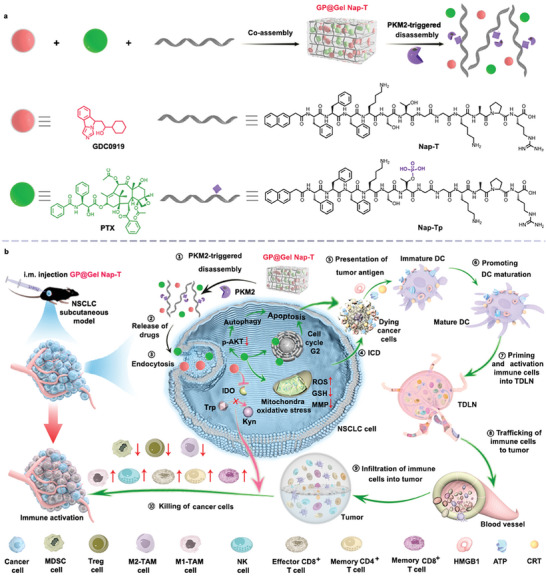
Schematic illustration of the components, PKM2‐triggered transformation and mechanism of **GP@Gel Nap‐T**. a) Schematic illustration of the formation and PKM2‐triggered transformation of **GP@Gel Nap‐T**, and the chemical structures of its components. b) Mechanism of **GP@Gel Nap‐T** for chemo‐immune therapy of NSCLC.

Herein, we designed a smart peptide hydrogelator Nap–Phe–Phe–Phe–Lys–Ser–Thr–Gly–Gly–Lys–Ala–Pro–Arg–OH (**Nap‐T**) that was responsive to pyruvate kinase M2 (PKM2), aiming to improve chemo‐immunotherapy of NSCLC through localized codelivery and controlled release of PTX and GDC0919. **Nap‐T** contained two segments: a self‐assembling peptide sequence Nap–Phe–Phe–Phe (Nap‐FFF), and a peptide sequence Lys–Ser–Thr–Gly–Gly–Lys–Ala–Pro–Arg–OH (KSTGGKAPR) serving as a phosphorylation substrate of PKM2,^[^ ^19^ ^]^ who is a key glycolytic kinase overexpressed in the NSCLC cell microenvironment (**Scheme** **1**a).^[^ ^20^ ^]^ **Nap‐T** self‐assembled under physiological conditions, during which PTX and GDC0919 were coassembled to afford the hydrogel platform **GP@Gel Nap‐T**. PKM2 could phosphorylate the threonine residue in the gelator **Nap‐T**, which converted the hydrophobic **Nap‐T** to a hydrophilic phosphorylated analogue Nap–Phe–Phe–Phe–Lys–Ser–Thr(H_2_PO_3_)–Gly–Gly–Lys–Ala–Pro–Arg–OH (**Nap‐Tp**), leading to a gel‐to‐solution conversion of **GP@Gel Nap‐T** accompanied by the release of PTX and GDC0919 (i.e., PKM2‐triggered disassembly). The chemo‐immunotherapy mechanism of **GP@Gel Nap‐T** involves multiple steps. Upon injection of **GP@Gel Nap‐T** into the NSCLC tumor model, the overexpressed PKM2 in tumor triggers the disassembly of **GP@Gel Nap‐T** (i.e., ① PKM2‐triggered disassembly) to release PTX and GDC0919 (i.e., ② Release of drugs). After PTX and GDC0919 are internalized by cancer cells (i.e., ③ Endocytosis), on the one hand, the released PTX triggers multifaceted responses. It prompts cancer cell apoptosis by downregulating p‐AKT in cell to activate autophagy and arresting cell proliferation at the G2 stage. Meanwhile, PTX induces mitochondrial oxidative stress (e.g., upregulation of reactive oxygen species (ROS), downregulation of glutathione (GSH) and mitochondrial membrane potential (MMP)) to provoke ICD (i.e., ④ ICD). These factors collectively contribute to cell death and the following immune response, including: ⑤ Presentation of tumor antigen, ⑥ Promoting DCs maturation, ⑦ Priming and activation immune cells into tumor‐draining lymph nodes (TDLNs), ⑧ Trafficking of immune cells into tumors, and ⑨ Infiltration of immune cells into tumors. On the other hand, the released GDC0919 inhibits the activity of IDO, leading to a decrease of M2‐TAMs, Tregs, or MDSCs, but an increase of M1‐TAMs, NK cells, CD8^+^ T cells, memory CD4^+^ T cells, and memory CD8^+^ T cells. The combination of these two pathways renders efficient NSCLC cell death (i.e., ⑩ Killing of cancer cells) and provides long‐term immune memory effects for enduring immune protection (Scheme 1b).

## 2 Results and Discussion

### 2.1 Synthesis and Characterization of GP@Gel Nap‐T

We first synthesized the compounds **Nap‐T** (Nap–Phe–Phe–Phe–Lys–Ser–Thr–Gly–Gly–Lys–Ala–Pro–Arg–OH) and its phosphorylated derivative **Nap‐Tp** (Nap–Phe–Phe–Phe–Lys–Ser–Thr(H_2_PO_3_)–Gly–Gly–Lys–Ala–Pro–Arg–OH) through solid‐phase peptide synthesis (SPPS) (SchemesS1 and S2, Supporting Information). These compounds were purified with high performance liquid chromatography (HPLC), and characterized with matrix‐assisted laser desorption ionization time‐of‐flight mass spectrometry (MALDI‐TOF‐MS) and nuclear magnetic resonance (NMR) spectrometry (Figures S1–S6, Supporting Information). The critical aggregation concentrations (CACs) of **Nap‐T** and **Nap‐Tp** were measured as 312.53 and 813.26 µm, respectively (Figure S7, Supporting Information). Subsequently, we validated whether **Nap‐T** could assemble into a hydrogel. 1.0 wt% **Nap‐T** in phosphate‐buffered saline (PBS, 0.01 m, pH 7.4) underwent a heating–cooling process (heating at 65 °C to dissolve the compound, then cooling down at room temperature), then a stable hydrogel (i.e., **Gel Nap‐T**) was observed (Figure S8a, Supporting Information), suggesting that **Nap‐T** could assemble into a hydrogel at the above condition. In contrast, **Nap‐Tp** at the above condition remained in its liquid state (Figure S8b, Supporting Information).

**Figure 1:**
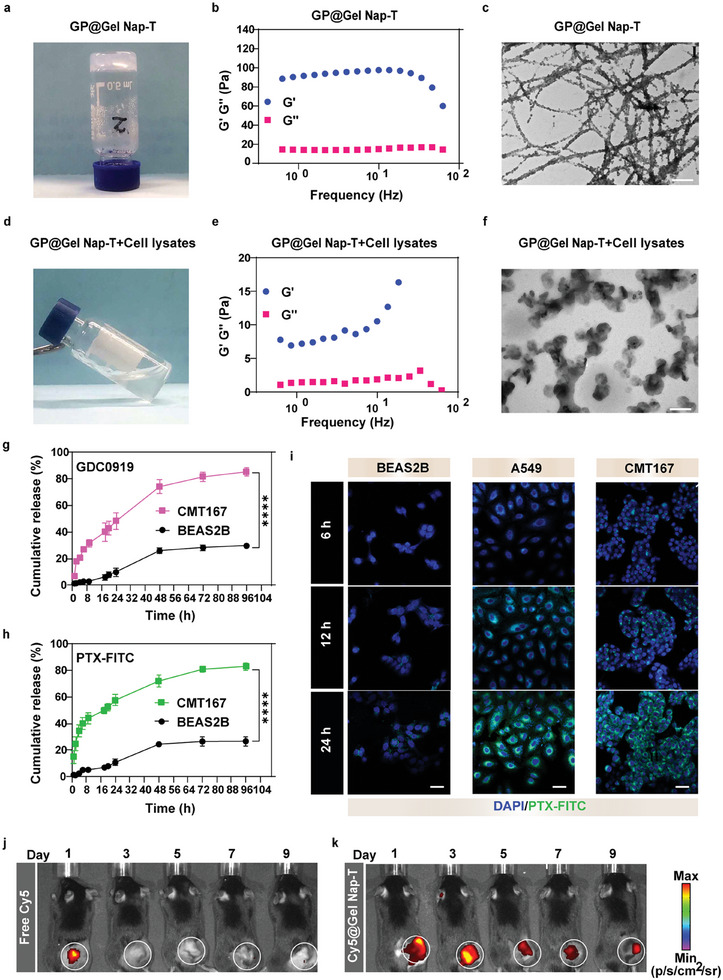
Characterizations of **GP@Gel Nap‐T** and PKM2‐triggered disassembly of **GP@Gel Nap‐T**. a) Optical images of 1.0 wt% **GP@Gel Nap‐T** after heating–cooling process. b) Frequency dependence of the dynamic storage moduli (*G*′) and the loss moduli (*G*″) of 1.0 wt% **GP@Gel Nap‐T** (strain: 1.0%). c) TEM images of 1.0 wt% **GP@Gel Nap‐T**. Scale bar, 0.5 µm. d) Optical images of 1.0 wt% **GP@Gel Nap‐T** after incubation with cell lysates overnight at 37 °C, respectively. e) Frequency dependence of the dynamic storage moduli (*G*′) and the loss moduli (*G*″) of 1.0 wt% **GP@Gel Nap‐T** after treatment as in (d) (strain: 1.0%). f) TEM image of **GP@ Gel Nap‐T** after treatment as in (d). Scale bar: 0.5 µm. g,h) Cumulative release profiles of GDC0919 and PTX‐FITC in culture medium after incubation of **GP@Gel Nap‐T** with BEAS2B and CMT167 cells at different times (1 h, 2 h, 4 h, 6 h, 9 h, 18 h, 20 h, 24 h, 48 h, 72 h, and 96 h). PTX was labeled by FITC (*n* = 3 biological independent samples). i) Representative CLSM images of (left) BEAS2B, (middle) A549, and (right) CMT167 cells after incubation with **P@Gel Nap‐T** for 6 h, 12 h, and 24 h. DAPI (blue), PTX‐FITC‐label (green). Scale bars: 50 µm (*n* = 3 biological independent samples). j,k) Real‐time fluorescence biodistribution images of CMT167 tumor‐bearing mice taken on day 1, 3, 5, 7, and 9 after intratumoral injection. j) free Cy5 and k) **Cy5@Gel Nap‐T** (*n* = 3 biological independent samples). Results are presented as mean ± SD. Statistical significance was assessed using one‐way ANOVA with Tukey's post‐test. **** *P* < 0.0001.

PTX and GDC0919 were then co‐assembled with **Nap‐T**. We first evaluated the loading capacity of **Nap‐T** toward the drugs. 1.0 wt% **GP@Gel Nap‐T** mixtures with different GDC0919/PTX/**Nap‐T** molar ratios (1:1:6.25, 1:1:12.5, 1:1:25, 1:1:50, 1:1:100, 1:1:200, and 1:1:400) were prepared and underwent the heating–cooling process. We found that stable gels were formed for all these groups, demonstrating the facile encapsulation of drugs GDC0919 and PTX in the hydrogel **Gel Nap‐T** (Figure S9, Table S1, Supporting Information). After that, 1.0 wt% **Nap‐T** in 0.2 mL PBS (0.01 m, pH 7.4) was added with 1 µg mL^−1^ GDC0919 and PTX, then subjected to the heating–cooling process described above. As expected, a homogeneous hydrogel was formed (i.e., **GP@Gel Nap‐T**) (**Figure** **1**a). Then, the rheological properties of the drug‐loaded hydrogel **GP@Gel Nap‐T** were evaluated. Similar with those of **Gel Nap‐T** (Figures S10 and S11a, Supporting Information), the storage modulus (*G*′) values of **GP@Gel Nap‐T** were higher than the corresponding loss modulus (*G*″) values across the investigated frequency (0.1–100 Hz) (Figure 1b) or strain ranges (0.0001–1%) (Figure S11b, Supporting Information), demonstrating that **GP@Gel Nap‐T** was an elastic solid (i.e., gel). Circular dichroism (CD) was employed to investigate the secondary structures of the assemblies in the hydrogels. The CD spectra showcased positive peaks at 190 nm and negative peaks at 210 nm for **Gel Nap‐T** and **GP@Gel Nap‐T** (Figure S12, Supporting Information), indicating the formation of β‐sheet‐like secondary structures within both hydrogels.^[^ ^21^ ^]^ The observed positive peak at 230 nm in the CD spectra likely corresponds to the chiral phenylalanine residue.^[^ ^22^ ^]^ The transmission electron microscopy (TEM) images indicated that the micromorphology of **GP@Gel Nap‐T** was entangled and densely packed nanofibers with an average diameter of 62.25 ± 5.19 nm (Figure 1c; Figure S13, Supporting Information), which was thicker than that of **Gel Nap‐T** (i.e., 40.69 ± 3.99 nm) owing to the drug‐participated coassembly (Figure S14, Supporting Information).^[^ ^15b^ ^]^ Taken together, the above results suggested that the drugs (i.e., PTX and GDC0919) and **Nap‐T** could co‐assemble to form a hydrogel **GP@Gel Nap‐T**.

### 2.2 Responsiveness of GP@Gel Nap‐T to PKM2

The Cancer Genome Atlas (TCGA) database revealed that PKM2 was upregulated in various human tumors (Figure S15, Supporting Information), and we observed substantial PKM2 overexpression in two NSCLC cell lines (i.e., a murine cell line CMT167, a human cell line A549) and some other cancer cell lines, coupled with its notable secretion into the conditioned media (Figures S16 and S17, Supporting Information). After confirming this, we validated whether PKM2 could mediate the disassembly of **GP@Gel Nap‐T**. Initially, the as‐prepared hydrogel **GP@Gel Nap‐T** was exposed to the lysates of CMT167 cells and incubated at 37 °C overnight. As anticipated, the gel **GP@Gel Nap‐T** underwent a gel‐to‐solution transition upon exposure to CMT167 cell lysates (Figure 1d), while the gel **GP@Gel Nap‐T** treated with PBS remained in the gel state (Figure S18, Supporting Information). Moreover, the dynamic rheological tests showed that the *G*′ values of cell lysate‐treated **GP@Gel Nap‐T** increased with the frequency (Figure 1e) and decreased with the strain (Figure S19, Supporting Information), demonstrating the fluid state of **GP@Gel Nap‐T** after treatment with PKM2‐containing cell lysates. Notably, these trends were analogous to those observed in **Gel Nap‐T** (Figure S20, Supporting Information), where the *G* values of cell lysate‐treated **Gel Nap‐T** also increased with frequency (Figure S20a, Supporting Information) and declined with strain (Figure S20b, Supporting Information). The TEM image revealed that, upon the above treatment, the micromorphology of **GP@Gel Nap‐T** and **Gel Nap‐T** transformed from nanofibers to irregular and dispersed nanoparticles (Figure 1f; Figure S21, Supporting Information). Then, HPLC analysis was performed to reveal **GP@Gel Nap‐T** underlying chemical evolution. The retention time of **Nap‐T** was precisely aligning with the retention time of **Nap‐Tp** after exposure to CMT167 cell lysates at 37 °C overnight (Figure S22, Supporting Information), thus suggesting the efficient phosphorylation of **Nap‐T** into **Nap‐Tp**. Furthermore, we observed that in the presence of a PKM2 inhibitor PKM2‐IN‐1, **Nap‐T** failed to be converted to **Nap‐Tp** after exposure to cell lysate, indicating that the conversion process was indeed PKM2‐dependent (Figure S22, Supporting Information). Additionally, we further evaluated the stability of **Gel Nap‐T** and **GP@Gel Nap‐T** in PKM2‐free cell culture medium. **Gel Nap‐T** and **GP@Gel Nap‐T** remained their morphological integrity in Dulbecco's modified Eagle medium (DMEM) medium for 9 days, demonstrating their robustness in PKM2‐free conditions (Figure S23, Supporting Information). These results collectively demonstrated that PKM2 could trigger the disassembly of the hydrogel **GP@Gel‐Nap‐T**.

### 2.3 Drug Release Profiles of GP@Gel Nap‐T

The drug release behaviors of the hydrogel **GP@Gel Nap‐T** upon PKM2‐mediated disassembly were then testified. To facilitate its monitoring in cell experiments, PTX was labeled with a fluorochrome FITC (i.e., PTX‐FITC). PTX‐FITC was encapsulated along with GDC0919 in the hydrogel to afford **GP@Gel Nap‐T**. To investigate the drug release kinetics, 100 µL of the as‐prepared **GP@Gel Nap‐T** was incubated with 5.0 × 10^5^ CMT167 cells in 1.0 mL of cell culture medium. Over different incubation durations, the culture mediums were collected for HPLC analysis. We observed sustained release profiles for both GDC0919 (Figure 1g) and PTX‐FITC (Figure 1h) in the CMT167 cell medium. At 96 h, ≈85.3% of GDC0919 and 83.2% of PTX‐FITC were released from the hydrogel. In contrast, in the control group the gel **GP@Gel Nap‐T** incubated with BEAS2B cells, which had a relatively low PKM2 expression (Figure S16, Supporting Information), showed low release profiles and much lower release ratios (less than 30.0%) for both drugs at 96 h. In addition, further analysis of the CMT167 cell medium at 20 h indicated the presence of the phosphorylated product (i.e., **Nap‐Tp**) (Figure S24, Supporting Information), suggesting the phosphorylation of **Nap‐T** by PKM2 in the CMT167 cell medium. Collectively, these results suggested the sustained release of drugs GDC0919 and PTX‐FITC from **GP@Gel Nap‐T** by CMT167 cells through the PKM2‐triggered disassembly mechanism as depicted above.

### 2.4 Release and Uptake of Drugs from GP@Gel Nap‐T in Cell and Mouse Models

The hydrogel loaded with PTX‐FITC was incubated with model cells to validate the cellular uptake profiles of the released drug via confocal laser scanning microscopy (CLSM). A PKM2‐lowexpressing NSCLC cell line (i.e., BEAS2B) and two PKM2‐overexpressing NSCLC cell lines (i.e., A549 and CMT167) were employed as the model cell lines. The hydrogel‐treated BEAS2B cells always exhibited weak FITC green fluorescence (intensity: 14.2 au) over 24 h, demonstrating the poor uptake of PTX‐FITC into BEAS2B cells (Figure 1i; Figure S25, Supporting Information), which was ascribed to the low expression of PKM2 to disintegrate the hydrogel. In contrast, for both A549 and CMT167 cells, the green fluorescence signals increased with time, reaching significantly bright intensities of 62.2 and 89 au, respectively, at 24 h (Figure 1i; Figure S25, Supporting Information). These results suggested efficient enzymatic release and cellular uptake of the drug from the hydrogel into PKM2‐overexpressing NSCLC cells. Moreover, we further validated the sustained release property of the hydrogel in vivo. To validate the drug release profiles from the hydrogel in animal experiments, a near‐infrared dye Cy5 was encapsulated in the hydrogel **Gel Nap‐T** to afford **Cy5@Gel Nap‐T**. After in situ injection of free Cy5 and the as‐prepared hydrogel **Cy5@Gel Nap‐T** on CMT167 tumors of the mice, persistent and robust Cy5 fluorescence signals were observed even after 9 days postinjection in **Cy5@Gel Nap‐T** group, while no fluorescence signal was detected in the free Cy5 group (Figure 1j,k; Figure S26, Supporting Information), indicating the sustained drug release ability of our hydrogel in vivo, which benefited the preserving of drugs in TME for efficacious treatment outcomes.

### 2.5 Enhanced Anti‐metastasis and Anti‐proliferation of GP@Gel Nap‐T via Potentiated Autophagy and Cell Cycle Arrest In Vitro

**Figure 2:**
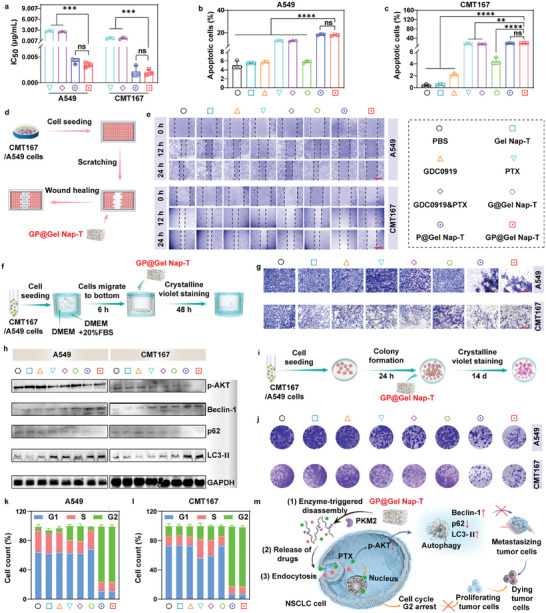
Effects of **GP@Gel Nap‐T** on the cell viability, apoptosis/necrosis, migration, autophagy‐associated proteins expression, proliferation, and cell cycle in vitro. a) The IC_50_ of PTX, GDC0919&PTX, **P@Gel Nap‐T,** and **GP@Gel Nap‐T** against A549 and CMT167 cells for 48 h (*n* = 3, biological independent samples). b,c) FCM quantification results of (b) A549 and (c) CMT167 cells after treatment with each formulation for 48 h stained with PI and Annexin V (*n* = 3, biological independent samples). d) Schematic illustration of wound healing to evaluate cell migration ability. e) Wound healing results of (up) A549 and (down) CMT167 cells after treatment with each formulation for 0 h, 12 h, 24 h. Scale bars: 50 µm (*n* = 3, biological independent samples). f) Schematic illustration of cell transwell to evaluate cell migration ability. g) Representative images of (up) A549 and (down) CMT167 cells after treatment with each formulation for 48 h in transwell migration assays. Scale bars: 50 µm (*n* = 3, biological independent samples). h) WB results of autophagy‐associated proteins and its upstream proteins expression on (left) A549 and (right) CMT167 cells after treatment with each formulation. Control, GAPDH (*n* = 3, biological independent samples). i) Schematic illustration of cell clonogenicity assay to evaluate cell proliferation ability. j) Representative images of (up) A549 and (down) CMT167 cells after treatment with each formulation for 14 d in cell clonogenicity assay (*n* = 3, biological independent samples). k,l) Quantitative FCM analysis of cell cycle on (k) A549 and (l) CMT167 cells after treatment with each formulation for 48 h (*n* = 3, biological independent samples). m) Schematic illustration the antitumor mechanism of **GP@Gel Nap‐T** in vitro. Results are presented as mean ± SD. Statistical significance was assessed using one‐way ANOVA with Tukey's post‐test. ns: no significant difference, **P < 0.01, ****P < 0.0001.

We then validated the anticancer effects of **GP@Gel Nap‐T**. First, we verified the biocompatibility of our peptide hydrogel platform using 3‐(4,5‐dimethylthiazol‐2‐yl)‐2,5‐diphenyltetrazolium bromide (MTT) assays. The results demonstrated that **Gel Nap‐T** at concentrations up to 1600 µm was nearly nontoxic to NSCLC cells (i.e., A549 and CMT167 cells) and human normal pulmonary epithelial cells (i.e., BEAS2B cell), indicating excellent biocompatibility of the hydrogel (Figure S27, Supporting Information). After that, we set eight groups for comparison: “PBS”, “**Gel Nap‐T**”, “GDC0919”, “**G@Gel Nap‐T**” (i.e., GDC0919‐loaded **Gel Nap‐T**), “PTX”, “GDC0919&PTX”, “**P@Gel Nap‐T**” (i.e., PTX‐loaded **Gel Nap‐T**), and “**GP@Gel Nap‐T**”. The first and last four groups were PTX‐absent and PTX‐present groups, respectively. To assess the potential influence of the hydrogel on PTX and GDC0919 mediated cytotoxicity effects, we conducted further experiments. Free PTX and GDC0919&PTX exhibited concentration‐dependent cytotoxicity (Figures S28 and S29, Supporting Information). Treatment with 50 ng mL^−1^ **GP@Gel Nap‐T** for 72 h resulted in a significant decrease in the viability of A549 cells and CMT167 cells to 4.5% and 2.73%, respectively. In contrast, GDC0919 and **G@Gel Nap‐T** showed minimal toxicity toward A549 cells and CMT167 cells (Figure S30, Supporting Information). Comparing the half inhibitory concentration (IC_50_) values, we observed that **GP@Gel Nap‐T** was even more cytotoxic to both cell lines than free PTX and GDC0919&PTX, indicating the potential of the peptide‐based hydrogel to mitigate severe toxic effects caused by PTX (**Figure** **2**a). To investigate whether the observed cytotoxicity was induced by apoptosis, we used Annexin V‐fluorescein isothiocyanate and propidium iodide (PI) costaining on treated cells (Figure 2b,c; Figure S31, Supporting Information). PTX and GDC0919&PTX induced early/late apoptosis of 12.3 ± 0.6% and 12.7 ± 0.2% in A549 cells, 14.0 ± 0.2% and 14.3 ± 0.4% in CMT167 cells, respectively. Conversely, the **P@Gel Nap‐T** and **GP@Gel Nap‐T** groups exhibited higher apoptosis rates compared to the other groups (Figure 2b,c; Figure S31, Supporting Information), suggesting their high cytotoxicity to NSCLC cells. These results suggested that PTX in our hydrogel provided enhanced NSCLC cell apoptosis.

We then investigated the anti‐metastasis efficacy of **GP@Gel Nap‐T** through scratch wound healing (Figure 2d,e) and transwell migration assays (Figure 2f,g). A549 or CMT167 cells incubated with PBS, **Gel Nap‐T**, GDC0919, or **G@Gel Nap‐T** exhibited obvious wound healing over 24 h (Figure 2e; Figure S32, Supporting Information), demonstrating the aggressive motility of NSCLC cells in these PTX‐absent groups. In contrast, A549 cells treated with PTX, GDC0919&PTX, **P@Gel Nap‐T**, and **GP@Gel Nap‐T**, exhibited slow healing rates of 51.86 ± 2.5%, 49.97 ± 3.3%, 34 ± 4.6%, and 31.9 ± 5.8% at 24 h, respectively, while those of CMT167 cells were 50.34 ± 1.9%, 50.16 ± 2.3%, 26.5 ± 1.6%, and 26.2 ± 1.82% at 24 h, respectively (Figure 2e; Figure S32, Supporting Information). These results demonstrated the significantly inhibited scratch wound healing of NSCLC cells in these PTX‐present groups, which was primarily attributed to the anti‐metastasis ability of PTX. Notably, among these groups, the “**P@Gel Nap‐T**” and “**GP@Gel Nap‐T**” group showed the best suppression on wound healing in both A549 and CMT167 cell models. Moreover, the transwell migration assays revealed that the “**P@Gel Nap‐T**” and “**GP@Gel Nap‐T**” group exhibited the lowest cell migration than the other groups in both cell models (Figure 2g; Figure S33, Supporting Information). Specifically, A549 cells treated with **P@Gel Nap‐T** and **GP@Gel Nap‐T** exhibited the slowest migration rates of 55.9 ± 1.2% and 57.4 ± 1.4%, respectively, at 48 h. Similarly, CMT167 cells with these treatments also showed the slowest migration rates of 36.3 ± 2.1% and 36.5 ± 2.6%, respectively (Figure 2g; Figure S33, Supporting Information). Collectively, these results demonstrated the superior anti‐metastasis ability of **GP@Gel Nap‐T**, which is mediated by its potent PTX component.

Next, we further explored the possible underlying anti‐metastasis mechanism of **GP@Gel Nap‐T**. Considering that autophagy might play a crucial role in PTX‐induced antimetastasis,^[^ ^23^ ^]^ we thus investigated the expression of autophagy‐associated proteins (i.e., LC3‐II, Beclin‐1, p62) and the upstream proteins (i.e., p‐AKT) using western blotting (WB) analysis. Compared with those PTX‐absent groups (i.e., “PBS”, “**Gel Nap‐T**”, “GDC0919”, and “**G@Gel Nap‐T**”), the PTX‐present groups (i.e., “PTX”, “GDC0919&PTX”, “**P@Gel Nap‐T**”, and “**GP@Gel Nap‐T**”), particularly the “**P@Gel Nap‐T**” and “**GP@Gel Nap‐T**” groups, showed increased expression of LC3‐II and Beclin‐1 while decreased expression of p‐AKT and p62 in both A549 and CMT167 cell models (Figure 2h), indicating the PTX‐mediated autophagic cell death through the AKT signaling pathway. These results also indicated that the hydrogel **GP@Gel Nap‐T** enhanced PTX‐mediated autophagic cell death to improve anti‐metastasis.

Proliferation constitutes a crucial aspect of cancer development and progression.^[^ ^24^ ^]^ Consequently, we examined the impact of **GP@Gel Nap‐T** on the proliferation in both cell models through the colony formation assay (Figure 2i,j). Compared with those of the other groups, A549 and CMT167 cells treated with **GP@Gel Nap‐T** formed the fewest and smallest colonies of 13.9% and 10.0%, respectively (Figure 2j; Figure S34, Supporting Information), demonstrating the high antiproliferation efficacy of **GP@Gel Nap‐T** in NSCLC cell models. The diminished proliferation might be associated with an arrest in certain stages of the cell cycle.^[^ ^25^ ^]^ Therefore, we further characterized the effects of **GP@Gel Nap‐T** on cell cycle progression using flow cytometry (FCM) analysis. A549 or CMT167 cells treated with PBS, **Gel Nap‐T**, GDC0919, or **G@Gel Nap‐T** remained a normal cell cycle (Figure 2k,l; Figure S35, Supporting Information). In contrast, in those PTX‐present groups (i.e., “PTX”, “GDC0919&PTX”, “**P@Gel Nap‐T**”, and “**GP@Gel Nap‐T**”), an increased proportion of G2 phase cells was observed at 48 h post treatment (Figure 2k,l; Figure S35, Supporting Information), indicating a G2 phase blocking induced by PTX.^[^ ^26^ ^]^ Notably, this proportion was significantly larger for those cells treated with **GP@Gel Nap‐T** or **P@Gel Nap‐T** at 48 h than those of PTX‐dissociative groups (i.e., “PTX” and “GDC0919&PTX”). This was probably because the hydrogel platform provided protection on PTX and sustainably released PTX for persistent cell cycle arrest. Collectively, these results demonstrated the high anti‐proliferation efficiency of **GP@Gel Nap‐T** through persistent PTX‐induced NSCLC cell cycle arrest.

Taken together, the above findings in this section implied that the sustained release of PTX from **GP@Gel Nap‐T** hindered cell metastasis via the AKT/autophagy pathway (i.e., LC3‐II and Beclin‐1 upregulated, p‐AKT and p62 downregulated), and curtailed cell proliferation through cell cycle arrest at the G2 stage. Ultimately, these mechanisms cocontributed to induction of cellular demise (Figure 2m).

### 2.6 GP@Gel Nap‐T Enhances Immune Response through ICD Induction and IDO Inhibition In Vitro

**Figure 3:**
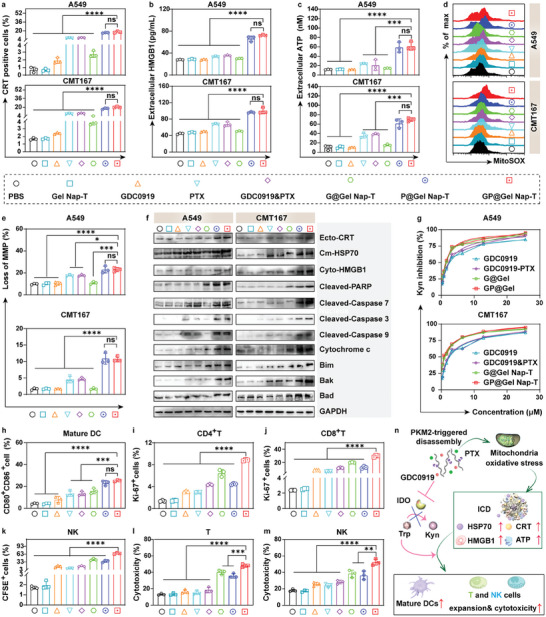
The efficiency and mechanism of ICD induction, IDO enzyme activity inhibition, and immune response elicited by **GP@Gel Nap‐T** in vitro. a) Quantitative FCM analysis of CRT on (up) A549 and (down) CMT167 cells after treatment with each formulation for 12 h (*n* = 3, biological independent samples). b) ELISA quantitative analysis of the release of HMGB1 from (up) A549 and (down) CMT167 cells after treatment with each formulation for 12 h (*n* = 3, biological independent samples). c) Quantitative analysis of extracellular ATP in (up) A549 and (down) CMT167 cells after treatment with each formulation for 12 h (*n* = 3, biological independent samples). d) Representative FCM analysis of MitoSOX in (up) A549 and (down) CMT167 cells after treatment with each formulation for 48 h (*n* = 3, biological independent samples). e) Quantitative FCM analysis of MMP in (up) A549 and (down) CMT167 cells after treatment with each formulation for 48 h (*n* = 3, biological independent samples). f) WB results of ICD‐associated proteins and its upstream proteins expression in (left) A549 and (right) CMT167 cells after treatment with each formulation for 48 h. Control, GAPDH (*n* = 3, biological independent samples). g) Inhibitory effects of the IDO‐mediated Kyn production on (up) A549 and (down) CMT167 cells after treatment with GDC0919, GDC0919&PTX, **G@ Gel Nap‐T**, and **GP@ Gel Nap‐T** for 48 h (*n* = 3, biological independent samples). h) Quantitative FCM analysis of mature DCs (*n* = 3, biological independent samples). i–k) Quantitative FCM analysis of (i) CD4^+^ T cells, (j) CD8^+^ T cells and (k) NK cells proliferation (*n* = 3, biological independent samples). l,m) Quantitative FCM analysis of dead CMT167 cells after (l) T cell and (m) NK cell attacking (*n* = 3, biological independent samples). n) Schematic illustration for the immune regulatory effect of **GP@Gel Nap‐T** via PTX‐induced mitochondrial oxidative stress‐ICD and GDC0919‐inhibited Kyn production in vitro (*n* = 3, biological independent samples). Results are presented as mean ± SD. Statistical significance was assessed using one‐way ANOVA with Tukey's post‐test. ns: no significant difference, *P < 0.05, **P < 0.01, ***P < 0.001, ****P < 0.0001.

Previous studies revealed the ability of PTX in inducing ICD across various tumor cells.^[^ ^4^, ^27^ ^]^ We then validated the immunogenic effect of **GP@Gel Nap‐T** in A549 and CMT167 cell models. After treating cells with **GP@Gel Nap‐T** or the other counterparts, we meticulously monitored the profiles of some representative DAMPs, including CRT, HMGB1, and ATP. As expected, we found that in both A549 and CMT167 cell models, the PTX‐present groups (i.e., “PTX”, “GDC0919&PTX”, “**P@Gel Nap‐T**”, and “**GP@Gel Nap‐T**”) showed much higher levels of CRT (**Figure** **3**a; Figure S36, Supporting Information), HMGB1 (Figure 3b), and ATP (Figure 3c) than the PTX‐absent groups (i.e., “PBS”, “**Gel Nap‐T**”, “GDC0919”, and “**G@Gel Nap‐T**”) in tumor cells, suggesting the ICD effect caused by PTX. Notably, the levels of the above DAMPs were significantly higher in PTX‐in‐gel groups (i.e., “**P@Gel Nap‐T**” and “**GP@Gel Nap‐T**”) over in PTX‐dissociative groups (i.e., “PTX” and “GDC0919&PTX”), implying that the sustained release of PTX for hydrogel enhanced the ICD effect.

Considering the crucial role of ROS upregulation and mitochondrial oxidative stress in PTX‐caused apoptosis and the release of DAMPs,^[^ ^28^ ^]^ we then investigated the intracellular ROS levels and mitochondrial‐related cues upon various treatment in A549 and CMT167 cell models. An ROS probe DCFH‐DA, mitochondrial superoxide probe MitoSOX and an MMP indicator JC‐1 were employed to facilitate FCM and CLSM analyses. We found that compared with the PTX‐absent groups (i.e., “PBS”, “**Gel Nap‐T**”, “GDC0919”, and “**G@Gel Nap‐T**”), all the PTX‐present groups (i.e., “PTX”, “GDC0919&PTX”, “**P@Gel Nap‐T**”, and “**GP@Gel Nap‐T**”) showed higher intracellular ROS levels (Figures S37 and S38, Supporting Information) and higher mitochondrial superoxide levels (Figure 3d; Figure S39, Supporting Information), and bigger MMP loss (Figure 3e; Figure S40, Supporting Information). GSH stands as a pivotal component of intracellular antioxidant mechanisms. Typically, cancer cells generate GSH in response to elevated ROS levels, aiming to uphold the delicate mitochondrial redox equilibrium. Consequently, we delved deeper into the impact of **GP@Gel Nap‐T** on mitochondrial GSH concentrations. As anticipated, all the PTX‐present groups (i.e., “PTX”, “GDC0919&PTX”, “**P@Gel Nap‐T**”, and “**GP@Gel Nap‐T**”) showed stronger GSH depletion (Figure S41, Supporting Information) in both cancer cell models. These results indicated the PTX‐caused oxidative stress, which were particularly severe for PTX‐in‐gel groups (i.e., “**P@Gel Nap‐T**” and “**GP@Gel Nap‐T**”). We further performed WB analysis and found that both A549 and CMT167 cells in PTX‐in‐gel groups exhibited significantly higher levels of DMAPs (i.e., surface‐exposed CRT (Ecto‐CRT), cell membrane HSP70 (Cm‐HSP70), and cytoplasm HMGB1 (Cyto‐HMGB1)), apoptotic proteins (i.e., Cleaved‐PARP, Cleaved‐caspase 3/7/9, Bim, Bak, and Bad) and cytochrome c than the other groups (Figure 3f), which indicated ICD and anabatic cell apoptosis for PTX‐in‐gel groups (i.e., “**P@Gel Nap‐T**” and “**GP@Gel Nap‐T**”).

As with previous reports,^[^ ^11a^ ^]^ enzyme‐linked immunosorbent assay (ELISA) and WB results showed that PTX significantly upregulated both IFN‐γ and IDO expression levels in CMT167‐bearing tumor tissues (Figures S42 and S43, Supporting Information). We also demonstrated a pronounced elevation in IDO expression in vitro within A549, NCIH1975, and CMT167 cells under IFN‐γ conditions (Figure S44, Supporting Information). Analysis of the correlation between IDO expression and immune cell infiltration in the TCGA database (Figure S45, Supporting Information) strongly suggested the involvement in orchestrating CD8^+^ T (CD3^+^CD8^+^) cells, CD4^+^ T (CD3^+^CD4^+^) cells, Tregs (CD4^+^Foxp3^+^), macrophages (CD68^+^), M1‐TAMs (CD68^+^CD80^+^ or CD68^+^CD86^+^), M2‐TAMs (CD68^+^CD163^+^), DCs (CD11c^+^), and MDSCs (CD11b^+^CD14^+^) infiltration within the tumor milieu. We found that GDC0919‐in‐gel groups (i.e., “**G@Gel Nap‐T**” and “**GP@Gel Nap‐T**”) exhibited nearly the same Kyn inhibition profile with an EC_50_ value of 1.81 ± 0.09 µm, which was higher than that of GDC0919‐dissociative groups (i.e., “GDC0919” and “GDC0919&PTX”) in both A549 and CMT167 cell models (Figure 3g), implying that our hydrogel could provide improved blocking of IDO activity through the sustained release of GDC0919 for persistent inhibition of Kyn production. Taken together, these results suggested that our PKM2‐responsive hydrogel enabled amplified mitochondrial oxidative stress, apoptotic cell death and downstream ICD, as well as improved inhibition of IDO activity, through sustained release of PTX and GDC0919.

Considering that ICD promoted the maturation of DCs, we then investigated the DCs after treatment with **GP@Gel Nap‐T** in vitro. CMT167 cells were treated with **GP@Gel Nap‐T** or the other counterparts for 12 h. Then, bone marrow‐derived dendritic cells (BMDCs) isolated from C57BL/6 mice and stimulated by recombinant murine GM‐CSF, recombinant murine IL‐4 for 7 days to acquire immature DCs were cultured with pretreated CMT167 cells for another 24 h, followed by analysis of the percentage of mature DCs (CD80^+^CD86^+^) using FCM. The percentages of mature DCs in PTX‐absent groups (i.e., “PBS”, “**Gel Nap‐T**”, “GDC0919”, and “**G@Gel Nap‐T**”) were low (3.05 ± 0.14%, 3.98 ± 0.19%, 9.2 ± 0.25%, and 16.43 ± 0.3%, respectively), while those of PTX‐dissociative groups (i.e., “PTX” and “GDC0919&PTX”) were a little higher (12.2 ± 0.25% and 16.43 ± 0.3%, respectively) (Figure 3h; Figure S46, Supporting Information). In contrast, the PTX‐in‐gel groups (i.e., “**P@Gel Nap‐T**” and “**GP@Gel Nap‐T**”) exhibited the highest percentages of mature DCs (20.2 ± 0.5% and 26.43 ± 0.7%, respectively), which were significantly higher than those of the above groups, demonstrating that our hydrogel platform promoted the PTX‐induced phenotypic maturation of DCs.

Next, we validated whether our **GP@Gel Nap‐T** could enhance proliferation of T cells and NK cells, as well as the corresponding tumor cytotoxicity. CMT167 cells were stimulated with 50 ng mL^−1^ recombinant murine IFN‐γ to induce IDO expression and then treated **GP@Gel Nap‐T** or the other treatments for 12 h before co‐culture. Pretreated CMT167 cells were cocultured with splenic lymphocytes isolated from C57BL/6 mice in the presence of a soluble anti‐CD3 antibody and recombinant murine IL‐2 for another 48 h, followed by assessment of CD4^+^ T and CD8^+^ T cells using FCM. For evaluation of NK cells, recombinant murine IL‐15 was added along with recombinant murine IL‐2 and prestained with 5‐(and 6)‐carboxyfluorescein diacetate (CFSE) when coculturing cells. We found that, the “**GP@Gel Nap‐T**” group exhibited the highest levels of CD4^+^ T cells (Figure 3i; Figure S47, Supporting Information), CD8^+^ T cells (Figure 3j; Figure S48, Supporting Information) and CFSE^+^ NK cells (Figure 3k; Figure S49, Supporting Information) over the other groups, demonstrating the superior proliferation of T cells and NK cells of **GP@Gel Nap‐T** through IDO inhibition by GDC0909 and ICD induction by PTX.

We then evaluated the tumor‐attacking capability of the activated T and NK cells. To effectively distinguish between effector and target cells, the recombinant murine IFN‐γ‐treated CMT167 target cells underwent pre‐treatment with **GP@Gel Nap‐T** for 12 h and were subsequently labeled with CFSE. Following this, they were incubated with effector splenic lymphocytes at a ratio of 1:10 for 4 h. To assess the percentage of killed target cells, 7‐AAD was introduced into the cell suspensions, and FCM analysis was conducted to determine the proportion of 7‐AAD‐positive CMT167 cells. We found that splenic lymphocytes isolated from C57BL/6 mice in the presence of a soluble anti‐CD3 antibody and recombinant murine IL‐2 for 3 days, the “**GP@Gel Nap‐T**” group leaded to a significantly higher percentage of cancer cell death (50.4 ± 1.14%) than those from the other groups (less than 40%) (Figure 3l; Figure S50, Supporting Information). Similarly, the splenic lymphocytes isolated from C57BL/6 mice and activated by recombinant murine IL‐15 and recombinant murine IL‐2 from the “**GP@Gel Nap‐T**” group exhibited a high percentage of cell death (55.0 ± 1.21%), which was also significantly higher than those of the other groups (less than 40%) (Figure 3m; Figure S51, Supporting Information). Collectively, these results suggested that our PKM2‐responsive hydrogel was able to improve proliferation of T cells and NK cells as well as tumor cytotoxicity through ICD induction and IDO inhibition, thus rendering enhanced immune response (Figure 3n).

### 2.7 Enhanced Antitumor Efficacy of GP@Gel Nap‐T In Vivo

**Figure 4:**
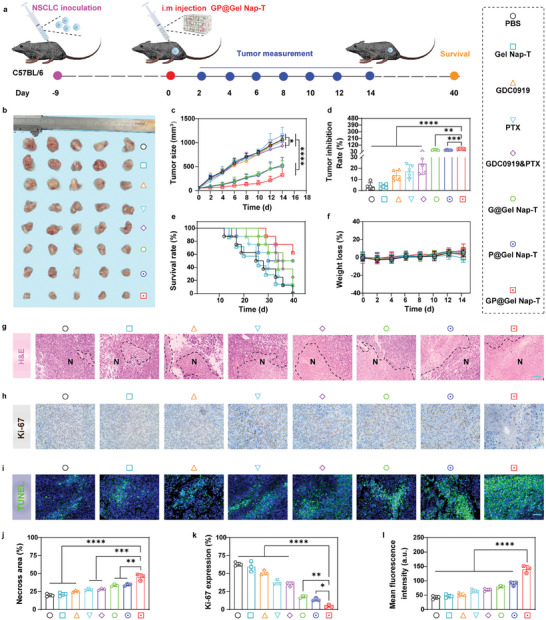
In vivo evaluations of antitumor effects of **GP@Gel Nap‐T**. a) Schedule of tumor implantation, **GP@Gel Nap‐T** injection, tumor measurement, and survival analysis. b) Photographs of tumors on day 14 after different treatments (*n* = 5 biologically independent animals per group). c) Average tumor growth kinetics. Growth curves were stopped when the tumor volume of the first mouse in the corresponding group reach 1500 mm^3^ (*n* = 5 biologically independent animals per group). d) Tumor growth inhibition of different treatments (*n* = 5 biologically independent animals per group). e) Survival of the mice on day 40 after different treatments (*n* = 5 biologically independent animals per group). f) Body weight loss of the CMT167 tumor‐bearing mice after different treatments (*n* = 5 biologically independent animals per group). g–i) Representative image of (g) H&E staining, (h) Ki‐67 staining, and (i) TUNEL staining in the tumor tissue from CMT167 tumor‐bearing mice on day 14 after different treatments. The letter “N” in the images represents the necrotic area. DAPI (blue), apoptosis nuclei debris (green). Scale bars: 50 µm (*n* = 3, biological independent samples). j–l) Corresponding quantitative analysis of (j) necrotic area percent of tumor tissues in (g), (k) the Ki‐67 expressions in (h), and (l) TUNEL signal area in (i) (*n* = 3 biological independent samples). Results are presented as mean ± SD. Statistical significance was assessed using one‐way ANOVA with Tukey's post‐test. ns: no significant difference, *P < 0.05, **P < 0.01, ***P < 0.001, ****P < 0.0001.

We next assessed the antitumor efficacy of **GP@Gel Nap‐T** in vivo using a CMT167 tumor‐bearing mouse model. First, C57BL/6 mice were inoculated with CMT167 cells (i.e., 1 × 10^6^ cells per gland) on the right back flank to establish subcutaneous tumors. After 9 days, these CMT167‐bearing mice were randomly divided into 8 groups (*n* = 5) and intratumorally administered with **GP@Gel Nap‐T** or the other seven counterparts (i.e., PBS, **Gel Nap‐T**, GDC0919, PTX, GDC0919&PTX, **G@Nap‐T**, and **P@Nap‐T**), followed by tumor measurement every two days for 14 days and survival time was recorded as 40 days (**Figure** **4**a). On day 14 post drug injection, we found that the groups “**Gel Nap‐T”**, “GDC0919”, “PTX”, and “GDC0919&PTX” exhibited similar tumor sizes (Figure 4b), as well as the time‐dependent size increment profiles (Figure 4c; Figure S52, Supporting Information), with the “PBS” group, indicating the inferior tumor inhibition efficacy of these groups. In contrast, tumors in drug‐in‐gel groups (i.e., “**G@Nap‐T**”, “**P@Nap‐T**”, and “**GP@Nap‐T**”) showed significantly smaller sizes with slower size increase rates, and notably, the “**GP@Nap‐T**” group showed the smallest tumor sizes, and the slowest tumor growth rate (Figure 4b,c; Figure S52, Supporting Information). To facilitate comparison, we further calculated the tumor inhibition rates of the above eight groups: “PBS” (6.8%), “**Gel Nap‐T**” (7.4%), “GDC0919” (11.2%), “PTX” (13.6%), “GDC0919&PTX” (23.7%), “**G@Nap‐T**” (53.3%), “**P@Nap‐T**” (53.0%), and “**GP@Nap‐T**” (73.1%), demonstrating the superior antitumor efficacy of the drug‐in‐gel groups (particularly, the “**GP@Nap‐T**” group) (Figure 4d). Moreover, 40% of the mice survived beyond 40 days after **GP@Nap‐T** treatment (Figure 4e), demonstrating the highest survival rate. We also confirmed the biosafety of **GP@Nap‐T** by monitoring weights of mice, conducting serum enzyme assays of mouse blood samples, and performing histopathological analysis of major organs and skin of mice on day 14. During the observation period of 14 days, no notable body weight fluctuations, abnormalities and organ damages, serum biochemistry markers and blood cell profile of mice in the above eight groups were observed (Figure 4f; Figure S53, Supporting Information), suggesting negligible adverse effect of **GP@Nap‐T** and its counterparts. Subsequently, hematoxylin & eosin (H&E) analysis, Ki‐67 staining, and immunohistochemical (IHC) assay of tumor tissues were performed. H&E analysis revealed that tumors of mice in “**GP@Nap‐T**” group displayed the most pyknotic cells (45.17%) with highly condensed nuclei, indicating the most apoptotic or dead tumor cells upon **GP@Nap‐T** treatment (Figure 4g,j). The weakest Ki‐67 staining image observed in “**GP@Nap‐T**” group suggested that **GP@Nap‐T** treatment leaded to the least proliferative tumor cells (4.94%) (Figure 4h,k). Besides, a significantly higher mean fluorescence intensity of dUTP nick‐end labeling (TUNEL)‐positive tumor cells were observed in “**GP@Nap‐T**” group (140.44 au) compared to the other groups (less than 90 au), indicating the most enhanced tumor cell apoptosis upon **GP@Nap‐T** treatment (Figure 4i,l). Taken together, the above results suggested that our hydrogel platform with controlled release of PTX and GDC0919 could significantly improve the inhibition on tumor growth in vivo.

### 2.8 Enhanced ICD Induction and IDO Inhibition by GP@Nap‐T In Vivo

**Figure 5:**
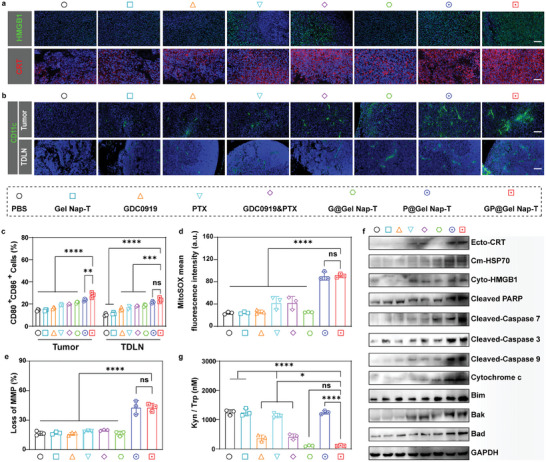
ICD induction and IDO inhibition by **GP@Gel Nap‐T** in vivo. a) Representative IF staining of HMGB1 and CRT in the tumor tissues from CMT167 tumor‐bearing mice on day 14 after different treatments. DAPI (blue), HMGB1 (green), CRT (red). Scale bars: 50 µm (*n* = 3 biological independent samples). b) Representative IF staining of DCs in the tumor tissues and TDLNs from CMT167 tumor‐bearing mice on day 14 after different treatments. DAPI (blue), CD11c (green). Scale bars: 50 µm (*n* = 3 biological independent samples). c) Quantitative FCM analysis of mature DCs in the tumor tissues and TDLNs from CMT167 tumor‐bearing mice on day 14 after different treatments (*n* = 3 biological independent samples). d) Quantitative FCM analysis of MitoSOX levels in the tumor tissues from CMT167 tumor‐bearing mice on day 14 after different treatments (*n* = 3 biological independent samples). e) Quantitative FCM analysis of MMP of tumor cells from CMT167 tumor‐bearing mice on day 14 (*n* = 3 biological independent samples). f) WB results of ICD‐associated proteins and its upstream proteins expression in the tumor tissues from CMT167 tumor‐bearing mice on day 14 after different treatments. Control, GAPDH (*n* = 3 biological independent samples). g) The ratio of Kyn/Trp concentrations in tumor from CMT167 tumor‐bearing mice on day 14 after different treatments (*n* = 3 biological independent samples). Results are presented as mean ± SD. Statistical significance was assessed using one‐way ANOVA with Tukey's post‐test. ns: no significant difference, *P < 0.05, **P < 0.01, ***P < 0.001, ****P < 0.0001.

We then validated whether the enhanced antitumor efficacy of **GP@Gel Nap‐T** was ascribed to the improved ICD and IDO inhibition in CMT167 tumor‐bearing mouse model. Immunofluorescence (IF) staining was first conducted to examine the expression of HMGB1 and CRT in tumor tissues from CMT167 tumor‐bearing mice on day 14 post various treatments. Remarkably, noticeably higher fluorescence signals of HMGB1 and CRT were observed in tumor tissues from **GP@Gel Nap‐T**‐treated mice (**Figure** **5**a; Figure S54, Supporting Information) than those of the other groups. In consistent with that in cell experiments, this result indicated that much more DAMPs were released to induce ICD after **GP@Gel Nap‐T** treatment. Moreover, we also observed that tumor tissues of mice in “**GP@Gel Nap‐T**” group exhibited enhanced recruitment of DCs into tumors and TDLNs (Figure 5b; Figure S55, Supporting Information), and these recruited DCs in “**GP@Gel Nap‐T**” group showed higher levels of CD80^+^ and CD86^+^ (Figure 5c; Figure S56, Supporting Information). These results suggested the enhanced ICD effect in vivo upon **GP@Gel Nap‐T** treatment. The crucial cellular species/event involved in ICD provoking pathway were also investigated in tumor tissues from CMT167 tumor‐bearing mice on day 14 post various treatments, such as ROS level, mitochondrial oxidative stress, and apoptotic proteins. Using DCFH‐DA as the indicator, ROS levels of tumor tissues quantified by FCM analysis revealed that the “**GP@Gel Nap‐T**” group exhibited the highest ROS level in tumor tissues among the groups (Figure S57, Supporting Information). Similar IF results were obtained in the mean fluorescence intensity values of tumor cells separately treated with **GP@Gel Nap‐T** (Figure S58, Supporting Information). Moreover, the “**GP@Gel Nap‐T**” group, as well as the “**P@Gel Nap‐T**” group, showed a significantly higher MitoSOX levels (Figure 5d; Figure S59, Supporting Information) and MMP loss (Figure 5e; Figure S60, Supporting Information) while lower mitochondrial GSH level (Figure S61, Supporting Information) than the other control groups, demonstrating heavier mitochondrial oxidative stress for the PTX‐in‐gel groups (i.e., “**P@Gel Nap‐T**” and “**GP@Gel Nap‐T**”). It is recognized that abnormal mitochondria subsequently release cytochrome c from its intermembrane space to activate pro‐apoptotic pathways (hallmarks: Bim, Bak, and Bad) and caspase signaling pathways (hallmarks: Cleaved‐PARP, Cleaved‐caspase 7, Cleaved‐caspase 3, Cleaved‐caspase 9), which culminate in ICD. As expected, WB results indicated significantly overexpression of these hallmarks as well as DMAPs (Ecto‐CRT, Cm‐HSP70, Cyto‐HMGB1) in tumor tissues from CMT167 tumor‐bearing mice on day 14 post treatments with **P@Gel Nap‐T** and **GP@Gel Nap‐T** (Figure 5f). Notably, despite the “**GP@Gel Nap‐T**” group exhibited similar potency in some aspects with “**P@Gel Nap‐T**”, the former showed significantly higher overall anticancer efficacy than the latter in vivo, as demonstrated in Section 2.7, owing to the synergistic effect of PTX and GDC0919. Collectively, these results indicated that **GP@Gel Nap‐T** enabled enhanced ICD effect in vivo through improvements in mitochondrial oxidative stress, DAMPs release, DC recruitment and maturation within tumor tissues.

In addition, we also validated the IDO inhibition by **GP@Gel Nap‐T** in vivo through analyzing the contents of Kyn and Trp in tumor tissues or serum with HPLC. In tumor tissues from CMT167 tumor‐bearing mice on day 14 post various treatments, the GDC0919‐present groups (i.e., “GDC0919”, “GDC0919&PTX”, “**G@Gel Nap‐T**” and “**GP@Gel Nap‐T**”) showed significantly lower Kyn/Trp ratios than the GDC0919‐absent groups (i.e., “PBS”, “**Gel Nap‐T**”, “PTX”, and “**P@Gel Nap‐T**”) (Figure 5g), demonstrating GDC0919‐induced downregulation of immunosuppressive Kyn. Notably, among the GDC0919‐present groups, the GDC0919‐in‐gel groups (i.e., “**G@Gel Nap‐T**” and “**GP@Gel Nap‐T**”) exhibited improved inhibition of IDO activity over the GDC0919‐dissociative groups (i.e., “GDC0919” and “GDC0919&PTX”). Similar results were observed in serum samples as well (Figure S62, Supporting Information). These results demonstrated that our hydrogel with sustained release of GDC0919 provided enhanced IDO inhibition in vivo.

### 2.9 Enhanced Immune Response by GP@Gel Nap‐T In Vivo

**Figure 6:**
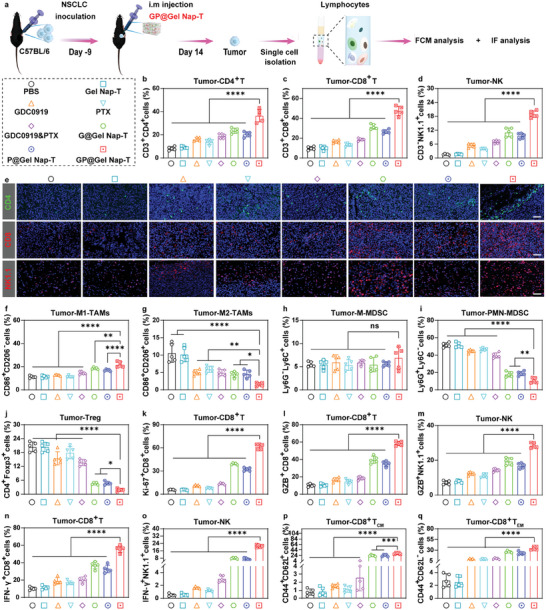
Immune activation by **GP@ Gel Nap‐T** in tumor tissues in vivo. a) Schematic illustration about the workflow of FCM and IF analysis of tumor tissues from CMT167 tumor‐bearing mice on day 14 after different treatments. b–d) Quantitative FCM analysis of the proportions of (b) CD4^+^ T cells, (c) CD8^+^ T cells, (d) NK cells in the tumor tissues from CMT167 tumor‐bearing mice on day 14 after different treatments (*n* = 5 biological independent samples). e) Representative IF staining of CD4, CD8, and NK1.1 in the tumor tissues from CMT167 tumor‐bearing mice on day 14 after different treatments. DAPI (blue), CD4 (green), CD8, and NK1.1 (red). Scale bars: 50 µm (*n* = 3 biological independent samples). f–q) Quantitative FCM analysis of the proportions of (f) M1‐TAMs, (g) M2‐TAMs, (h) M‐MDSCs, (i) PMN‐MDSCs, (j) Tregs, (k) Ki‐67^+^CD8^+^ T cells, (l) GZB^+^CD8^+^ T cells, (m) GZB^+^NK1.1^+^ cells, (n) IFN‐γ^+^CD8^+^ T cells, (o) IFN‐γ^+^NK1.1^+^ cells, (p) CD8^+^ T_CM_ (CD8^+^CD44^+^CD62L^+^) cells, and (q) CD8^+^ T_EM_ (CD8^+^CD44^+^CD62L^−^) in the tumor tissues from CMT167 tumor‐bearing mice on day 14 after different treatments (*n* = 5 biological independent samples). Results are presented as mean ± SD. Statistical significance was assessed using one‐way ANOVA with Tukey's post‐test. ns: no significant difference, *P < 0.05, **P < 0.01, ***P < 0.001, ****P < 0.0001.

To reveal the immune response mediated by **GP@Gel Nap‐T** in vivo, we analyzed immune cell populations in tumor tissues from CMT167 tumor‐bearing mice on day 14 post treatment (**Figure** **6**a). Compared with the other seven groups, the “**GP@Gel Nap‐T**” group showed significantly higher percentages of tumor‐infiltrating CD4^+^ T (Figure 6b), CD8^+^ T (Figure 6c), and NK1.1^+^ cells (Figure 6d) in tumor tissues, which were also demonstrated by IF staining (Figure 6e; Figure S63, Supporting Information). Notably, the other two drug‐in‐gel groups (i.e., “**G@Gel Nap‐T**” and “**P@Gel Nap‐T**”) also showed higher percentages of CD4^+^ T, CD8^+^ T, and NK1.1^+^ cells than the other groups (i.e., “PBS”, “**Gel Nap‐T**”, “GDC0919”, “PTX”, and “GDC0919&PTX”), demonstrating the immune cell‐provoking potential of drug delivery based on hydrogel. Moreover, the **GP@Gel Nap‐T** treatment leaded to the highest percentage of antitumorigenic M1‐TAMs (Figure 6f) while lowest population of protumorigenic M2‐TAMs (Figure 6g), implying that **GP@Gel Nap‐T** may effectively modulate the polarization state of TAMs. The observed result can be attributed to two profound mechanisms: first, PTX exhibits the capacity to reprogram M2‐TAMs into M1‐TAMs, thereby activating the immune response, as evidenced in previous studies;^[^ ^29^ ^]^ second, the downregulation of IDO achieved by GDC0919 appears to correlate significantly with a diminished proportion of the M2 phenotype among TAMs.^[^ ^30^ ^]^ The proportion of monocytic myeloid‐derived suppressor cells (M‐MDSCs) in tumor tissues showed no significant difference among all groups (Figure 6h), suggesting that the treatment did not have a substantial impact on the abundance of M‐MDSCs in the TME. The percentages of polymorphonuclear myeloid‐derived suppressor cells (PMN‐MDSCs) and Tregs in “**GP@Gel Nap‐T**” group significantly decreased to 8.4% and 2.9%, respectively, ≈6.1 and 7.2 times lower than those in “PBS” group (51.63% and 20.44%, respectively), respectively (Figure 6i,j). This finding is in line with previous reports, which indicate that the codelivery of an IDO inhibitor and PTX results in a decrease in the population of MDSCs and Tregs.^[^ ^31^ ^]^ Collectively, these results indicated that **GP@Gel Nap‐T** treatment not only enriched T cells, NK cells, and M1‐TAMs in tumor tissues but also downregulated the populations of M2‐TAMs, PMN‐MDSCs, and Tregs, synergistically enhancing antitumor immune response.

Next, we undertook a thorough examination of T cell proliferation within tumors belonging to these distinct groups. Our findings highlighted a significant elevation in the percentage of Ki‐67^+^CD8^+^ T cells and Ki‐67^+^CD4^+^ T cells within tumors following **GP@Nap‐T** treatment (Figure 6k; Figure S64, Supporting Information). To delve deeper into the immune response at tumor sites, we further measured the levels of representative pro‐inflammatory and anti‐inflammatory cytokines in tumor tissues from CMT167 tumor‐bearing mice on day 14 post treatment using FCM and ELISA analyses. Compared with the other seven groups, the “**GP@Gel Nap‐T**” group exhibited the highest percentages of granzyme B (GZB)^+^CD4^+^ T cells (Figure S65, Supporting Information), GZB^+^CD8^+^ T cells (Figure 6l), and GZB^+^NK1.1^+^ cells (Figure 6m), as well as IFN‐γ^+^CD4^+^ T (Figure S66, Supporting Information), IFN‐γ^+^CD8^+^ T (Figure 6n), and IFN‐γ^+^NK1.1^+^ cells (Figure 6o). Additionally, the cytokines levels in tumor tissues, such as IL‐10, TNF‐α, and IFN‐γ, were measured by ELISA. IL‐10, known for inhibiting T cell and macrophage activation, and promoting the differentiation of immunosuppressive Tregs, was found to be downregulated upon **GP@Gel Nap‐T** treatment (Figure S67a,b, Supporting Information). Conversely, TNF‐α and IFN‐γ showed the highest levels in “**GP@Gel Nap‐T**” group (Figure S67a,c,d, Supporting Information), indicating the enhanced upregulation of the immuno‐promoting cytokines.

To validate whether the activation of immune responses generated memory immune effects, we measured the amount of central memory T (T_CM_, CD44^+^CD62L^+^) cells and effector memory T (T_EM_, CD44^+^CD62L^−^) cells in tumor tissues from CMT167 tumor‐bearing mice on day 14 post treatment using FCM analyses. Consistent with the improved antitumor immune responses in tumors, **GP@Gel Nap‐T** treatment predominantly resulted in the highest frequency of CD4^+^ T_CM_ and CD8^+^ T_CM_ cells in the tumor tissues compared to the other groups (Figure 6p; Figures S68a and S69, Supporting Information). Additionally, **G@Gel Nap‐T** or **P@Gel Nap‐T** modestly increased the percentage of CD4^+^ T_CM_ and CD8^+^ T_CM_ cells when compared with those in the control mice (Figure 6p; Figures S68a and S69, Supporting Information). The percentage of CD4^+^ T_EM_ cells and CD8^+^ T_EM_ cells significantly increased in tumors after **GP@Gel Nap‐T** treatment (Figure 6q; Figures S68b and S69, Supporting Information), suggesting the capability of generating long‐term protection against tumor burdens, metastasis, or relapse.

### 2.10 GP@Gel Nap‐T Amplifies Antitumor Immune Responses in TDLNs

**Figure 7:**
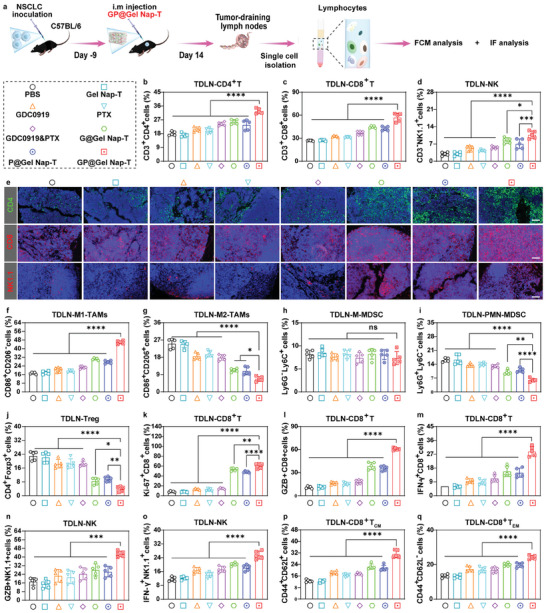
Immune activation by **GP@Gel Nap‐T** in TDLNs in vivo. a) Schematic illustration about the workflow of FCM and IF analysis of TDLNs from CMT167 tumor‐bearing mice on day 14 after different treatments. b–d) Quantitative FCM analysis of the proportions of (b) CD4^+^ T cells, (c) CD8^+^ T cells, (d) NK cells in TDLNs from CMT167 tumor‐bearing mice on day 14 after different treatments (*n* = 5 biological independent samples). e) Representative IF staining of CD4, CD8, and NK1.1 in TDLNs from CMT167 tumor‐bearing mice on day 14 after different treatments. DAPI (blue), CD4 (green), CD8, and NK1.1 (red). Scale bars: 50 µm (*n* = 3 biological independent samples). f–q) Quantitative FCM analysis of the proportions of (f) M1‐TAMs, (g) M2‐TAMs, (h) M‐MDSCs, (i) PMN‐MDSCs, (j) Tregs, (k) Ki‐67^+^CD8^+^ T cells, (l) GZB^+^CD8^+^ T cells, (m) IFN‐γ^+^CD8^+^ T cells, (n) GZB^+^NK1.1^+^ cells, (o) IFN‐γ^+^NK1.1^+^ cells, (p) CD8^+^ T_CM_ (CD8^+^CD44^+^CD62L^+^) cells, and (q) CD8^+^ T_EM_ (CD8^+^CD44^+^CD62L^−^) in TDLNs from CMT167 tumor‐bearing mice on day 14 after different treatments (*n* = 5 biological independent samples). Results are presented as mean ± SD. Statistical significance was assessed using one‐way ANOVA with Tukey's post‐test. ns: no significant difference, *P < 0.05, **P < 0.01, ***P < 0.001, ****P < 0.0001.

TDLNs serve as the primary site for initiating and sustaining antitumor immunity.^[^ ^32^ ^]^ We then examined the infiltration of immune cells in TDLNs from CMT167 tumor‐bearing mice on day 14 post treatment (**Figure** **7**a). The infiltration of CD4^+^ T and CD8^+^ T cells in TDLNs was quantified using FCM, exhibiting a highest percentage of 32.0% and 57.22% for the “**GP@Gel Nap‐T**” group, respectively (Figure 7b,c). This increasing trend of T cell infiltration in TDLNs across different formulations aligned with enhanced antitumor efficacies. Furthermore, the proportion of CD3^−^NK1.1^+^ cells after **GP@Gel Nap‐T** treatment increased by 3.0‐fold, 2.8‐fold, 1.8‐fold, 2.2‐fold, 1.7‐fold, 1.2‐fold, and 1.5‐fold compared to “PBS”, “**Gel Nap‐T”**, “GDC0919”, “PTX”, “GDC0919&PTX**”**, “**G@Gel Nap‐T**”, and “**P@Gel Nap‐T”** groups, respectively (Figure 7d). We performed IF analysis to study the infiltration of CD4^+^ T, CD8^+^ T, and NK1.1^+^ cells in the TDLNs after different treatments. The “**GP@Gel Nap‐T**” group exhibited a mass of CD4^+^ T cells (green) infiltration in TDLNs, compared to the “PBS” group with a small amount of tumor‐infiltrating CD4^+^ T cells (Figure 7e; Figure S70a, Supporting Information). More importantly, **GP@Gel Nap‐T** treatment not only elevated the percentage of tumor‐infiltrating CD4^+^ T cells but also significantly increased the infiltration of CD8^+^ T and NK1.1^+^ cells in TDLNs, which was about 3.3‐fold and 5.5‐fold higher than other groups, respectively (Figure 7e; Figure S70b,c, Supporting Information). The populations of M1‐type and M2‐type TAMs in the TDLNs microenvironment from **GP@Gel Nap‐T**‐injected mice were 46.2% and 7.69%, respectively, as determined by FCM. The ratios of M2‐TAMs to M1‐TAMs in TDLNs of **GP@Gel Nap‐T**‐injected mice were 3.7‐fold higher than the “PBS” group (Figure 7f,g). These data confirmed that **GP@Gel Nap‐T**‐mediated cancer chemo‐immunotherapy significantly reduced immunosuppressive M2‐TAMs and increased inflammatory M1‐TAMs via the sustained release of PTX and GDC0919. Additionally, the levels of Ly6G and Ly6C in TDLNs MDSC cells were examined by FCM on day 14 post treatment. While there was no difference in M‐MDSC population among different groups (Figure 7h), the number of PMN‐MDSCs in Ly6G^+^Ly6C^−^ cells was attenuated by the free‐drug combination to 14.7%, further decreasing to 3.4% in the hydrogel system of the “**GP@Gel Nap‐T**” group (Figure 7i). This significant decrease was attributed to the codelivery of an IDO inhibitor (GDC0919) and a chemotherapeutic agent (PTX) via the peptide hydrogel. To further demonstrate that a mild thermal environment could amplify **GP@Gel Nap‐T**‐induced antitumor response, we also assessed the frequency of Tregs in the TDLNs (Figure 7j). The “**GP@Gel Nap‐T”** group exhibited the lowest Tregs frequency, consistent with the effective suppression against tumors in this group.

T cell proliferation was used to evaluate how **GP@Gel Nap‐T** influenced the immune responses of T cells. Ki‐67 staining was employed for the measurement of T cell proliferation (Figure 7k; Figure S71, Supporting Information). **GP@Gel Nap‐T** treatment resulted in a 2.7‐fold and 7.3‐fold increase in the absolute numbers of Ki‐67^+^CD4^+^ T cells and Ki‐67^+^CD8^+^ T cells in the TDLNs, respectively (Figure 7k; Figure S71, Supporting Information). Since T cell activation and proliferation are accompanied by enhanced cytotoxic function,^[^ ^33^ ^]^ TDLNs T cells on day 14 post treatment were first analyzed for cytotoxic effector molecule expression (GZB and IFN‐γ). In the TDLNs of **GP@Gel Nap‐T**‐treated mice, the frequency of T cells producing GZB and IFN‐γ was increased (Figure 7l,m; Figures S72 and S73, Supporting Information). We also observed that the frequency of activated NK cells producing GZB and IFN‐γ was significantly increased in the **GP@Gel Nap‐T**‐treated group (Figure 7n,o). These data supported that **GP@Gel Nap‐T** treatment might activate a strong antitumor immunity to kill cancer cells through a high level of GZB and IFN‐γ secreted by T cells or NK cells. Moreover, the population of CD44^+^CD62L^+^ in CD4^+^ T cells (13.7%) (Figure S74a, Supporting Information) and CD8^+^ T cells (25.1%) (Figure 7p; Figure S75, Supporting Information), CD44^+^CD62^−^ in CD4^+^ T cells (22.5%) (Figure S74b, Supporting Information) and CD8^+^ T cells (14.4%) (Figure 7q; Figure S75, Supporting Information). In summary, **GP@Gel Nap‐T** enhanced the infiltration of T cells and NK cells into the TDLNs, along with their secretion of effector molecules. It also facilitated the conversion of M2‐TAMs to M1‐TAMs, reduction of Tregs and PMN‐MDSCs, and even long‐term immune memory properties.

## 3 Conclusion

In this study, we engineered a smart peptide hydrogel platform **GP@Gel Nap‐T** by co‐assembling PTX and GDC0919 with a PKM2‐responsive peptide hydrogelator **Nap‐T**, aiming to enhance the immuno‐chemotherapy of NSCLC. We first demonstrated that **GP@Gel Nap‐T** formed a stable hydrogel with a β‐sheet‐like secondary structure through rheology and CD analyses. TEM images further revealed that the micromorphology of **GP@Gel Nap‐T** was entangled and dense nanofibers. In vitro experiments confirmed that, upon treatment with PKM2‐overexpressing NSCLC cell lysates, **GP@Gel Nap‐T** underwent a gel‐to‐solution transition with the release of PTX and GDC0919. Cellular uptake experiments confirmed the efficient internalization of the released drugs by NSCLC cells. Moreover, we demonstrated the high efficacy of **GP@Gel Nap‐T** in inhibiting NSCLC cell viability, migration and proliferation, promoting apoptosis, as well as inducing cell cycle arrest at G2. Importantly, we found that **GP@Gel Nap‐T**‐treated NSCLC cells exhibited high levels of ATP, HMGB1, and CRT, as well as increased ROS and ROS‐dependent mitochondrial dysfunction, which confirmed efficient ICD induction. Meanwhile, we confirmed that **GP@Gel Nap‐T** effectively potentiated immune responses by inhibiting IDO‐mediated Kyn production. In vivo experiments revealed that **GP@Gel Nap‐T** leaded to significantly reduced tumor size and growth rate, rendering a significantly higher tumor inhibition rate of 73.1% and survival rate of 40% than the other counterparts. Through H&E analysis, Ki‐67 staining, and TUNEL assay, we further found that **GP@Gel Nap‐T** treatment leaded to enhanced tumor cell apoptosis and thus decreased proliferation. Importantly, in a CMT167 tumor‐bearing mouse model, we found that **GP@Gel Nap‐T** promoted mitochondrial oxidative stress (i.e., increased ROS and mitochondrial superoxide, decreased MMP and GSH), DAMPs release (i.e., CRT, HMGB1, ATP, and HSP70), DC recruitment (i.e., CD45^+^CD11c^+^), and DC maturation (i.e., CD80^+^CD86^+^) within tumor tissues. Meanwhile, the “**GP@Gel Nap‐T**” group exhibited significantly lower Kyn/Trp ratios in tumor tissues and serum samples compared with the other groups. These results together confirmed enhanced ICD induction and IDO inhibition by **GP@Gel Nap‐T** through controlled and sustained drug release. We further took an insight into the immune response by **GP@Gel Nap‐T** in the CMT167 tumor‐bearing mouse model. It was found that **GP@Gel Nap‐T** significantly increased the infiltration of CD4^+^ T, CD8^+^ T, and NK cells into tumor tissues and TDLNs, thereby boosting the antitumor immune response. It modulated the polarization of TAMs toward an antitumorigenic M1 phenotype (i.e., CD86^+^CD206^−^) and reduced immunosuppressive cell populations such as PMN‐MDSCs (i.e. Ly6G^+^Ly6C^−^) and Tregs (i.e. CD4^+^Foxp3^+^). This collective modulation resulted in a substantial enhancement of the antitumor immune response. Furthermore, we found a significant increase in Ki‐67^+^CD4^+^ and Ki‐67^+^CD8^+^ T cells following **GP@Gel Nap‐T** treatment, indicating robust T cell activation and expansion. Moreover, the “**GP@Gel Nap‐T**” group exhibited enhanced cytotoxic and inflammatory immune response, which were evidenced by the highest percentages of GZB^+^ and IFN‐γ^+^ CD4^+^, CD8^+^, and NK1.1^+^ cells, as well as highest levels of TNF‐α and IFN‐γ, while the lowest level of the immunosuppressive cytokine IL‐10 in tumor tissues. Importantly, the “**GP@Gel Nap‐T**” group leaded to the highest percentages of T_CM_ (CD44^+^CD62L^+^) and T_EM_ (CD44^+^CD62L^−^) cells in CMT167 tumor tissues and TDLNs, suggesting that **GP@Gel Nap‐T** not only enhanced the immediate antitumor immune response but also provided long‐term protection against tumor burdens, metastasis, or relapse.

Taken together, our peptide hydrogel has shown unique merits in biomedical applications. First, it has high biosafety, because peptides possess inherently high biosafety and the peptide hydrogel is entirely prepared from peptides, which can be completely degraded in body. Second, compared with most other carrier materials, our peptide hydrogel is much easier to synthesize (thanks to the well‐established solid‐phase peptide synthesis technology) and prepare (merely through a heating–cooling process), thus showing an advantage in terms of manufacture. Third, our peptide hydrogel can achieve the precise release of drugs in tumor lesions, which relies on the disassembly of the peptide hydrogel specifically activated by the tumor biomarker PKM2. Moreover, this injected peptide hydrogel can well balance the issues of drug bioavailability and toxicity. Fourth, our peptide hydrogel can persistently release drugs in tumor lesions for long‐lasting therapeutic efficacy. In this study, we really found that this drug release pattern could achieve better therapeutic effects through continuous intervention on certain cellular factors and immune cells. Based on the above encouraging results, we anticipate our smart peptide hydrogel platform might be applied for treatment of NSCLC as well as other diseases in clinic in near future.

## 4 Experimental Section

### Materials

Chlorotrityl chloride resin, Fmoc‐protected amino acids, and O‐benzotriazole‐*N*,*N*,*N'*,*N'*‐tetramethyluronium‐hexafluorophosphate were acquired from GL Biochem Co., Ltd. (Shanghai). *N*,*N*‐diisopropylethylamine (DIPEA) and trifluoroacetic acid (TFA) were sourced from Sigma‐Aldrich (Shanghai, China). Assay kits for methyl thiazolyl tetrazolium (MTT) cytotoxicity, glutathione (GSH), adenosine 5'‐triphosphate (ATP), one‐step TUNEL apoptosis assay kit, anti‐mouse calreticulin (CRT) and anti‐mouse Ki‐67 primary antibodies, DNase I, recombinant murine interleukin‐4 (IL‐4), recombinant murine interferon‐gamma (IFN‐γ), RIPA lysis buffer, PI, and phenylmethylsulfonyl fluoride (PMSF) were all obtained from Beyotime (Shanghai, China). JC‐1 mitochondrial membrane potential assay kit, ROS assay kit, MitoSOX red mitochondrial superoxide indicator, RNase A, and collagenase IV were purchased from YEASEA (Shanghai, China). Goat anti‐rabbit IgG H&L (Alexa Fluor 647), goat anti‐rabbit IgG H&L (Alexa Fluor 488), and anti‐IDO primary antibody were sourced from Abcam (Shanghai, China). GDC0919 and Paclitaxel were procured from MedChemExpress (Shanghai, China) and Aladdin (Shanghai, China), respectively. Mouse 1× lymphocyte separation medium, the ELISA kits of IFN‐γ, TNF‐α, and IL‐10 were purchased from DAKEWE (Beijing, China). Ehrlich's reagent was obtained from Solarbio (Beijing, China). 4′,6‐diamidino‐2‐phenylindole (DAPI) staining solution was sourced from Fcmacs Biotech (Nanjing, China). Annexin V‐FITC/PI apoptosis detection kit was acquired from Vazyme (Nanjing, China). Zombie NIR fixable viability kit, anti‐CD16/32, anti‐CD45‐Brilliant Violet 421, anti‐CD11c‐APC, anti‐CD11b‐PE/Dazzle 594, anti‐F4/80‐Alexa Fluor 488, anti‐CD206‐PE, anti‐CD80‐Brilliant Violet 711, anti‐CD86‐PE/Cyanine7, anti‐Ly6G‐Brilliant Violet 510, anti‐Ly6C‐Brilliant Violet 605, anti‐CD3‐FITC, anti‐CD4‐PerCP/Cyanine5.5, anti‐CD8a‐APC, anti‐Foxp3‐PE, anti‐Ki‐67‐Brilliant Violet 605, anti‐NK1.1‐APC, anti‐Granzyme B‐PE, anti‐IFN‐γ‐APC, anti‐CD11c‐FITC, anti‐CD44‐Brilliant Violet 605, anti‐CD62L‐PE/Cyanine 7, purified anti‐CD8a and purified anti‐NK1.1 antibody, precision count beads, 7‐AAD viability staining solution, the buffer of true‐nuclear transcription factor, fixation, and intracellular staining permeabilization wash were all purchased from BioLegend Inc. (San Diego, USA). The primary antibodies for HSP70, the secondary antibodies for mouse and rabbit horseradish peroxidase (HRP) (IgG H&L), recombinant murine granulocyte‐macrophage colony‐stimulating factor (GM‐CSF), IL‐2, and IL‐15 were acquired from Proteintech (Wuhan, China). ELISA kits of high mobility group box 1 protein (HMGB1) and pyruvate kinase M2 (PKM2) were purchased from Elabscience (Wuhan, China). HMGB1 and CD4 antibody were obtained from AiFang biological (Hunan, China). Primary antibodies for Beclin‐1, LC3A/B, SQSTM1/p62, Cytochrome c, PARP, Bak, Bim, Bad, IDO, PKM2, Caspase‐3, Caspase‐7, Caspase‐9, Calreticulin, Phospho‐Akt, and GAPDH were all purchased from Cell Signaling Technology (Boston, USA). CellTrace CFSE cell proliferation kit and Dynabeads mouse T‐activator CD3/CD28 were sourced from Thermo Scientific (Waltham, MA). Roswell Park Memorial Institute (RPMI) 1640 medium, DMEM, fetal bovine serum (FBS), penicillin, and streptomycin were purchased from Gibco (Grand Island, NY, USA). PVDF transfer membranes (0.45 µm and 0.22 µm) were fabricated by Millipore (Bedford, MA, USA).

### Instruments

Millipore ultrapure water was acquired using a Milli‐Q purification system. HPLC purification procedures were conducted on a Shimazu UFLC system, featuring two LC‐20AP pumps and an SPD‐20A UV/vis detector, employing a Shimazu PRC‐ODS column. HPLC analysis was carried out utilizing an Agilent 1260 HPLC system, equipped with a G1322A pump and in‐line diode array UV detector, and utilizing an Agilent Zorbax 300SB‐C18 RP column with CH_3_CN (0.1% of TFA) and ultrapure water (0.1% of TFA) as the eluent. The molecular weights of **Nap‐T** and **Nap‐Tp** were assayed using MALDI‐TOF MS on an Ultraflextreme instrument. The critical aggregation concentration (CAC) was assessed utilizing a UV–vis spectrometer (UV‐2600i, Shimadzu, Japan). ^1^H NMR and ^13^C NMR spectra were recorded on a Bruker Avance III HD 600 MHz. Rheology tests were performed using a Haake RheoStress 6000 (Thermo Scientific, USA) with cone‐and‐plate geometry (1°/20 mm) at a gap of 370 µm. CD spectra were obtained with a Circular Dichroism instrument (Applied Photophysics, England). TEM investigations, utilized for observing morphology and measuring assembly size, were carried out on a JEOL JEM‐1400Flash. A microplate reader (Infinite F50; Tecan, Männedorf, Switzerland) was employed for measuring the optical density of MTT, LDH, and ELISA assays. Images were acquired using a Zeiss LSM 710 microscope through CLSM. Cell cycle arrest assessment and quantification as well as immune cell population sorting were conducted using FCM systems, including FACSCalibur and FACSymphony A5 SORP. The in vitro release of two drugs (PTX, GDC0919) and Kyn generation were assessed using an Agilent 1200 SL Series RRLC/6410B Triple Quad MS (Agilent, Santa Clara, CA, USA). In vivo fluorescence imaging of mice was conducted using an in vivo imaging system (IVIS Spectrum, USA). Paraffin slices were imaged with an inverted optical microscope (XD‐202, Jiangnan Yongxin, China). Images of tissue sections subject to immunofluorescence (IF) staining were captured by Pannoramic MIDI (3D HISTECH, Budapest, Hungary). The automatic biochemical assay system (model Chemray 800, manufactured by Rayto in Shenzhen, China) was utilized to assess liver and renal functions. Routine blood examination utilized the BC‐2800vet automated hematological analyzer, manufactured by Mindray in Shenzhen, China. WB images were captured using the Tanon 4600 automatic chemiluminescence image analysis system (Shanghai, China).

### Critical Aggregation Concentration (CAC) Measurement

A UV–vis spectrophotometer (UV‐2600i, Shimadzu) was employed to measure the transmittance of various concentrations of **Nap‐T** or **Nap‐Tp** (ranging from 2 mm to 31.3 µm) across wavelengths from 200 to 700 nm. The transmittances at 230 nm were plotted to determine the CACs of the peptides dissolved in PBS with a concentration of 0.01 m and a pH of 7.4.

### Dynamic Rheology

All 1.0 wt% hydrogels (**Gel Nap‐T** and **GP@Gel Nap‐T**) were swiftly applied onto the rheometer plate (Haake RheoStress 6000, Thermo Scientific), which had been preconditioned at 5 °C. Subsequently, the parallel plate tool was lowered to achieve a gap height of 0.5 mm, and the temperature was gradually increased to 37 °C to initiate gelation. A dynamic frequency sweep (0.1–100 rad s^−1^ at a constant 1.0% strain) ensued, followed by a dynamic strain sweep (0.1–1000% strain at a constant 6 rad s^−1^, frequency = 1 Hz) for all samples, ensuring that the hydrogels remained within the linear viscoelastic region.

### Circular Dichroism (CD) Measurement

Using a CD spectrometer (Applied Photophysics), all 1.0 wt% hydrogels (**Gel Nap‐T** and **GP@Gel Nap‐T**) were measured at 20 °C and elevated temperatures, spanning the wavelength range of 280−190 nm, with a path length of 1.0 mm, scan speed of 50 nm min^−1^, and a response time of 2.0 s.

### Cell Culture

Human lung cancer cell lines A549, NCIH1975, NCIH466, murine lung cancer cell lines CMT167 and Lewis, human breast cancer cell line BT549, human glioblastoma cell line U251, and murine glioblastoma cell line GL261 were all obtained from American Type Culture Collection (ATCC). NCM460 cells (human colonic epithelial), HT29 cells (human colorectal carcinoma), MDAMB231 cells (human breast cancer), and 4T1 cells (murine breast cancer) were kindly gifted by Prof. Wang at China Pharmaceutical University, Nanjing, China. Human lung epithelial cell line BEAS2B was obtained as a gift from Mr. Zhao at Southeast University, Nanjing, China. Human Burkitt's lymphoma cell line Raji and human leukemic cell line K562 were kindly supplied by Mr. Lin (Central South University, Changsha, China). The mouse TAP2 mutant cell line RMAS was kindly donated by Prof. Bi (Institute of Advanced Technology, Chinese Academy of Sciences, Shenzhen, China). A549, CMT167, Lewis, BEAS2B, MDAMB231, 4T1, and U251 cells were preserved in‐house and cultured in DMEM medium supplemented with 10% FBS, 100 U mL^−1^ of penicillin, and 100 µg mL^−1^ of streptomycin. NCIH1975, NCIH466, HT29, NCM460, BT549, Raji, K562, and RMAS were cultured in RPMI‐1640 medium enriched with 100 U mL^−1^ of both penicillin and streptomycin as antibiotics, along with 10% FBS, under conditions of 5% CO_2_ and 37 °C in a humidified environment. GL261 cells were cultured in DMEM/F12 supplemented with 10% FBS, 100 U mL^−1^ of penicillin, and 100 µg mL^−1^ of streptomycin. Splenic lymphocytes were isolated from C57BL/6 female mice using lymphocyte separation medium and density gradient centrifugation. Splenic lymphocytes were cultured for at least 3 days in RPMI‐1640 complete medium with 20 ng mL^−1^ recombinant murine IL‐2 and 20 ng mL^−1^ recombinant murine IL‐15 for natural killer (NK) cells activation and expansion before use. For T cells activation and expansion, splenic lymphocytes were cultured in RPMI‐1640 complete medium containing 20 ng mL^−1^ recombinant murine IL‐12, 1 µg mL^−1^ anti‐CD3/CD28 beads for at least 3 days before use. BMDCs were obtained from the femurs and tibiae of C57BL/6 female mice, and incubated with complete RPMI‐1640 medium supplemented with 20 ng mL^−1^ recombinant murine GM‐CSF and 10 ng mL^−1^ recombinant murine IL‐4 for at least 1 week.

### Cellular Uptake

A total of 1 × 10^5^ A549 and CMT167 cells were seeded onto a glass‐bottom dish and allowed to incubate overnight. Subsequently, the culture medium was replaced with **P@Gel Nap‐T** (PTX: 1 µg mL^−1^) in DMEM medium. Following incubation for 12 and 24 h, the treated cells underwent washing three times with PBS, fixation, and staining with DAPI (2 mg mL^−1^) at room temperature for 1 h. CLSM was employed to visualize the cells using a confocal microscope (Zeiss LSM 710), and quantification was performed using ImageJ (version 1.54).

### Cell Viability

Initially, the examination of **Gel Nap‐T**, GDC0919, PTX, GDC0919&PTX, **G@Gel Nap‐T**, **P@Gel Nap‐T**, and **GP@Gel Nap‐T** cytotoxicity involved treating A549 and CMT167 cells, which were plated in 96‐well microplates (2.5 × 10^4^ cells per well), with varying concentrations of the aforementioned compounds for 24, 48, and 72 h. Subsequently, MTT assay was employed to assess cell viability. Specifically, 10 µL of MTT solution (5 mg mL^−1^) was introduced into each well, followed by a 4 h incubation at 37 °C. Formed formazan crystals in each well were dissolved using 150 µL DMSO, and the resulting absorbance at 492 nm was measured using a microplate reader (Infinite F50, Tecan). Cell viability was determined by comparing the results with untreated cells.

### Cell Death Assessment

5 × 10^4^ A549 and CMT167 cells subjected to various treatments including PBS, **Gel Nap‐T** (**Nap‐T**: 520 µm), GDC0919 (80 ng mL^−1^), PTX (80 ng mL^−1^), GDC0919&PTX (80 ng mL^−1^), **G@Gel Nap‐T** (**Nap‐T**: 520 µm; GDC0919: 80 ng mL^−1^), **P@Gel Nap‐T** (**Nap‐T**: 520 µm; PTX: 80 ng mL^−1^), and **GP@Gel Nap‐T** (**Nap‐T**: 520 µm; GDC0919 and PTX: 80 ng mL^−1^). After 48 h of incubation, the cells were collected, washed three times with PBS, and stained with Annexin V‐FITC and PI, and subsequently analyzed using FCM (FACSymphony A5 SORP, BD Biosciences). The data analysis was performed utilizing FlowJo (version 10.0.8).

### Scratch Wound healing Assay

5 × 10^4^ A549 and CMT167 cells were seeded into a 6‐well culture dish and subsequently exposed to PBS, **Gel Nap‐T** (**Nap‐T**: 520 µm), GDC0919 (80 ng mL^−1^), PTX (80 ng mL^−1^), GDC0919&PTX (80 ng mL^−1^), **G@Gel Nap‐T** (**Nap‐T**: 520 µm; GDC0919: 80 ng mL^−1^), **P@Gel Nap‐T** (**Nap‐T**: 520 µm; PTX: 80 ng mL^−1^), and **GP@Gel Nap‐T** (**Nap‐T**: 520 µm; GDC0919 and PTX: 80 ng mL^−1^) for 0 h, 12 h, and 24 h. A wound was created in the center of each well using a 200 µL yellow pipette tip. Subsequently, the wells were gently rinsed with PBS to eliminate cellular debris. Following this, each well received incomplete culture medium, and images were captured at 0 h, 12 h, and 24 h (XD‐202, Jiangnan Yongxin). The wound healing ratio was quantified using ImageJ software (version 1.54).

### Transwell Migration Assays

To assess the inhibitory impact of **GP@Gel Nap‐T** on the in vitro migration of NSCLC cells, 2 × 10^4^ A549 and CMT167 cells, suspended in 100 µL serum‐free medium, were introduced into the upper chambers of transwell plates. Simultaneously, 600 µL of complete medium with 10% FBS was dispensed into the lower compartment. Various treatments were applied to both chambers, including PBS, **Gel Nap‐T** (**Nap‐T**: 520 µm), GDC0919 (80 ng mL^−1^), PTX (80 ng mL^−1^), GDC0919&PTX (80 ng mL^−1^), **G@Gel Nap‐T** (**Nap‐T**: 520 µm; GDC0919: 80 ng mL^−1^), **P@Gel Nap‐T** (**Nap‐T**: 520 µm; PTX: 80 ng mL^−1^), and **GP@Gel Nap‐T** (**Nap‐T**: 520 µm; GDC0919 and PTX: 80 ng mL^−1^). Following a 48 h incubation period, the chambers were gently washed twice from upper to lower with PBS, and the migrated cells in the lower chambers were fixed with 4% paraformaldehyde for 30 min. Subsequently, the chambers were stained with a 0.5% crystal violet solution for 30 min. The cells in the upper chamber were then carefully wiped off with a PBS‐soaked cotton swab. Migrated cells were observed under a microscop (XD‐202, Jiangnan Yongxin) and quantified using ImageJ software (version 1.54).

### Colony Formation Assay

To evaluate the inhibitory effects of **GP@Gel Nap‐T** on the colony‐forming abilities of A549 and CMT167 cells in vitro, 400 A549 and CMT167 cells were plated in 6‐well dishes and allowed to incubate overnight. Subsequently, the cells were treated with various conditions, including PBS, **Gel Nap‐T** (**Nap‐T**: 520 µm), GDC0919 (80 ng mL^−1^), PTX (80 ng mL^−1^), GDC0919&PTX (80 ng mL^−1^), **G@Gel Nap‐T** (**Nap‐T**: 520 µm; GDC0919: 80 ng mL^−1^), **P@Gel Nap‐T** (**Nap‐T**: 520 µm; PTX: 80 ng mL^−1^), and **GP@Gel Nap‐T** (**Nap‐T**: 520 µm; GDC0919 and PTX: 80 ng mL^−1^). After one weeks, visible colonies were observed, followed by washing with cold PBS and fixation with 4% paraformaldehyde for 30 min. Subsequently, the cells were stained with 0.5% crystal violet for 30 min, and images of the plates were captured using a camera. The number of colonies was quantified using ImageJ software (version 1.54).

### Cell Cycle Analysis

5 × 10^4^ A549 and CMT167 cells exposed to different conditions, including complete medium, **Gel Nap‐T** (**Nap‐T**: 520 µm), GDC0919 (80 ng mL^−1^), PTX (80 ng mL^−1^), GDC0919&PTX (80 ng mL^−1^), **G@Gel Nap‐T** (**Nap‐T**: 520 µm; GDC0919: 80 ng mL^−1^), **P@Gel Nap‐T** (**Nap‐T**: 520 µm; PTX: 80 ng mL^−1^), and **GP@Gel Nap‐T** (**Nap‐T**: 520 µm; GDC0919 and PTX: 80 ng mL^−1^) for 48 h were collected, followed by triple washing with PBS. After fixation with 70% v/v ice‐cold ethanol, the cells were stained with a solution of PI at a concentration of 50 µg mL^−1^, which also contained RNase A at a concentration of 100 µg mL^−1^ at 37 °C for 0.5 h under dark conditions. The obtained results were analyzed using a FCM (FACSCalibur, BD Biosciences) and interpreted using Modfit LT 5.0 software.

### Animal Model

C57BL/6 female mice aged 4–6 weeks were procured from Beijing Vital River Laboratory Animal Technology Co., Ltd (Beijing, China). These mice were kept in a controlled environment with a 12 h diurnal cycle, maintaining an ambient temperature of 24 °C, and a relative humidity level of 50%. The housing conditions adhered to barrier standards. The Nanjing Medical University's Animal Care and Use Committee (IACUC) granted approval for all animal experiments conducted under the project number IACUC‐2104050‐1, ensuring compliance with ethical standards and minimizing redundancy in reporting.

### WB Analysis

A549 and CMT167 cells were cultured in DMEM supplemented with various treatments including PBS, **Gel Nap‐T** (**Nap‐T**: 520 µm), GDC0919 (80 ng mL^−1^), PTX (80 ng mL^−1^), GDC0919&PTX (80 ng mL^−1^), **G@Gel Nap‐T** (**Nap‐T**: 520 µm; GDC0919: 80 ng mL^−1^), **P@Gel Nap‐T** (**Nap‐T**: 520 µm; PTX: 80 ng mL^−1^), and **GP@Gel Nap‐T** (**Nap‐T**: 520 µm; GDC0919 and PTX: 80 ng mL^−1^) for 48 h. Subsequently, cellular proteins were extracted and subjected to electrophoresis on SDS‐PAGE gels with a 15% acrylamide concentration 6.5–15%. Subsequently, the separated proteins were transferred onto PVDF membranes. The membranes were then probed with specific primary antibodies targeting Caspase 9, Caspase 3, Caspase 7, Cytochrome c, Bak, Bim, Bax, CRT HMGB1, HSP70, LC3A/B, p62/SQSTM1, Beclin‐1, p‐AKT, and GAPDH for a duration of ≈12–16 h at 4 °C. Afterward, the membranes underwent three washes with Tris‐buffered saline with Tween 20, followed by incubation with an HRP‐conjugated secondary antibody for a duration of 2 h at room temperature. GAPDH was used as an internal control to normalize the protein loading across different samples. Finally, the WB analysis was conducted using a chemiluminescence imaging system (Tanon 4600) and quantified using ImageJ software (version 1.54). To evaluate ICD signaling pathway protein expression in vivo, samples of tumor tissues were collected from CMT167 tumor‐bearing mice on day 14 post various treatments. Next, protein extraction from fresh cancer tissues followed a conventional method. Initially, 100 mg of fresh frozen tissues underwent homogenization using a syringe‐type, hand‐held homogenizer and were subsequently incubated in 1 mL of RIPA buffer containing PMSF at 0 °C. The resulting tissue lysates were centrifuged at a force of 12 000 × *g* for a duration of 15 min at a temperature of 4 °C, and the supernatants fractions were subsequently collected. Finally, protein electrophoresis and blot analysis were performed as described above. The blot signal analysis was conducted using a chemiluminescence imaging system (Tanon 4600) and quantified using ImageJ software (version 1.54).

### Intracellular ROS Assay

To evaluate intracellular ROS levels, the ROS assay kit DCFH‐DA was employed. Initially, A549 and CMT167 cells were categorized into eight groups: PBS, **Gel Nap‐T** (**Nap‐T**: 520 µm), GDC0919 (80 ng mL^−1^), PTX (80 ng mL^−1^), GDC0919&PTX (80 ng mL^−1^), **G@Gel Nap‐T** (**Nap‐T**: 520 µm; GDC0919: 80 ng mL^−1^), **P@Gel Nap‐T** (**Nap‐T**: 520 µm; PTX: 80 ng mL^−1^), and **GP@Gel Nap‐T** (**Nap‐T**: 520 µm; GDC0919 and PTX: 80 ng mL^−1^). After a 48 h incubation period, the cells underwent triple washing with PBS and subsequent incubation with fresh culture medium. Subsequently, the cells were exposed to 5 µm of DCFH‐DA for a duration of 30 min in the absence of light (at 37 °C in 5% CO_2_). Images were captured using a Zeiss LSM 710 confocal microscope and quantified using ImageJ software (version 1.54). Additionally, intracellular ROS levels were further assessed and quantified using FCM (FACSymphony A5 SORP, BD Biosciences). The FCM data analysis was performed utilizing FlowJo (version 10.0.8). For ex vivo ROS evaluation, the NSCLC model of mice was established. The ROS production efficacy of PBS, **Gel Nap‐T**, GDC0919 (5 mg kg^−1^), PTX (10 mg kg^−1^), GDC0919&PTX (GDC0919: 5 mg kg^−1^; PTX: 10 mg kg^−1^), **G@Gel Nap‐T** (GDC0919: 5 mg kg^−1^), **P@Gel Nap‐T** (PTX: 10 mg kg^−1^), and **GP@Gel Nap‐T** (GDC0919: 5 mg kg^−1^; PTX: 10 mg kg^−1^) was evaluated by 0.5 h after intravenous injection of DCFH‐DA (60 µg per mouse) on day 14. Mice were euthanized, and tumor tissue was collected, frozen, and sectioned. The fluorescence images were recorded by using Pannoramic MIDI (3D HISTECH). Additionally, the ex vivo ROS were further assessed and quantified using FCM. Specifically, tumor tissues were digested in a solution containing collagenase IV (1 mg mL^−1^) and DNase I (15 U mL^−1^) in PBS buffer at a temperature of 37 °C for a duration of 60 min to yield a cell suspension. Subsequently, the dissociated cells were passed through a 70 µm mesh cell filter and stained by DCFH‐DA (5 µm). Following this, the cells underwent triple washing with warm PBS, were collected, and quantified using FCM (FACSymphony A5 SORP, BD Biosciences). The FCM data analysis was performed utilizing FlowJo (version 10.0.8).

### MitoSOX Assay

A549 and CMT167 cells were treated with PBS, **Gel Nap‐T** (**Nap‐T**: 520 µm), GDC0919 (80 ng mL^−1^), PTX (80 ng mL^−1^), GDC0919&PTX (80 ng mL^−1^), **G@Gel Nap‐T** (**Nap‐T**: 520 µm; GDC0919: 80 ng mL^−1^), **P@Gel Nap‐T** (**Nap‐T**: 520 µm; **PTX**: 80 ng mL^−1^), and **GP@Gel Nap‐T** (**Nap‐T**: 520 µm; GDC0919 and PTX: 80 ng mL^−1^) for 48 h. Finally, the cells were exposed to a MitoSOX Red reagent working solution (5 µm) at a temperature of 37 °C for a duration of 30 min in the absence of light. Following this, the cells underwent triple washing with warm PBS, were collected, assessed and quantified using FCM (FACSymphony A5 SORP, BD Biosciences). For the in vivo examination of mitochondrial superoxide, samples of tumor tissues were collected from CMT167 tumor‐bearing mice on day 14 post various treatments. Tumor tissues were digested in a solution containing collagenase IV (1 mg mL^−1^) and DNase I (15 U mL^−1^) in PBS buffer at a temperature of 37 °C for a duration of 60 min to yield a cell suspension. Subsequently, the dissociated cells were passed through a 70 µm mesh cell filter and stained by MitoSOX Red (5 µm). Following this, the cells underwent triple washing with warm PBS, were collected, and quantified using FCM (FACSymphony A5 SORP, BD Biosciences). The FCM data analysis was performed utilizing FlowJo (version 10.0.8).

### Mitochondrial GSH Assay

For the evaluation of mitochondrial GSH levels in vitro, A549 and CMT167 cells underwent treatment with different formulations: PBS, **Gel Nap‐T** (**Nap‐T**: 520 µm), GDC0919 (80 ng mL^−1^), PTX (80 ng mL^−1^), GDC0919&PTX (80 ng mL^−1^), **G@Gel Nap‐T** (**Nap‐T**: 520 µm; GDC0919: 80 ng mL^−1^), **P@Gel Nap‐T** (**Nap‐T**: 520 µm; PTX: 80 ng mL^−1^), and **GP@Gel Nap‐T** (**Nap‐T**: 520 µm; GDC0919 and PTX: 80 ng mL^−1^). After a 48 h incubation, the mitochondria were isolated and lysed. The resulting lysates were collected via centrifugation, and the protein precipitation solution from the GSH assay kit was added and thoroughly mixed. Following rapid freezing‐thawing, the samples were incubated on ice for 5 min and then subjected to centrifugation. The resulting supernatant was used to determine the mitochondrial GSH levels following the protocol provided with the GSH assay kit. The measurement of GSH levels in the mitochondria was conducted using a microplate reader (Infinite 200 Pro, Tecan). For the in vivo examination of GSH, samples of tumor tissues were collected from CMT167 tumor‐bearing mice on day 14 post various treatments. Tumor tissues were digested in a solution containing collagenase IV (1 mg mL^−1^) and DNase I (15 U mL^−1^) in PBS buffer at a temperature of 37 °C for a duration of 60 min to yield a cell suspension. Subsequently, the dissociated cells were passed through a 70 µm nylon mesh cell filter and isolated, lysed mitochondria. The collected lysates were obtained through centrifugation, and the protein precipitation solution from the GSH assay kit was added and mixed thoroughly. After rapid freezing‐thawing, the samples were incubated on ice for 5 min and subsequently subjected to centrifugation. The resulting supernatant was utilized to assess the mitochondrial GSH levels following the protocol outlined in the GSH assay kit. The measurement of GSH levels in the mitochondria was performed using a microplate reader (Infinite 200 Pro, Tecan).

### MMP Assay

To quantify the MMP changes of A549 and CMT167 cells after different treatments in vitro, JC‐1 probe was used. Specifically, cells were plated at a density of 500 000 cells per well in 6‐well culture dishes, and then treated with PBS, **Gel Nap‐T** (**Nap‐T**: 520 µm), GDC0919 (80 ng mL^−1^), PTX (80 ng mL^−1^), GDC0919&PTX (80 ng mL^−1^), **G@Gel Nap‐T** (**Nap‐T**: 520 µm; GDC0919: 80 ng mL^−1^), **P@Gel Nap‐T** (**Nap‐T**: 520 µm; PTX: 80 ng mL^−1^), and **GP@Gel Nap‐T** (**Nap‐T**: 520 µm; GDC0919 and PTX: 80 ng mL^−1^) for a duration of 48 h. Subsequently, the treated cells were stained by JC‐1 probe. Following this, the cells underwent triple washing with warm PBS, were collected, assessed and quantified using FCM (FACSymphony A5 SORP, BD Biosciences). For the in vivo examination of MMP, samples of tumor tissues were collected from CMT167 tumor‐bearing mice on day 14 post various treatments. Tumor tissues were digested in a solution containing collagenase IV (1 mg mL^−1^) and DNase I (15 U mL^−1^) in PBS buffer at a temperature of 37 °C for a duration of 60 min to yield a cell suspension. Subsequently, the dissociated cells were passed through a 70 µm nylon mesh cell filter and stained by JC‐1 probe. Following this, the cells underwent triple washing with warm PBS, were collected, and quantified using FCM (FACSymphony A5 SORP, BD Biosciences). The FCM data analysis was performed utilizing FlowJo (version 10.0.8).

### IDO Inhibition Evaluation

To assess the IDO inhibitory potential of **GP@Gel Nap‐T** in vitro, A549 and CMT167 cells were initially stimulated by IFN‐γ (50 ng mL^−1^) in a 96‐well microplate at a concentration of 5 × 10^3^ cells per well. Afterward, they were incubated with various compounds for 48 h. Following the incubation period, 150 µL of supernatant from each well was transferred to a fresh 96‐well plate. To ensure complete hydrolysis of formed N‐formylkynurenine to Kyn, 75 µL of 30% trichloroacetic acid was added and maintained at 50 °C for an additional 6 h. Afterward, the supernatants were transferred to another new 96‐well plate and mixed with an equal volume of Ehrlich reagent a duration of 10 min at ambient temperature before measuring their absorbance at 492 nm utilizing a microplate spectrophotometer (Infinite F50, Tecan). For the in vivo examination of the Kyn to Trp ratios, samples of tumor tissues and serum were collected from CMT167 tumor‐bearing mice on day 14 post various treatments. Tumor tissues were blended in water to create a homogeneous mixture, and this mixture was then combined with acetonitrile (i.e. 1:1, v/v), centrifuged to obtain cell supernatant. Subsequently, the cell supernatant and serum were treated with 10 µL of 30% trichloroacetic acid each, followed by centrifugation at 12 000 × *g* for 10 min to eliminate proteins. The resulting supernatant was filtered through a 0.22 µm PTFE filtration membrane and subjected to analysis via HPLC (Agilent 6410B). Trp and Kyn levels were monitored at 280 nm and 360 nm, respectively, using HPLC conducted with a flow rate of 1.0 mL min^−1^ and a mobile phase composed of acetonitrile and water (15/85, v/v). Elution times for Trp and Kyn were 5.2 min and 8.9 min, respectively, with concentrations determined using established calibration curves.

### Evaluation of Immunogenic Cell Death Markers and DCs Maturation

To assess the presence and abundance of CRT on the surface of tumor cell, FCM was performed. A549 and CMT167 cells were exposed to each formulation for 12 h. Subsequently, CRT expression was identified using primary antibodies specific to CRT for 30 min, and then the cells were incubated for an additional 30 min with a secondary antibody labeled with Alexa Fluor 488 to visualize the CRT. Following this, the cells underwent triple washing with warm PBS, were collected, the percentage of CRT^+^ cells was assessed and quantified through FCM (FACSymphony A5 SORP, BD Biosciences). The FCM data analysis was performed utilizing FlowJo (version 10.0.8). To detect the level of HMGB1 released into the culture medium, the supernatants of treated A549 and CMT167 cells after exposed to each formulation for 12 h. After that, supernatants were harvested and tested for HMGB1 levels using an ELISA kit according to the manufacturer's instructions. Additionally, the amount of ATP released into the extracellular environment was measured using an ATP assay kit following the manufacturer's protocol. The extracellular secretion levels of ATP were quantified using an ATP assay kit following the manufacturer's protocol. For the in vitro investigation of DCs maturation, BMDCs were isolated from the femurs and tibiae of 4‐week‐old female C57BL/6J mice. Following the removal of red blood cells, the cells were then cultured in complete RPMI‐1640 medium supplemented with recombinant murine GM‐CSF (20 ng mL^−1^) and recombinant murine IL‐4 (10 ng mL^−1^). Nonadherent cells were harvested for further tests were performed on day 7. CMT167 cells were pretreated with each formulation for 12 h. Then, BMDCs (5 × 10^5^ cells per well) and pretreated CMT167 cells (5 × 10^4^ per well) were cocultured for 24 h. DCs maturation were determined using FCM (FACSymphony A5 SORP, BD Biosciences) after staining with anti‐CD11c‐APC (dilution: 1:100), anti‐CD80‐Brilliant Violet 711, and anti‐CD86‐PE/Cyanine7 antibodies. The FCM data analysis was performed utilizing FlowJo (version 10.0.8). To detect DAMPs that are present in or associated with dead tumor cells in vivo, tumor tissues from CMT167 tumor‐bearing mice were collected on day 14 after different treatments. The tissues underwent IF with CRT, HMGB1, and anti‐CD11c‐APC antibodies as described above for IF analysis. The intensity of the fluorescence was measured using ImageJ software (version 1.54). DCs maturation in vivo was further determined using FCM (FACSymphony A5 SORP, BD Biosciences) after staining with anti‐CD11c‐APC, anti‐CD80‐Brilliant Violet 711, and anti‐CD86‐PE/Cyanine7 antibodies. The FCM data analysis was performed utilizing FlowJo (version 10.0.8).

### T and NK cell Proliferation

To investigate the potential of **GP@Gel Nap‐T** on T and NK cell proliferation, a coculture system involving lymphocytes and CMT167 cells was established. CMT167 cells were subjected to overnight stimulation with recombinant murine IFN‐γ (50 ng mL^−1^) to induce IDO expression. Then, **GP@Gel Nap‐T** or the other treatments were added to IFN‐γ stimulated CMT167 cells for 12 h. Splenic lymphocytes were isolated from C57BL/6 mice, and following the lysis of red blood cells. Following this step, the splenic lymphocytes were separated using lymphocyte separation medium and density gradient centrifugation. For T cell proliferation, splenic lymphocyte was activated using anti‐CD3/CD28 beads (1.0 µg mL^−1^) and incubated with IL‐2 (20 ng mL^−1^) before use. In a 24‐well plate, prestimulated CMT167 cells (5 × 10^5^ cells per well) were combined with activated splenic lymphocytes (1 × 10^6^ cells per well). After a 2 day co‐culture, splenic lymphocytes were collected and stained with Live/Dead‐APC/Cyanine7, anti‐CD45‐Brilliant Violet 421, anti‐CD3‐FITC, anti‐CD4‐PerCP/Cyanine5.5, and anti‐CD8a‐APC. Next, the cells were fixed, permeabilized, and then stained with anti‐Ki‐67‐Brilliant Violet 605 as per the manufacturer's instructions. The percentage of Ki‐67^+^ T cells was quantified using FCM (FACSymphony A5 SORP, BD Biosciences). The FCM data analysis was performed utilizing FlowJo (version 10.0.8). For NK cell proliferation assays, splenic lymphocytes were activated using recombinant murine IL‐15 (20 ng mL^−1^) and recombinant murine IL‐2 (20 ng mL^−1^) before use. Afterward, activated lymphocytes (1 × 10^6^ cells per well) were stained with 12 µm CFSE and mixed with pre‐stimulated CMT167 cells (5 × 10^5^ cells per well) in a 24‐well plate. Following a 2‐day coculture, splenic lymphocytes were collected and stained with Live/Dead‐APC/Cyanine7, anti‐CD45‐Brilliant Violet 421, anti‐CD3‐FITC, and anti‐NK1.1‐APC. The percentage of CFSE^+^ NK cells was quantified using FCM (FACSymphony A5 SORP, BD Biosciences). The FCM data analysis was performed utilizing FlowJo (version 10.0.8).

### T and NK Cell Cytotoxicity

To investigate the potential of **GP@Gel Nap‐T** on T and NK cell cytotoxicity, a co‐culture system involving lymphocytes and CMT167 cells was established. CMT167 cells were subjected to overnight stimulation with recombinant murine IFN‐γ (50 ng mL^−1^) to induce IDO expression. Afterward, CMT167 cells were treated with different formulations for 12 h and then stained with 2 µm CFSE. Splenic Lymphocytes were isolated from C57BL/6 mice, and following the lysis of red blood cells. Following this step, the splenic lymphocytes were separated using lymphocyte separation medium and density gradient centrifugation. For T cell cytotoxicity, lymphocyte was activated using anti‐CD3/CD28 beads (1.0 µg mL^−1^) and incubated with recombinant murine IL‐2 (20 ng mL^−1^) before use. In a 24‐well plate, pre‐stimulated and CFSE labeled CMT167 cells (0.1 × 10^5^ cells per well) were combined with activated splenic lymphocytes (1 × 10^6^ cells per well). After a 4 h co‐culture, cells were collected and stained with 7‐AAD, anti‐CD45‐Brilliant Violet 421, anti‐CD3‐FITC, and anti‐CD8a‐APC. The percentage of 7‐AAD^+^CFSE^+^ cells was quantified using FCM (FACSymphony A5 SORP, BD Biosciences). The FCM data analysis was performed utilizing FlowJo (version 10.0.8). For NK cell cytotoxicity assays, splenic lymphocytes were activated using recombinant murine IL‐15 (20 ng mL^−1^) and incubated with recombinant murine IL‐2 (20 ng mL^−1^) before use. Activated splenic lymphocytes (1 × 10^6^ cells per well) were mixed with pre‐stimulated CMT167 cells (5 × 10^5^ cells per well) in a 24‐well plate. Following a 4 h co‐culture, splenic lymphocytes were collected and stained with 7‐AAD, anti‐CD45‐Brilliant Violet 421, anti‐CD3‐FITC, and anti‐NK1.1‐APC. The percentage of 7‐AAD^+^CFSE^+^ cells was quantified using FCM (FACSymphony A5 SORP, BD Biosciences). The FCM data analysis was performed utilizing FlowJo (version 10.0.8).

### In Vivo Therapeutic Efficacy Assessment of GP@Gel Nap‐T

To explore the potential of **GP@Gel Nap‐T** in suppressing tumor growth through the induction of ICD and inhibition of IDO, female C57BL/6 mice aged 4–6 weeks were subcutaneously inoculated with CMT167 tumor cells (1 × 10^6^ cells per mouse). Upon reaching a tumor volume of 50 mm^3^, mice were randomly assigned into groups (*n* = 5) and received treatments via intratumoral administration. The treatment groups included PBS, **Gel Nap‐T**, GDC0919 (5 mg kg^−1^), PTX (10 mg kg^−1^), GDC0919&PTX (GDC0919: 5 mg kg^−1^; PTX: 10 mg kg^−1^), **G@Gel Nap‐T** (GDC0919: 5 mg kg^−1^), **P@Gel Nap‐T** (PTX: 10 mg kg^−1^), and **GP@Gel Nap‐T** (GDC0919: 5 mg kg^−1^; PTX: 10 mg kg^−1^). Tumor volume and body weight were assessed every other day throughout the experiment. Tumor volume was calculated using the formula *V* = (*L* × *W* ^2^)/2, where *L* and *W* represent the long and short axes of the tumor, respectively. Additionally, the body weight of each mouse was recorded using a digital balance. At the conclusion of the experiment, tumors were harvested and weighed.

### Tissue H&E Staining and TUNEL Assay

Tumor tissues were harvested and immersed in 4% paraformaldehyde for a day to prepare them for H&E staining and TUNEL assay. Subsequently, they were dehydrated in a series of sucrose PBS solutions with concentrations of 15% and 30% for 24 h each. Following this, the tumor tissues were immersed in a compound designed for optimal cutting temperature and kept at −80 °C for a duration of 2 h. Tissue sections were then cryosectioned, and the slices were subjected to staining with both H&E and the TUNEL kit, following the manufacturer's instructions. The slices were further counter‐stained with DAPI. During apoptosis, endonucleases become activated and cut the genomic DNA at specific points between nucleosomes, creating exposed 3′‐OH groups that can be tagged by terminal deoxynucleotidyl transferase using fluorescein‐labeled deoxyuridine triphosphate (FITC‐labeled dUTP). The stained tumor sections were visualized using a digital pathology system (Pannoramic MIDI, 3D HISTECH). The pathological signal was quantified using ImageJ software (version 1.54).

### Immunohistochemistry and Immunofluorescence Staining

To detect Ki‐67 in tumor tissue, IHC staining was performed. IF staining was used to determine the presence of CD4, CD8, and NK1.1 in tumor tissues and TDLNs. Initially, paraffin sections were heated at 60 °C for 1 h before undergoing dewaxing and rehydration. Antigen retrieval was then conducted in citric acid buffer (pH 6.0) for 20 min. Following this, the IHC sections were treated with 1% hydrogen peroxide to remove endogenous peroxidase activity. Then, for 1 h, the IHC slides were exposed to a solution containing 10% normal goat serum at room temperature to block nonspecific antibody binding. The sections were subsequently incubated overnight at 4 °C with primary antibodies against Ki‐67, CD4, CD8, and NK1.1. After incubation with Alexa Fluor 488‐conjugated secondary antibody or Alexa Fluor 647‐conjugated secondary antibody, the slides were mounted, and staining images were captured using a fluorescence microscope (Pannoramic MIDI, 3D HISTECH). The intensity of the fluorescence was measured using ImageJ software (version 1.54).

### Quantification of Tumor‐infiltrating Immune Cells

The composition of immune cell populations within tumor tissues and TDLNs post various treatments was assessed using FCM (FACSymphony A5 SORP, BD Biosciences). The mice from each treatment group were euthanized 14 days after they were inoculated. Tumor tissues and TDLNs were perfused, and collected, and then digested in a solution containing 1 mg mL^−1^ collagenase IV and 15 U mL^−1^ DNase I in PBS buffer at a temperature of 37 °C for 60 min to yield a cell suspension. Subsequently, the dissociated cells were passed through a 70 µm nylon mesh cell filter and centrifuged on Percoll gradients. To minimize nonspecific antibody binding, the cells were initially incubated with CD16/CD32 for 15 min to block Fc receptors. Various antibodies and fluorophores were employed to identify and characterize immune cell populations in the tumor tissues and TDLNs. These included Live/Dead‐APC/Cyanine7, anti‐CD45‐Brilliant Violet 421, anti‐CD11c‐APC, anti‐CD11b‐PE/Dazzle 594, anti‐F4/80‐Alexa Fluor 488, anti‐CD206‐PE, anti‐CD80‐Brilliant Violet 711, anti‐CD86‐PE/Cyanine7, anti‐Ly6G‐Brilliant Violet 510, anti‐Ly6C‐Brilliant Violet 605, anti‐CD3‐FITC, anti‐CD4‐PerCP/Cyanine5.5, anti‐CD8a‐APC, anti‐Foxp3‐PE, anti‐Ki‐67‐Brilliant Violet 605, anti‐NK1.1‐APC, anti‐Granzyme B‐PE, and anti‐IFN‐γ‐APC. For cytokine staining, cells were restimulated for 4 h and intracellular staining was performed following the manufacturer's instructions. Finally, FCM analysis (FACSymphony A5 SORP, BD Biosciences) was conducted on the cells. To further evaluate cytokine levels in vivo, mice were anesthetized, and tumor tissues were collected on day 14 after treatment. Tumor tissues were weighed, homogenized, and centrifuged to assay the levels of TNF‐α, IFN‐γ, and IL‐10 using ELISA kits as per the manufacturer's instructions. Gating scheme for FCM analysis for tumors and TDLNs was shown in Figure S76 of the Supporting Information.

### Biocompatibility Evaluation

The primary organs comprising the heart, liver, spleen, lungs, and kidneys, along with the skin, were gathered and preserved in 4% paraformaldehyde. Subsequently, these organs and the skin underwent paraffin embedding, were sectioned into 4 µm slices, and subjected to HE staining to assess histopathological alterations following distinct treatments. Blood samples were procured from each group for both biochemical analysis and routine blood examination. Liver and renal functions were assessed using an automated biochemical analyzer (Chemray 800, Rayto). Routine blood examination was conducted using the Mindray's BC‐2800vet, an automated system for hematology analysis.

### Statistical Analysis

The statistical analysis involved the presentation of all data as mean ± standard deviation (SD). One‐way analysis of variance (ANOVA) was employed for the statistical assessment of the data. GraphPad Prism 8 was utilized for all statistical analysis. The legend of each figure indicates the precise sample size utilized for the respective experiment. The significance thresholds were established as follows: a single asterisk (*) denoting *P*‐values less than 0.05, double asterisks (**) for *P*‐values less than 0.01, triple asterisks (***) for *P*‐values less than 0.001, and four asterisks (****) for *P*‐values less than 0.0001.

## Conflict of Interest

The authors declare no conflict of interest.

## Author Contributions

H.W., X.S., K.L., J.L., and H.J. contributed equally to this work. H.W., X.S., K.L., J.L., H.J., X.C., X.L., G.L., and H.X. conceived the project and designed the experiments. H.W. and X.S. synthesized and characterized the compounds and hydrogel. D.Y., Y.L., Y.D., and Y.L. contributed to the cell culture, cell viability, cell apoptosis, wound‐healing, transwell invasion, and colony formation assay. H.W. and J.L. performed the ROS, MitoSOX, GSH, MMP, IDO inhibition, ICD, T and NK cell proliferation, and T and NK cell cytotoxicity experiments. H.W., J.L., and H.J. contributed to the in vivo tumorigenesis and antitumor activity experiments. Y.L., Y.D., and Y.L. contributed to the WB. H.W., K.L., and J.L. contributed to the ELISA, H&E staining, IF staining, IHC, TUNEL assay, and FCM assays. X.Z. helped with the biocompatibility evaluation. H.W., X.S., K.L., J.L., H.J., X.C., X.L., G.L., and H.X. analyzed and interpreted the data, wrote and revised the manuscript. The final manuscript was approved by all the co‐authors.

## Supporting information



Supporting Information

## Data Availability

The data that support the findings of this study are available from the corresponding author upon reasonable request.

## References

[1] C. Gridelli , A. Rossi , D. P. Carbone , J. Guarize , N. Karachaliou , T. Mok , F. Petrella , L. Spaggiari , R. Rosell , Nat. Rev. Dis. Primers 2015, 1, 15009. 27188576 10.1038/nrdp.2015.9

[2] F. A. Greco , Lung Cancer 2001, 34, 53. 11742703 10.1016/s0169-5002(01)00393-2

[3] C. M. Scribano , J. Wan , K. Esbona , J. B. Tucker , A. Lasek , A. S. Zhou , L. M. Zasadil , R. Molini , J. Fitzgerald , A. M. Lager , J. J. Laffin , K. Correia‐Staudt , K. B. Wisinski , A. J. Tevaarwerk , R. O'Regan , S. M. McGregor , A. M. Fowler , R. J. Chappell , T. S. Bugni , M. E. Burkard , B. A. Weaver , Sci. Transl. Med. 2021, 13, 4811. 10.1126/scitranslmed.abd4811 PMC8612166 34516829

[4] a) T. S. Lau , L. K. Y. Chan , G. C. W. Man , C. H. Wong , J. H. S. Lee , S. F. Yim , T. H. Cheung , I. A. McNeish , J. Kwong , Cancer Immunol. Res. 2020, 8, 1099; 32354736 10.1158/2326-6066.CIR-19-0616

[5] a) S. Nagata , R. Hanayama , K. Kawane , Cell 2010, 140, 619; 20211132 10.1016/j.cell.2010.02.014

[6] a) D. L. Tang , R. Kang , C. B. Coyne , H. J. Zeh , M. T. Lotze , Immunol. Rev. 2012, 249, 158; 22889221 10.1111/j.1600-065X.2012.01146.x PMC3662247

[7] a) D. Hanahan , R. A. Weinberg , Cell 2011, 144, 646; 21376230 10.1016/j.cell.2011.02.013

[8] a) C. Uyttenhove , L. Pilotte , I. Théate , V. Stroobant , D. Colau , N. Parmentier , T. Boon , B. J. Van den Eynde , Nat. Med. 2003, 9, 1269; 14502282 10.1038/nm934

[9] a) F. Fallarino , U. Grohmann , K. W. Hwang , C. Orabona , C. Vacca , R. Bianchi , M. L. Belladonna , M. C. Fioretti , M. L. Alegre , P. Puccetti , Nat. Immunol. 2003, 4, 1206; 14578884 10.1038/ni1003

[10] a) D. D. Wang , X. B. Feng , L. Lu , J. E. Konkel , H. Y. Zhang , Z. Y. Chen , X. Li , X. Gao , L. W. Lu , S. T. Shi , W. J. Chen , L. Y. Sun , Arthritis Rheumatol. 2014, 66, 2234; 24756936 10.1002/art.38674 PMC4309486

[11] a) Y. C. Chen , R. Xia , Y. X. Huang , W. C. Zhao , J. Li , X. L. Zhang , P. C. Wang , R. Venkataramanan , J. Fan , W. Xie , X. C. Ma , B. F. Lu , S. Li , Nat. Commun. 2016, 7, 13443; 27819653 10.1038/ncomms13443 PMC5103075

[12] S. Sindhwani , A. M. Syed , J. Ngai , B. R. Kingston , L. Maiorino , J. Rothschild , P. MacMillan , Y. W. Zhang , N. U. Rajesh , T. Hoang , J. L. Y. Wu , S. Wilhelm , A. Zilman , S. Gadde , A. Sulaiman , B. Ouyang , Z. Lin , L. S. Wang , M. Egeblad , W. C. W. Chan , Nat. Mater. 2020, 19, 566. 31932672 10.1038/s41563-019-0566-2

[13] X. D. Liu , N. Shin , H. K. Koblish , G. J. Yang , Q. Wang , K. Wang , L. Leffet , M. J. Hansbury , B. Thomas , M. Rupar , P. Waeltz , K. J. Bowman , P. Polam , R. B. Sparks , E. W. Yue , Y. L. Li , R. Wynn , J. S. Fridman , T. C. Burn , A. P. Combs , R. C. Newton , P. A. Scherle , Blood 2010, 115, 3520. 20197554 10.1182/blood-2009-09-246124

[14] a) G. A. Orr , P. Verdier‐Pinard , H. McDaid , S. B. Horwitz , Oncogene 2003, 22, 7280; 14576838 10.1038/sj.onc.1206934 PMC4039039

[15] a) Y. Hua , H. Yin , X. Y. Liu , J. B. Xie , W. J. Zhan , G. L. Liang , Y. Shen , Adv. Sci. 2022, 9, 2202260; 10.1002/advs.202202260 PMC9353504 35618488

[16] a) F. Gao , X. H. Yang , W. L. Song , Small Methods 2024, 8, 2300753; 10.1002/smtd.202300753 37599261

[17] a) M. C. Branco , J. P. Schneider , Acta Biomater. 2009, 5, 817; 19010748 10.1016/j.actbio.2008.09.018 PMC2729065

[18] a) Y. F. Liu , Y. L. Yang , C. Wang , X. J. Zhao , Nanoscale 2013, 5, 6413; 23739953 10.1039/c3nr00225j

[19] W. W. Yang , Y. Xia , D. Hawke , X. J. Li , J. Liang , D. M. Xing , K. Aldape , T. Hunter , W. K. A. Yung , Z. M. Lu , Cell 2012, 150, 685. 22901803 10.1016/j.cell.2012.07.018 PMC3431020

[20] a) M. C. Hsu , W. C. Hung , Mol. Cancer 2018, 17, 35; 29455645 10.1186/s12943-018-0791-3 PMC5817853

[21] Z. M. Yang , G. L. Liang , L. Wang , B. Xu , J. Am. Chem. Soc. 2006, 128, 3038. 16506785 10.1021/ja057412y

[22] X. S. Yan , K. Wu , Y. Yuan , Y. Zhan , J. H. Wang , Z. Li , Y. B. Jiang , Chem. Commun. 2013, 49, 8943. 10.1039/c3cc44336a 23964363

[23] a) B. R. Wang , J. B. Han , Y. Jiang , S. Xu , R. Yang , Y. G. Kong , Z. Z. Tao , Q. Q. Hua , Y. Zou , S. M. Chen , Autophagy 2024, 20, 329; 37776538 10.1080/15548627.2023.2258052 PMC10813569

[24] M. A. Feitelson , A. Arzumanyan , R. J. Kulathinal , S. W. Blain , R. F. Holcombe , J. Mahajna , M. Marino , M. L. Martinez‐Chantar , R. Nawroth , I. Sanchez‐Garcia , D. Sharma , N. K. Saxena , N. Singh , P. J. Vlachostergios , S. C. Guo , K. Honoki , H. Fujii , A. G. Georgakilas , A. Bilsland , A. Amedei , E. Niccolai , A. Amin , S. S. Ashraf , C. S. Boosani , G. Guha , M. R. Ciriolo , K. Aquilano , S. Chen , S. I. Mohammed , A. S. Azmi , et al., Semin. Cancer Biol 2015, 35, S25. 25892662 10.1016/j.semcancer.2015.02.006 PMC4898971

[25] K. L. King , J. A. Cidlowski , Annu. Rev. Physiol. 1998, 60, 601. 9558478 10.1146/annurev.physiol.60.1.601

[26] J. A. Shen , Q. Yin , L. L. Chen , Z. W. Zhang , Y. P. Li , Biomaterials 2012, 33, 8613. 22910221 10.1016/j.biomaterials.2012.08.007

[27] Q. M. Yang , G. Shi , X. L. Chen , Y. Lin , L. Cheng , Q. Y. Jiang , X. Yan , M. Jiang , Y. M. Li , H. T. Zhang , H. L. Wang , Y. Wang , Q. N. Wang , Y. J. Zhang , Y. Liu , X. L. Su , L. Dai , M. H. Tang , J. Li , L. Zhang , Z. Y. Qian , D. C. Yu , H. X. Deng , Theranostics 2020, 10, 8382. 32724476 10.7150/thno.45391 PMC7381738

[28] a) J. Alexandre , Y. M. Hu , W. Q. Lu , H. Pelicano , P. Huang , Cancer Res. 2007, 67, 3512; 17440056 10.1158/0008-5472.CAN-06-3914

[29] a) K. Tu , H. Deng , L. Kong , Y. Wang , T. Yang , Q. Hu , M. Hu , C. L. Yang , Z. P. Zhang , ACS Appl. Mater. Interfaces 2020, 12, 16018; 32192326 10.1021/acsami.9b23084

[30] U. S. Gautam , T. W. Foreman , A. N. Bucsan , A. V. Veatch , X. Alvarez , T. Adekambi , N. A. Golden , K. M. Gentry , L. A. Doyle‐Meyers , K. E. Russell‐Lodrigue , P. J. Didier , J. L. Blanchard , K. G. Kousoulas , A. A. Lackner , D. Kalman , J. Rengarajan , S. A. Khader , D. Kaushal , S. Mehra , Proc. Natl. Acad. Sci. USA 2018, 115, E62. 29255022 10.1073/pnas.1711373114 PMC5776797

[31] Z. X. Li , Q. Pei , M. Zhao , Z. G. Xie , M. Zheng , Adv. Funct. Mater. 2024, 34, 2312500.

[32] M. I. Koukourakis , A. Giatromanolaki , Biochim. Biophys. Acta, Rev. Cancer 2022, 1877, 188704. 35227831 10.1016/j.bbcan.2022.188704

[33] M. R. Verneris , M. Karami , J. Baker , A. Jayaswal , R. S. Negrin , Blood 2004, 103, 3065. 15070686 10.1182/blood-2003-06-2125

