# Supplementary material for: Pyruvate Kinase M2‐Responsive Release of Paclitaxel and Indoleamine 2,3‐Dioxygenase Inhibitor for Immuno‐Chemotherapy of Nonsmall Cell Lung Cancer
Source: Adv Sci (Weinh). 2024 Dec 24;12(7):2409790. doi: 10.1002/advs.202409790 (PMC11831488; doi:10.1002/advs.202409790)
Supplement: Supplementary file 1 — Supporting Information [file ADVS-12-2409790-s001.pdf]

ADVANCED SCIENCE
Open Access

Supporting Information

for *Adv. Sci.*, DOI 10.1002/advs.202409790

Pyruvate Kinase M2-Responsive Release of Paclitaxel and Indoleamine 2,3-Dioxygenase Inhibitor for Immuno-Chemotherapy of Nonsmall Cell Lung Cancer

Haisi Wu, Xianbao Sun, Kaiming Li, Jinyu Li, Hui Jiang, Dan Yan, Ya Lin, Yan Ding, Yawen Lu, Xiaole Zhu, Xufeng Chen, Xiaolin Li*, Gaolin Liang* and Huae Xu**

Supporting Information

Pyruvate Kinase M2-Responsive Release of Paclitaxel and Indoleamine 2,3-Dioxygenase Inhibitor for Immuno-Chemotherapy of Non-Small Cell Lung Cancer

Haisi Wu, Xianbao Sun, Kaiming Li, Jinyu Li, Hui Jiang, Dan Yan, Ya Lin, Yan Ding, Yawen Lu, Xiaole Zhu, Xufeng Chen, Xiaolin Li*, Gaolin Liang*, and Huae Xu**

Dr. H.Wu, Prof. H. Xu
Department of Pharmaceutics, School of Pharmacy, Nanjing Medical University; Nanjing, 211116, China
The Affiliated Suzhou Hospital of Nanjing Medical University, Suzhou Municipal Hospital, Gusu School, Nanjing Medical University; Suzhou, 215002, China
E-mail: xuhuae@njmu.edu.cn (H.X.)

Dr. X. Sun, Prof. G. Liang
State Key Laboratory of Digital Medical Engineering, School of Biological Science and Medical Engineering, Southeast University, Nanjing 211189, China
E-mail: gliang@seu.edu.cn (G.L.)

Dr. K. Li, J. Li, H. Jiang, D. Yan, Y. Lin, Y. Ding, Y. Lu
Department of Pharmaceutics, School of Pharmacy, Nanjing Medical University; Nanjing, 211116, China

X. Zhu, Prof. X. Chen
Department of Emergency Medicine, The First Affiliated Hospital of Nanjing Medical University, Nanjing 210029, China
E-mail: cxfyx@njmu.edu.cn (X.C.)

Prof. X. Li
Department of Geriatric Gastroenterology, the First Affiliated Hospital of Nanjing Medical University, Nanjing 210009, China
E-mail: lxl@njmu.edu.cn (X.L.)

This file includes:

1. Syntheses and Characterizations (**Schemes S1-S2**)

2. Supporting Figures **(Figures S1-S76)**

3. Supporting Table **(Table S1)**

1. Syntheses and Characterizations

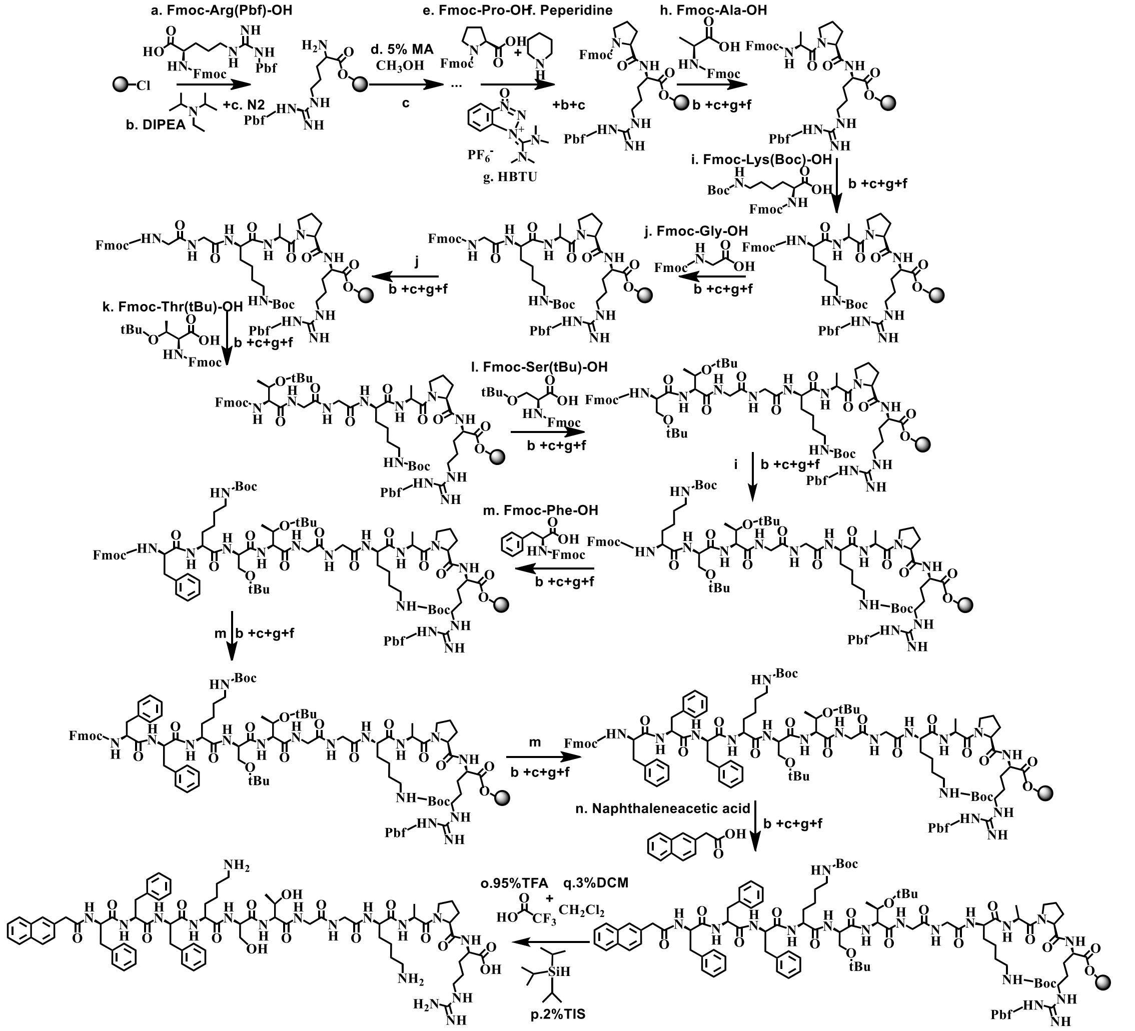

Scheme S1. The synthetic route for **Nap-T**.

Synthesis of Nap-T: Nap-Phe-Phe-Phe-Lys-Ser-Thr-Gly-Gly-Lys-Ala-Pro-Arg-OH (**Nap-T**) was synthesized with solid phase peptide synthesis (SPPS).[1] Scheme S1 show the synthetic routes for **Nap-T**. The compounds **Nap-T** was purified with HPLC. The molecular weight of **Nap-T** was determined through matrix-assisted laser desorption ionization time-of-flight (MALDI-TOF) mass spectrometry (Ultraflextreme). MALDI-TOF mass spectrum of **Nap-T** is shown in Figure S1. MS of **Nap-T**: calcd. $[M + H]^+$: 1510.78, $[M + Na]^+$: 1532.78; found ESI-MS (*m*/*z*) $[M + H]^+$: 1510.86, (*m*/*z*) $[M + Na]^+$: 1532.84. ^{1}H NMR and ^{13}C NMR spectra were recorded on a Bruker Avance III HD 600 MHz. ^{1}H NMR spectrum of **Nap-T** is shown in Figure S2. ^{1}H NMR (600 MHz, DMSO-d_6) δ 8.28 (d, J = 8.6 Hz, 1 H), 8.21 (d, J = 7.9 Hz, 1 H), 8.18-8.08 (m, 5 H), 8.04 (d, J = 8.3 Hz, 1 H), 7.98-7.92 (m, 1 H), 7.86-7.83 (m, 1 H), 7.81-7.71 (m, 8 H), 7.57 (s, 1 H), 7.50-7.42 (m, 3 H), 7.28-7.23 (m, 5 H), 7.19-7.11 (m, 14 H), 4.62-4.57 (m, 1H), 4.55-4.33 (m, 7 H), 4.30-4.21 (m, 2 H), 4.17-4.06 (m, 2 H), 3.82-3.70 (m, 4 H), 3.70-3.66

(m, 1 H), 3.63-3.52 (m, 4 H), 3.48-3.45 (m, 1 H), 3.15-3.08 (m, 2 H), 3.08-2.96 (m, 2 H), 2.93-2.88 (m, 1 H), 2.87-2.81 (m, 1 H), 2.79-2.72 (m, 5 H), 2.71-2.65 (m, 1 H), 2.05-1.99 (m, 1 H), 1.93-1.80 (m, 3 H), 1.80-1.58 (m, 5 H), 1.57-1.46 (m, 9 H), 1.38-1.27 (m, 4 H), 1.20 (d, J = 6.8 Hz, 3 H), 1.05 (d, J = 6.3 Hz, 3 H). ^{13}C NMR spectrum of **Nap-T** is shown in Figure S3. ^{13}C NMR (151 MHz, DMSO-d_6) δ 173.42, 171.56, 171.52, 171.44, 171.03, 170.84, 170.60, 170.58, 170.41, 170.33, 169.81, 169.18, 168.59, 156.83, 137.79, 137.63, 137.58, 133.90, 132.92, 131.73, 129.23, 128.08, 128.01, 127.92, 127.58, 127.45, 127.37, 127.24, 126.28, 126.22, 126.14, 125.98, 125.46, 66.45, 61.57, 59.11, 58.28, 54.83, 53.72, 52.19, 51.88, 51.57, 46.69, 46.32, 42.19, 41.93, 40.32, 40.06, 38.68, 37.48, 37.42, 31.64, 31.40, 28.95, 28.29, 26.65, 26.53, 25.12, 24.42, 22.19, 22.16, 19.63, 16.81.

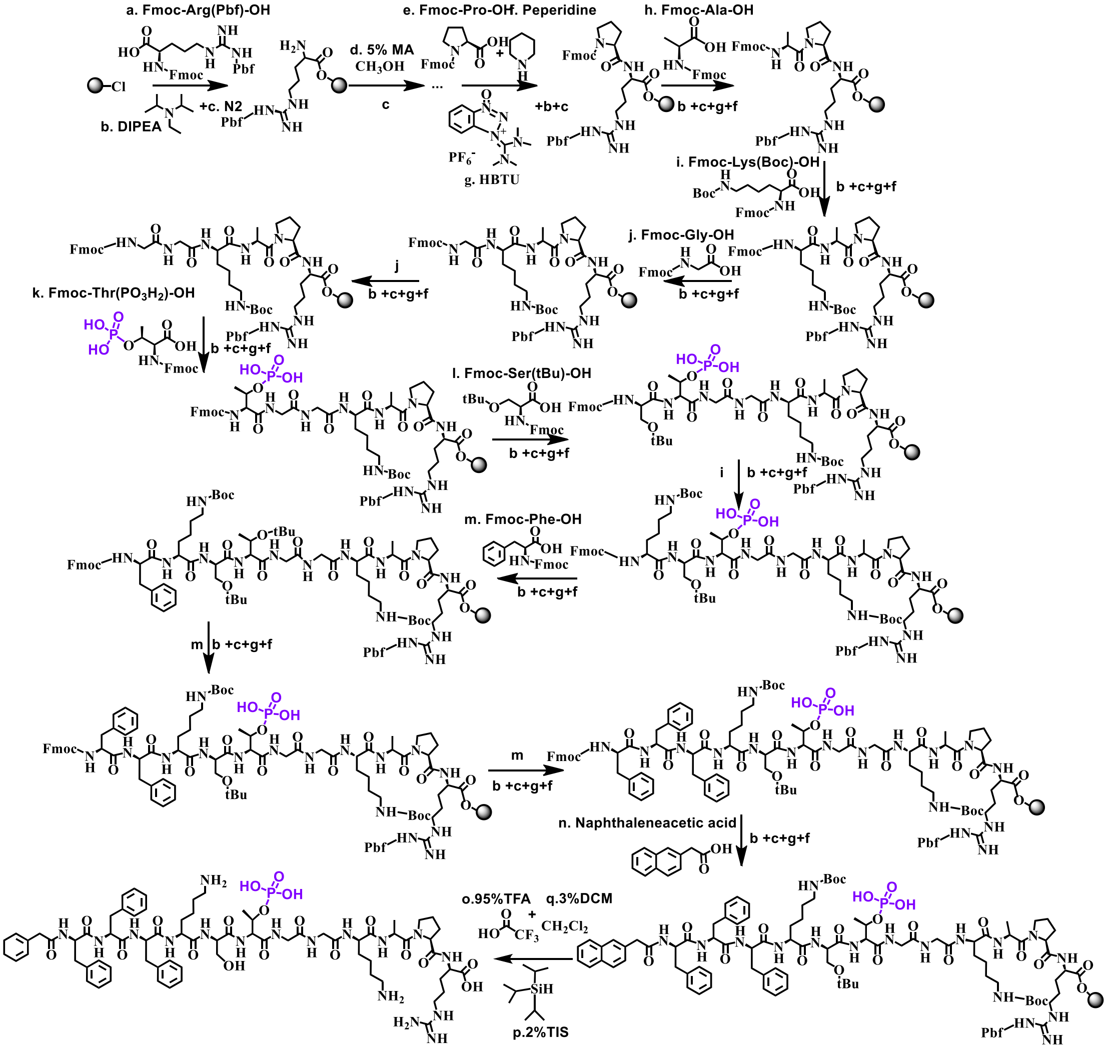

Scheme S2. The synthetic route for **Nap-Tp**.

Synthesis of Nap-Tp: Nap-Phe-Phe-Phe-Lys-Ser-Thr(H_2PO_3)-Gly-Gly-Lys-Ala-Pro-Arg-OH (**Nap-Tp**) was synthesized with solid phase peptide synthesis (SPPS).[1] Scheme S2 show the synthetic routes for **Nap-Tp**. The compounds **Nap-Tp** were purified with HPLC. The molecular

weight of **Nap-Tp** was determined through matrix-assisted laser desorption ionization time-of-flight (MALDI-TOF) mass spectrometry (Ultraflextreme). MALDI-TOF mass spectrum of **Nap-Tp** is shown in Figure S4. MS of **Nap-Tp**: calcd. $[M + H]^+$: 1590.74; found ESI-MS (*m*/*z*) $[M + H]^+$: 1590.83. ^1H NMR spectrum of **Nap-Tp** is shown in Figure S5. ^1H NMR (600 MHz, DMSO-d_6) δ 8.42-8.35 (m, 1 H), 8.28 (d, *J* = 8.5 Hz, 1 H), 8.22 (d, *J* = 7.4 Hz, 3 H), 8.16-8.07 (m, 3 H), 7.93 (d, *J* = 8.2 Hz, 1 H), 7.87-7.68 (m, 13 H), 7.57 (s, 1 H), 7.50-7.44 (m, 3 H), 7.28-7.24 (m, 5 H), 7.19-7.11 (m, 15 H), 4.71-4.65 (m, 1 H), 4.65-4.59 (m, 1 H), 4.54-4.46 (m, 4 H), 4.45-4.39 (m, 3 H), 4.38-4.34 (m, 1 H), 4.26-4.21 (m, 1 H), 4.19-4.15 (m, 1 H), 3.87-3.36 (m, 10 H), 3.14-3.04 (m, 4 H), 3.02-2.97 (m, 1 H), 2.93-2.89 (m, 1 H), 2.88-2.83 (m, 1 H), 2.80-2.73 (m, 6 H), 2.71-2.66 (m, 1 H), 2.08-2.00 (m, 1 H), 1.94-1.69 (m, 6 H), 1.68-1.63 (m, 1 H), 1.62-1.50 (m, 10 H), 1.41-1.26 (m, 5 H), 1.23-1.18 (m, 6 H). ^{13}C NMR spectrum of **Nap-Tp** is shown in Figure S6. ^{13}C NMR (151 MHz, DMSO-d_6) δ 173.36, 171.74, 171.57, 171.16, 170.99, 170.88, 170.82, 170.64, 170.36, 169.81, 169.42, 169.00, 168.52, 156.82, 137.78, 137.65, 137.57, 133.89, 132.91, 131.72, 129.27, 129.23, 128.07, 128.00, 127.92, 127.58, 127.45, 127.36, 127.23, 126.28, 126.22, 126.14, 125.99, 125.47, 69.81, 62.90, 61.43, 59.13, 57.19, 55.14, 53.69, 52.21, 52.05, 51.41, 46.72, 46.34, 42.19, 40.31, 40.06, 38.57, 37.48, 37.41, 31.59, 31.20, 28.97, 28.21, 26.58, 26.51, 25.16, 24.44, 22.19, 21.93, 18.40, 16.74.

2. Supporting Figures

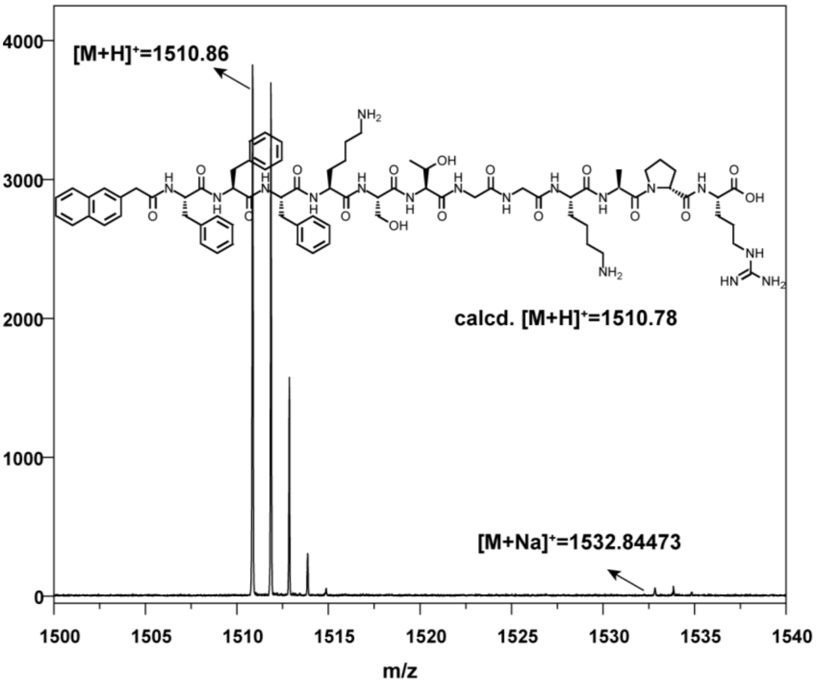

Figure S1. MALDI-TOF mass spectrum of **Nap-T**.

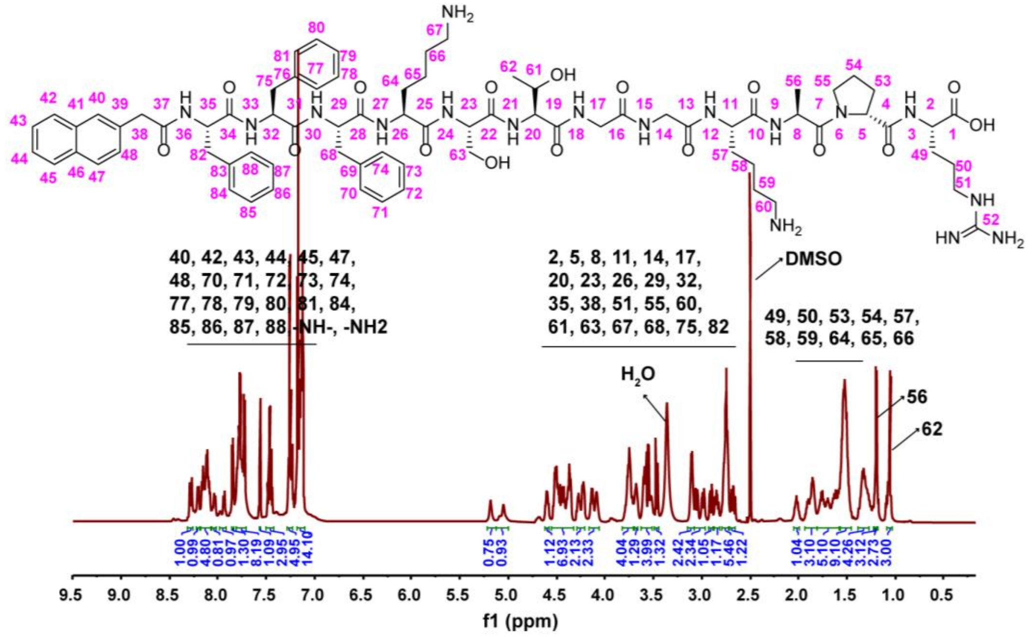

Figure S2. ^{1}H NMR spectrum of **Nap-T** in DMSO-d_6.

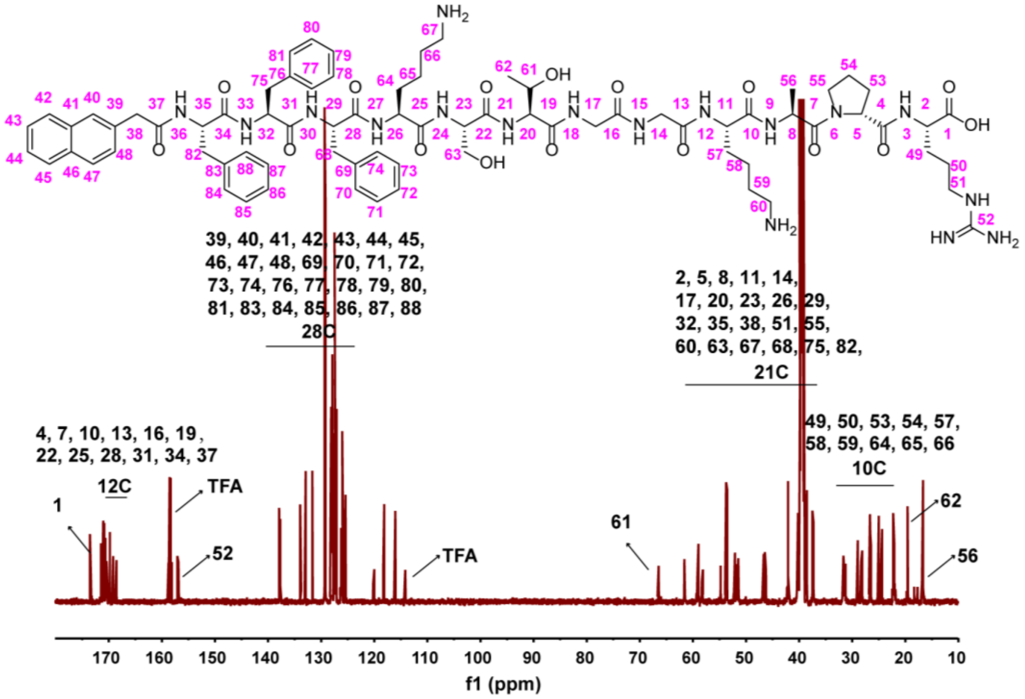

Figure S3. ^{13}C NMR spectrum of **Nap-T** in DMSO-d_6.

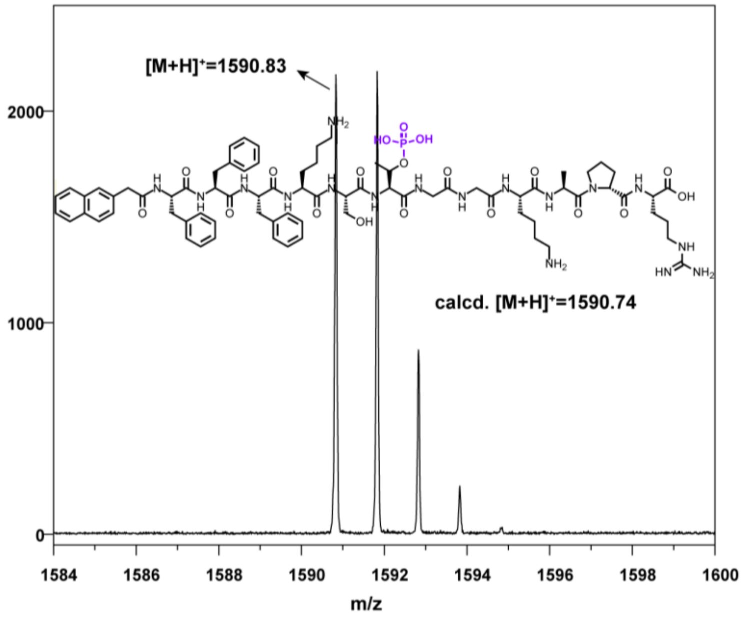

Figure S4. MALDI-TOF mass spectrum of **Nap-Tp**.

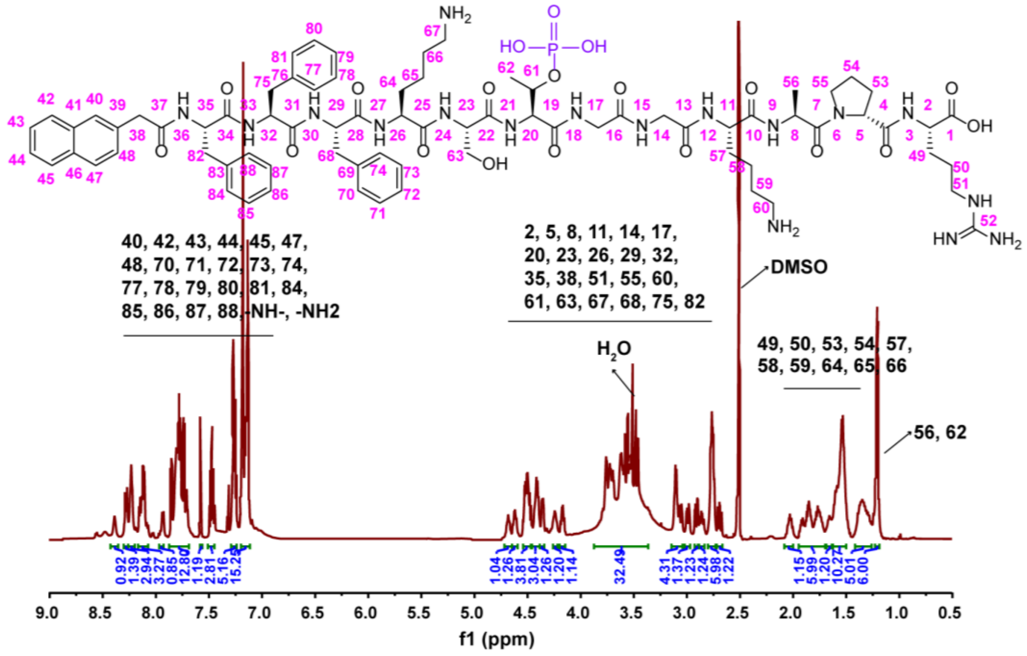

Figure S5. ^{1}H NMR spectrum of **Nap-Tp** in DMSO-d_6.

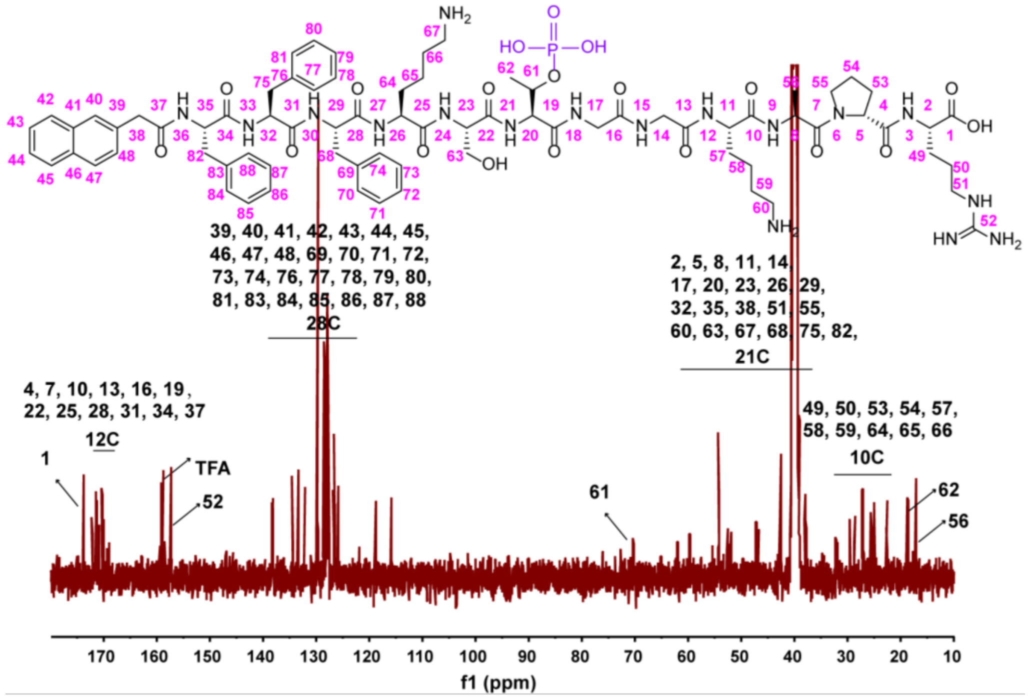

Figure S6. ^{13}C NMR spectrum of **Nap-Tp** in DMSO-d_6.

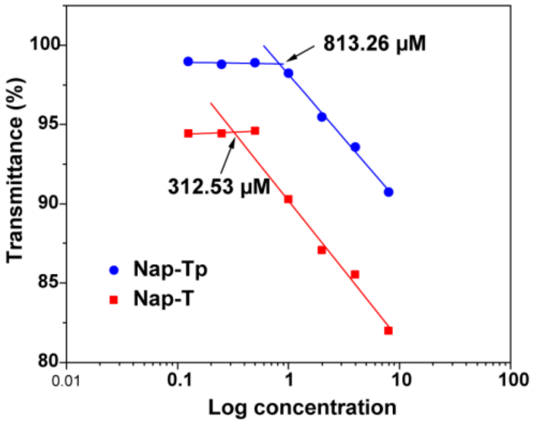

Figure S7. CACs results for pure compounds **Nap-T** and **Nap-Tp**.

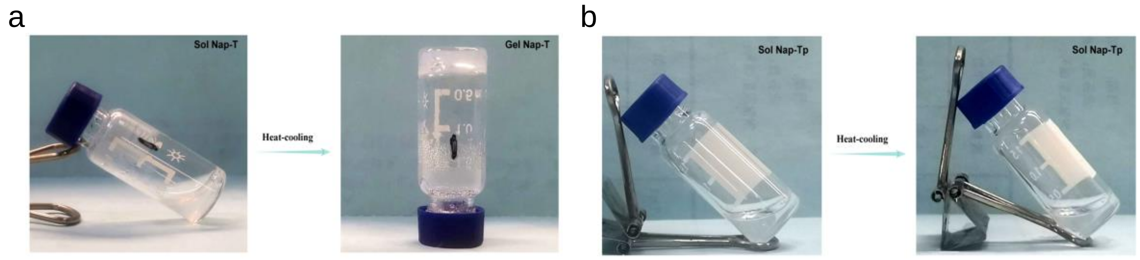

Figure S8. Optical images of 1.0 wt% **Nap-T** (a) and **Nap-T** (b) before and after heat-cooling process.

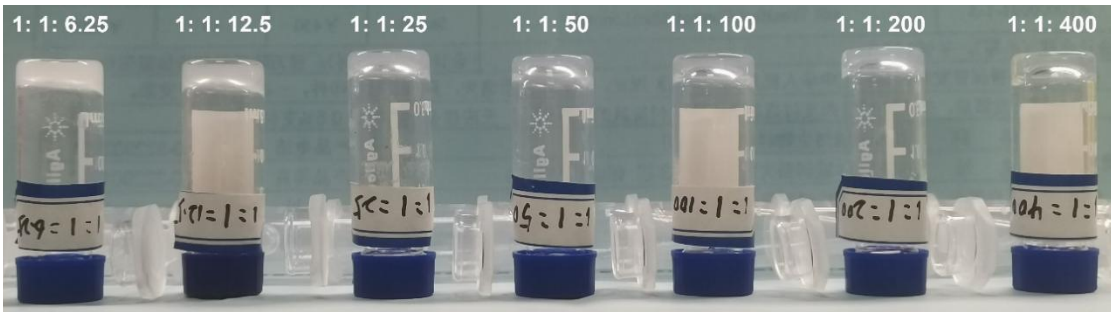

Figure S9. Photographs of 1.0 wt% **GP@Gel Nap-T** mixtures with different GDC0919/PTX/**Nap-T** molar ratios (1: 1: 6.25, 1: 1: 12.5, 1: 1: 25, 1: 1: 50, 1: 1: 100, 1: 1: 200, and 1: 1: 400) after the heating-cooling process.

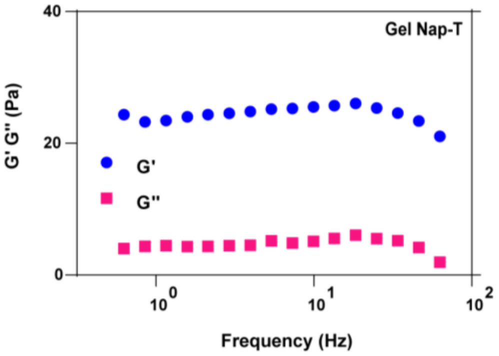

Figure S10. Frequency dependence of the dynamic storage moduli (G′) and the loss moduli (G′′) of 1.0 wt% **Gel Nap-T** (25 °C, strain: 1.0%).

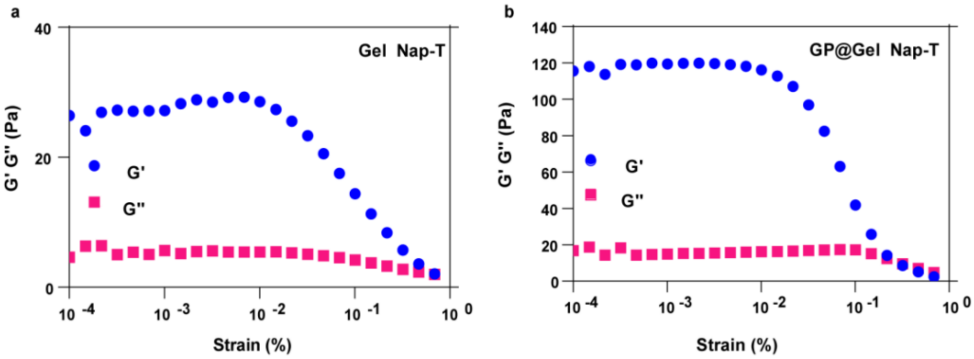

Figure S11. Strain dependence of the dynamic storage moduli (G´) and the loss moduli (G´´) of 1.0 wt% (a) **Gel Nap-T** and (b) **GP@Gel Nap-T** (25 ℃, frequency: 1 Hz).

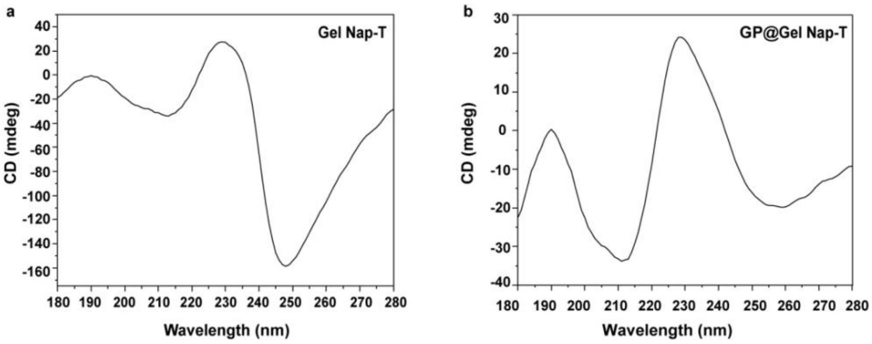

Figure S12. CD spectra of (a) **Gel Nap-T** and (b) **GP@Gel Nap-T** at 1.0 wt% in PBS (0.01 M, pH 7.4).

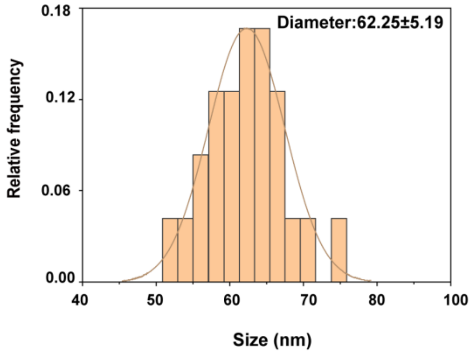

Figure S13. Statistics of diameter distribution of nanofibers in Figure 1c.

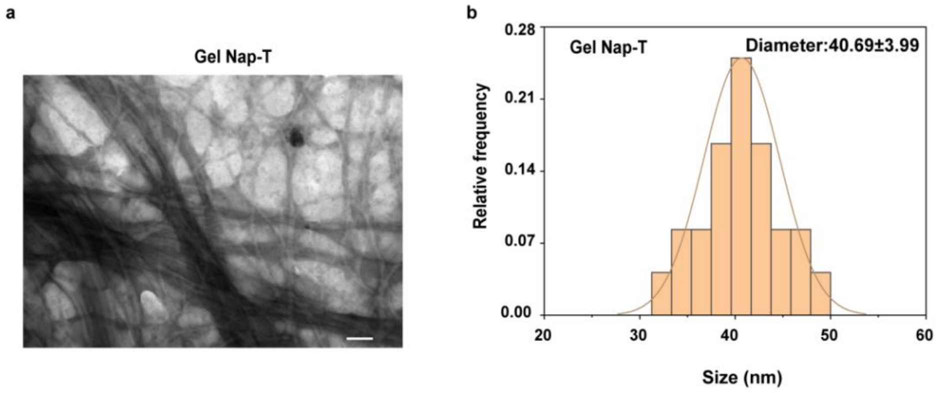

Figure S14. (a) TEM images and (b) corresponding statistics of diameter distribution of 1.0 wt% **Gel Nap-T.** Scale bars, 0.5 µm.

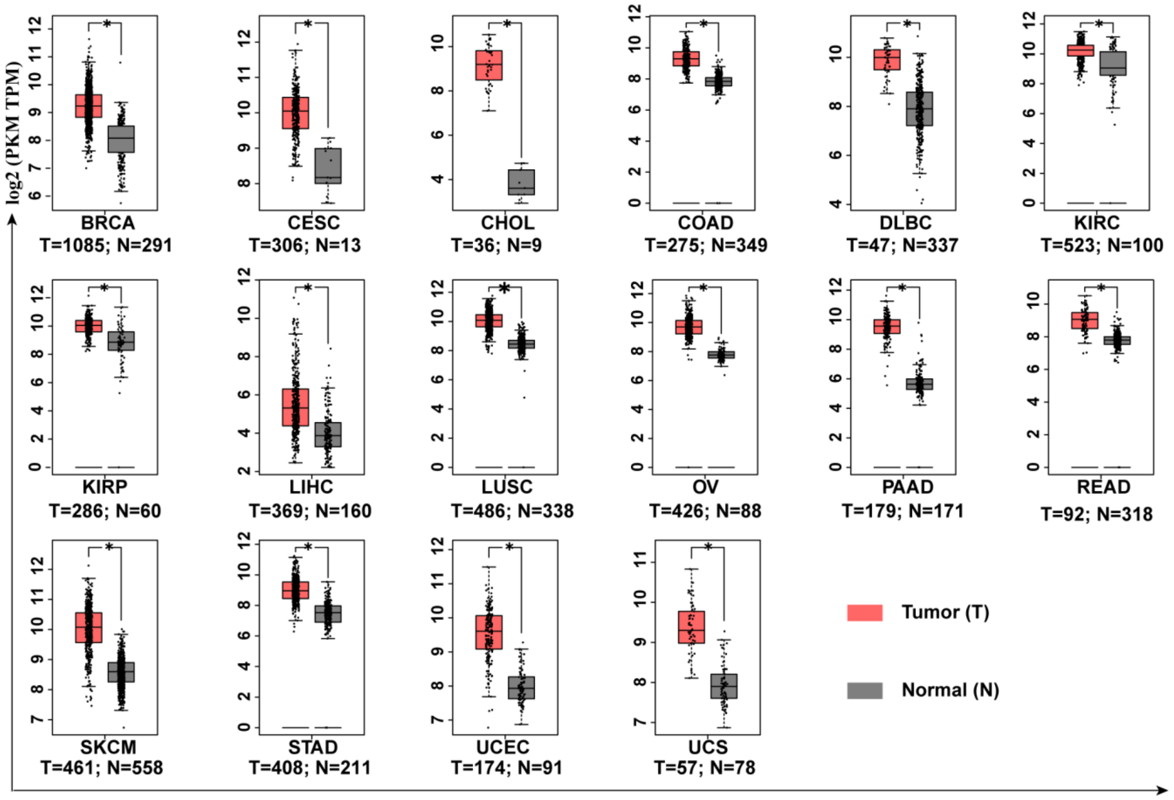

Figure S15. Expression of PKM2 in BRCA, CESC, CHOL, COAD, DLBC, KIRC, KIRP, LIHC, LUSC, OV, PAAD, READ, SKCM, STAD, UCEC and UCS or the corresponding normal tissues from the TCGA database, represented as transcripts per million (TPM). *P < 0.05.

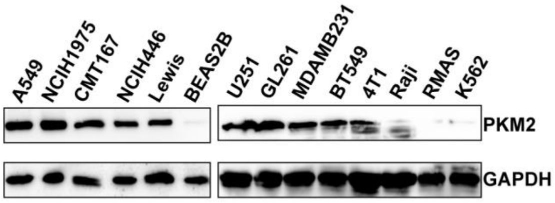

Figure S16. WB results of the expression levels of PKM2 in different cancer cells. Control, GAPDH.

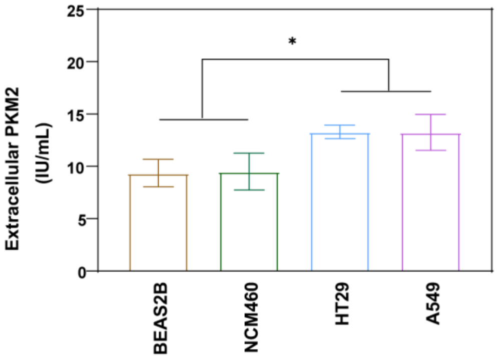

Figure S17. ELISA quantitative analysis of PKM2 levels in the conditioned media (n = 3, biological independent samples). Results are presented as mean ± SD. Statistical significance was assessed using one-way ANOVA with Tukey's post-test. *P < 0.05.

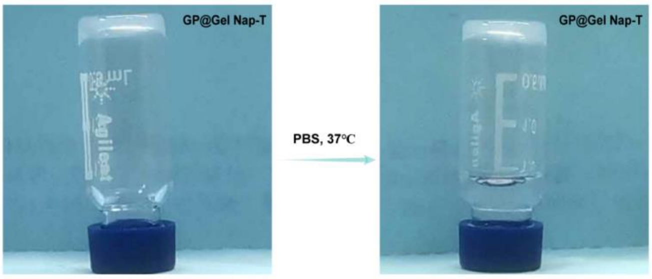

Figure S18. Optical images of 1.0 wt% **GP@Gel Nap-T** before and after incubation with PBS overnight at 37 °C, respectively.

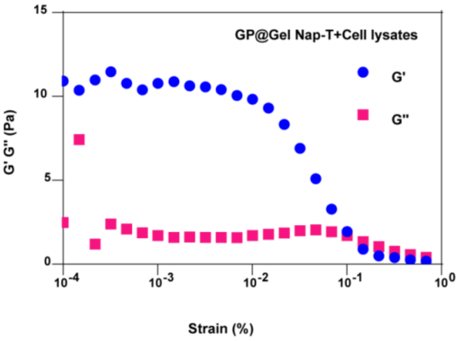

Figure S19. Strain dependence of the dynamic storage moduli (G´) and the loss moduli (G´´) of 1.0 wt% **GP@Gel Nap-T** (25 °C, frequency: 1 Hz).

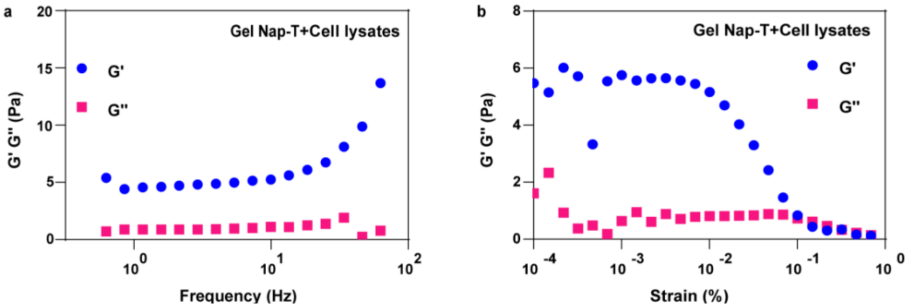

Figure S20. (a) Frequency dependence of the dynamic storage moduli (G´) and the loss moduli (G´´) of 1.0 wt% **Gel Nap-T** (25 °C, strain: 1.0%). **(b)** Strain dependence of the dynamic storage moduli (G´) and the loss moduli (G´´) of 1.0 wt% **Gel Nap-T** (25 °C, frequency: 1 Hz).

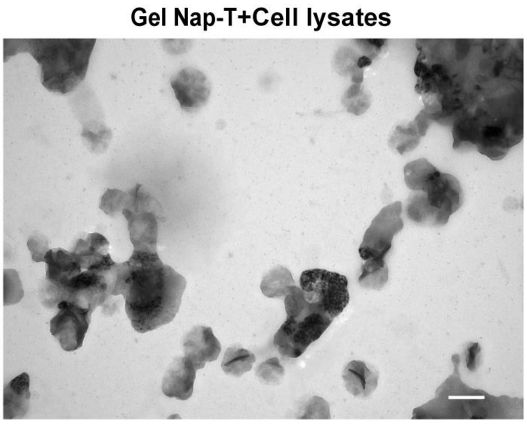

Figure S21. TEM image of **Gel Nap-T** after incubation with cell lysates overnight at 37 °C. Scale bar, 0.5 μm.

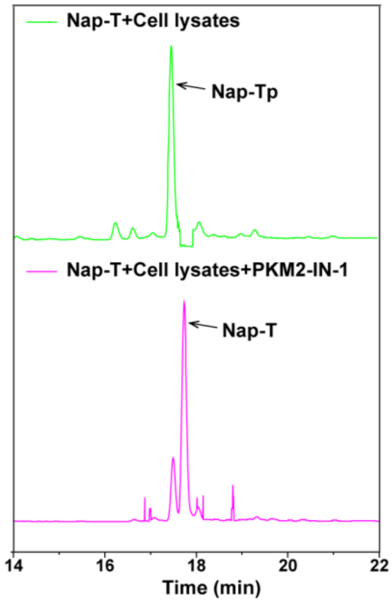

Figure S22. HPLC traces of **Nap-T** (cyan) and **Nap-T**+PKM2-IN-1 (purple) incubated with cell lysates at 37 °C for 24 h. Wavelength for detection: 220 nm.

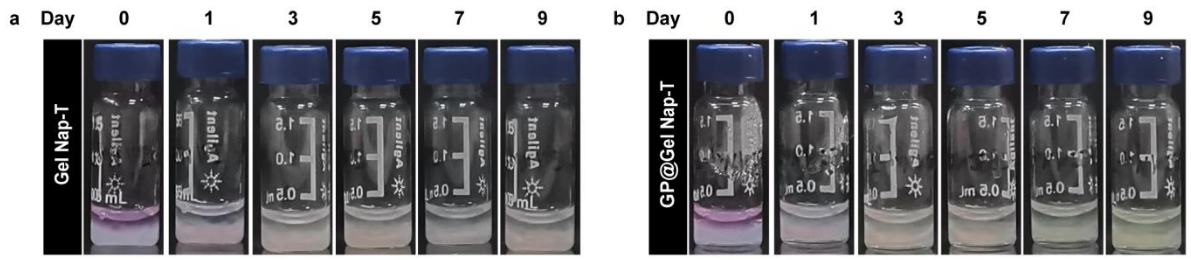

Figure S23. Optical images of 1.0 wt% **Gel Nap-T** (a) and **GP@Gel Nap-T** (b) after incubation with DMEM medium for 0 day, 1 day, 3 day, 5 day, 7 day and 9 day at 37 °C, respectively.

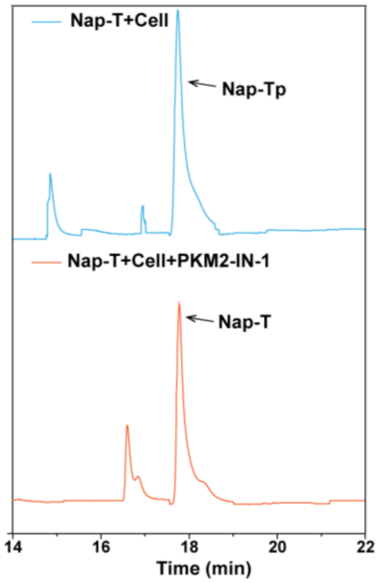

Figure S24. HPLC traces of **Nap-T** (light blue) and **Nap-T**+PKM2-IN-1 (brown) incubated with CMT167 cells culture medium at 37 °C for 24 h. Wavelength for detection: 220 nm.

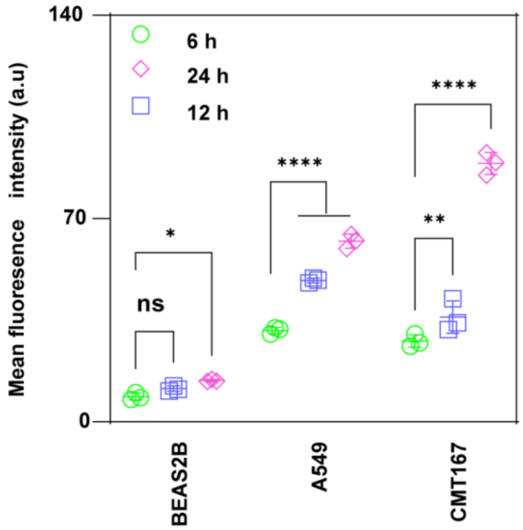

Figure S25. Corresponding average fluorescence intensity in Figure 1i (n = 3, biological independent samples). Results are presented as mean ± SD. Statistical significance was assessed using one-way ANOVA with Tukey's post-test. ns: no significant difference, *P < 0.05, **P < 0.01, ****P < 0.0001.

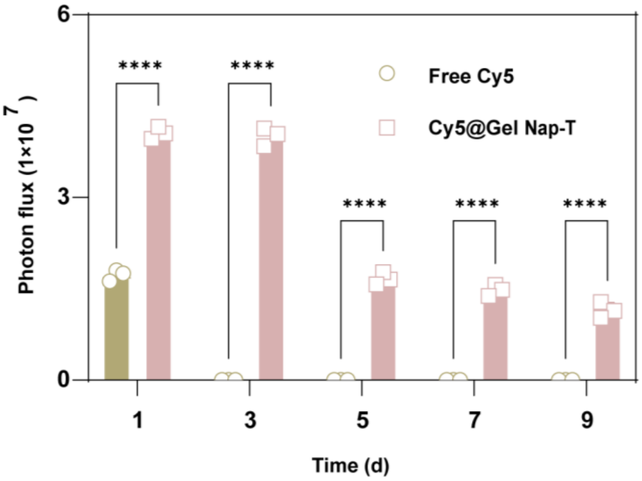

Figure S26. Corresponding average fluorescence intensity in Figure 1j, k (n = 3, biological independent samples). Results are presented as mean ± SD. Statistical significance was assessed using one-way ANOVA with Tukey's post-test. ****P < 0.0001, vs control group.

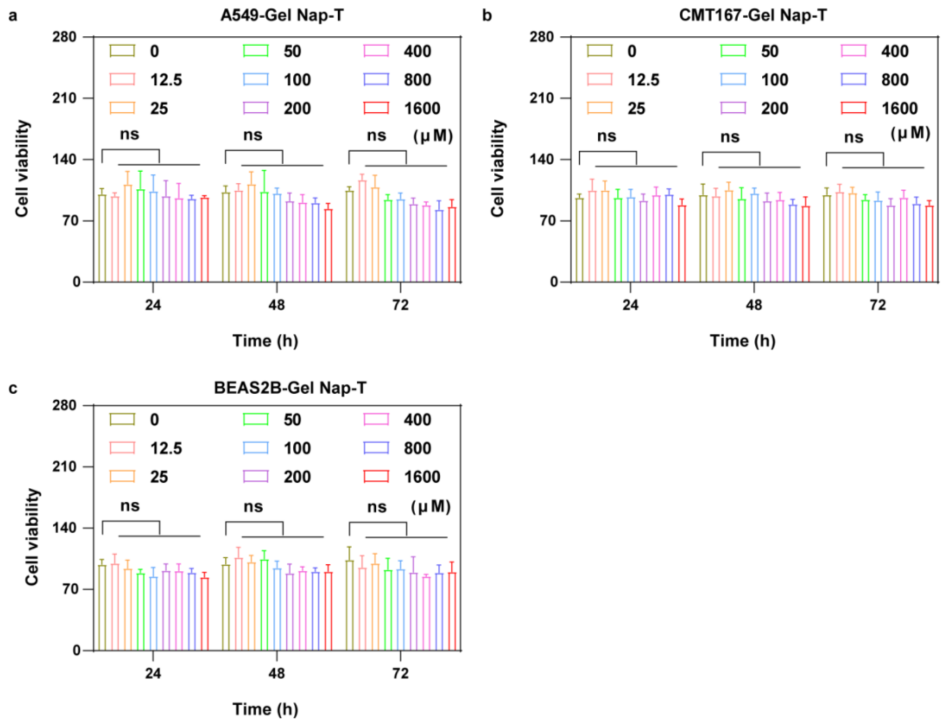

Figure S27. Cell viability assay of (a) A549, (b) CMT167 and (c) BEAS2B cells incubated with **Gel Nap-T** (**Nap-T**: 0-1600 μM) for 24, 48, or 72 h (n = 3, biological independent samples). Results are presented as mean ± SD. ns: no significant difference.

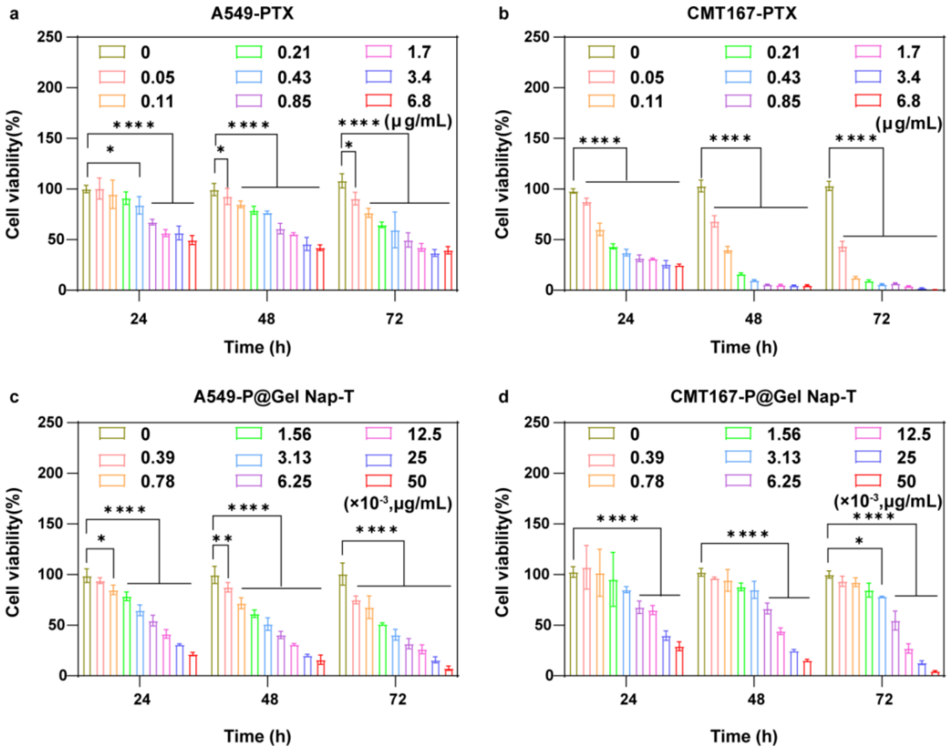

Figure S28. Cell viability of A549 cells and CMT167 cells incubated with (a, b) PTX and (c, d) **P@Gel Nap-T**, respectively, for 24, 48, or 72 h (n = 3, biological independent samples). Results are presented as mean ± SD. Statistical significance was assessed using one-way ANOVA with Tukey's post-test. *P < 0.05, **P < 0.01, ****P < 0.0001.

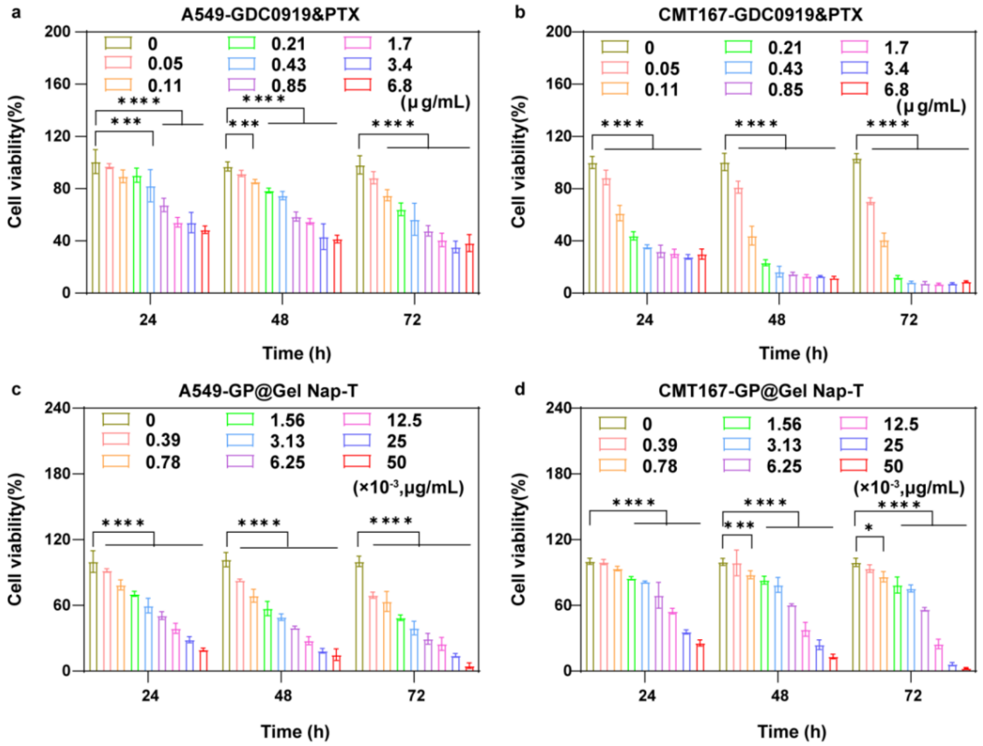

Figure S29. Cell viability of A549 cells and CMT167 cells incubated with (a, b) GDC0919&PTX and (c, d) **GP@Gel Nap-T**, respectively, for 24, 48, or 72 h (n = 3, biological independent samples). Results are presented as mean ± SD. Statistical significance was assessed using one-way ANOVA with Tukey's post-test. *P < 0.05, ***P < 0.001, ****P < 0.0001.

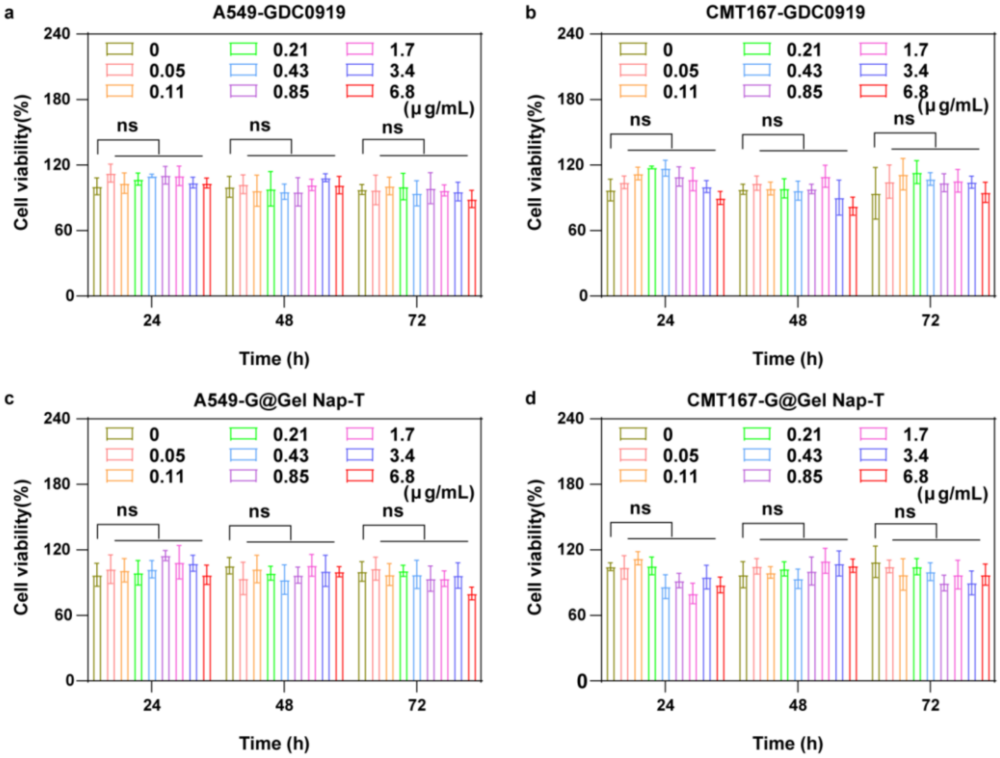

Figure S30. Cell viability of A549 cells and CMT167 cells incubated with (a, b) GDC0919 and (c, d) **G@Gel Nap-T**, respectively, for 24, 48, or 72 h (n = 3, biological independent samples). Results are presented as mean ± SD. ns: no significant difference.

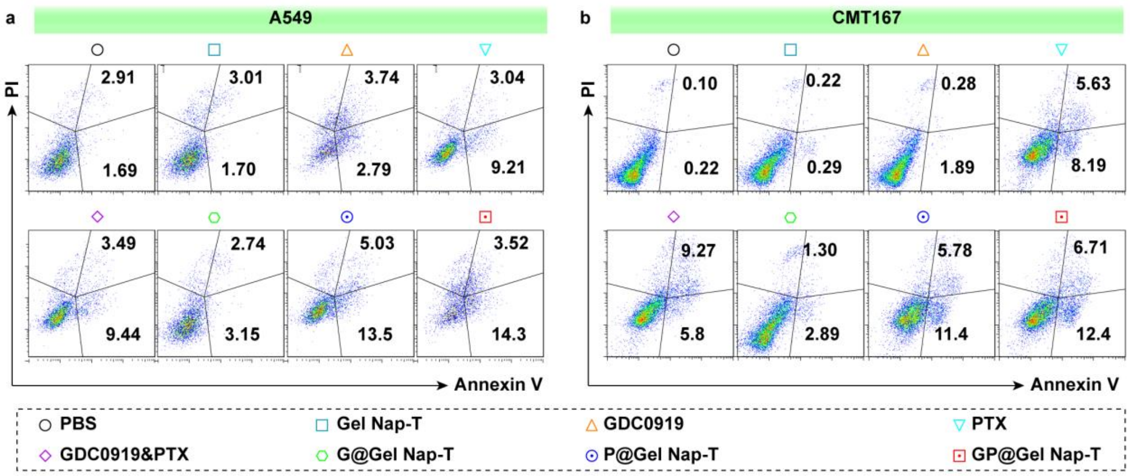

Figure S31. Representative FCM analysis of (a) A549 and (b) CMT167 cells after treatment with PBS, GDC0919, PTX, GDC0919&PTX, **Gel Nap-T**, **G@Gel Nap-T**, **P@Gel Nap-T** and **GP@Gel Nap-T** for 48 h stained with PI and Annexin V.

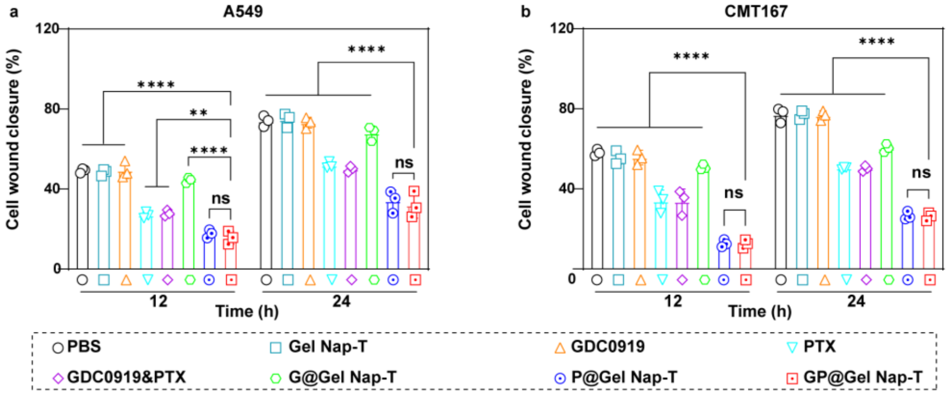

Figure S32. Quantitative results of scratch wound healing on (a) A549 and (b) CMT167 cells after different treatments in Figure 2e (n = 3, biological independent samples). Results are presented as mean ± SD. Statistical significance was assessed using one-way ANOVA with Tukey's post-test. ns: no significant difference, ** P < 0.01, **** P < 0.0001.

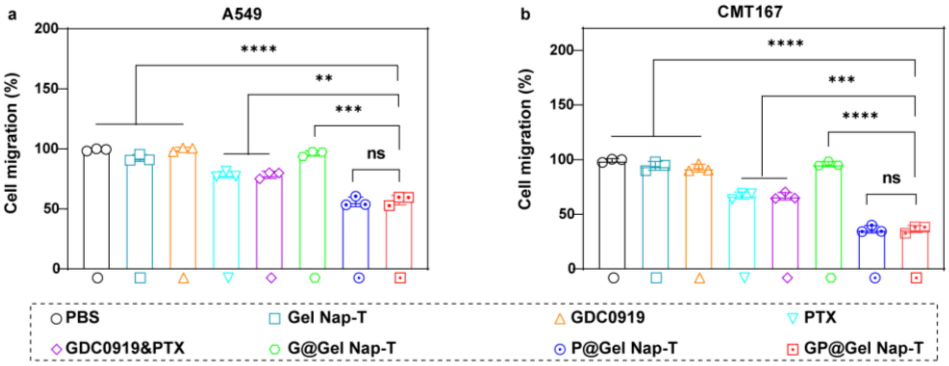

Figure S33. Quantitative results of transwell migration on (a) A549 and (b) CMT167 cells after different treatments in Figure 2g (n = 3, biological independent samples). Results are presented as mean ± SD. Statistical significance was assessed using one-way ANOVA with Tukey's post-test. ns: no significant difference, ** P < 0.01, ***P < 0.001, ****P < 0.0001.

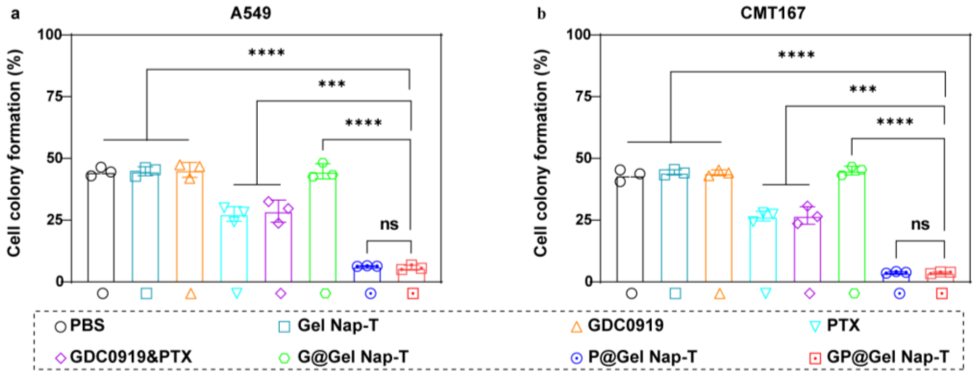

Figure S34. Quantitative results of cell clonogenicity on (a) A549 and (b) CMT167 cells after different treatments in Figure 2j for 14 d (n = 3, biological independent samples). Results are presented as mean ± SD. Statistical significance was assessed using one-way ANOVA with Tukey's post-test. ns: no significant difference, ***P < 0.001, ****P < 0.0001.

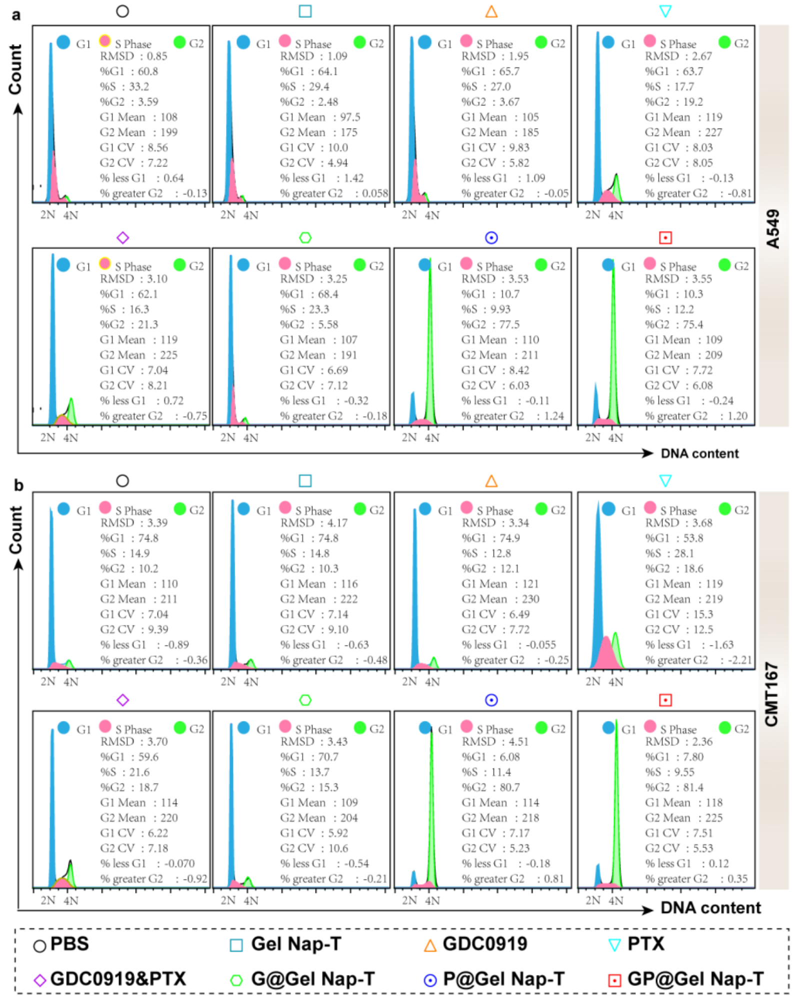

Figure S35. Representative FCM analysis of cell cycle on (a) A549 and (b) CMT167 cells after treatment with PBS, GDC0919, PTX, GDC0919&PTX, **Gel Nap-T**, **G@Gel Nap-T**, **P@Gel Nap-T** and **GP@Gel Nap-T** for 48 h.

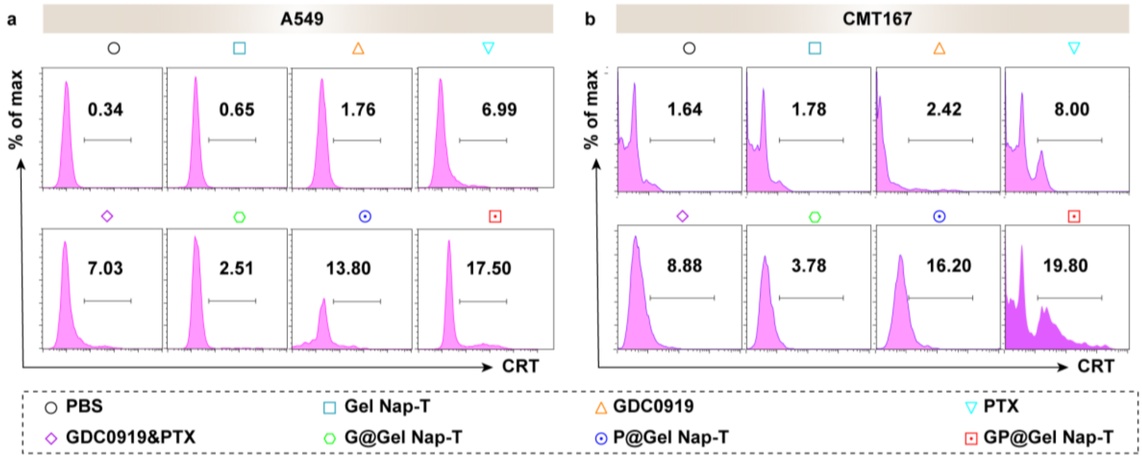

Figure S36. Representative FCM analysis of CRT on (a) A549 and (b) CMT167 cells after treatment with PBS, GDC0919, PTX, GDC0919-PTX, **Gel Nap-T**, **G@ Gel Nap-T**, **P@Gel Nap-T** and **GP@ Gel Nap-T** for 12 h.

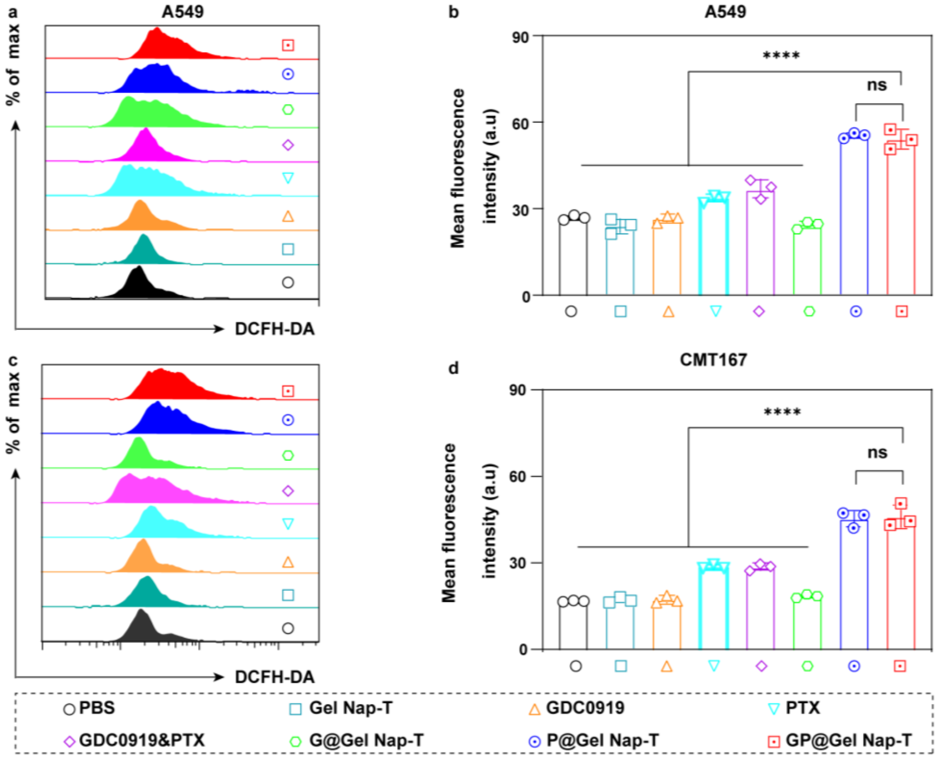

Figure S37. (a, c) Representative FCM and (b, d) corresponding quantitative data of intracellular ROS levels in A549 and CMT167 cells after treatment with PBS, GDC0919, PTX, GDC0919&PTX, **Gel Nap-T**, **G@Gel Nap-T**, **P@Gel Nap-T** and **GP@Gel Nap-T** for 48 h (n = 3, biological independent samples). Results are presented as mean ± SD. Statistical significance was assessed using one-way ANOVA with Tukey's post-test. ns: no significant difference, ****P < 0.0001.

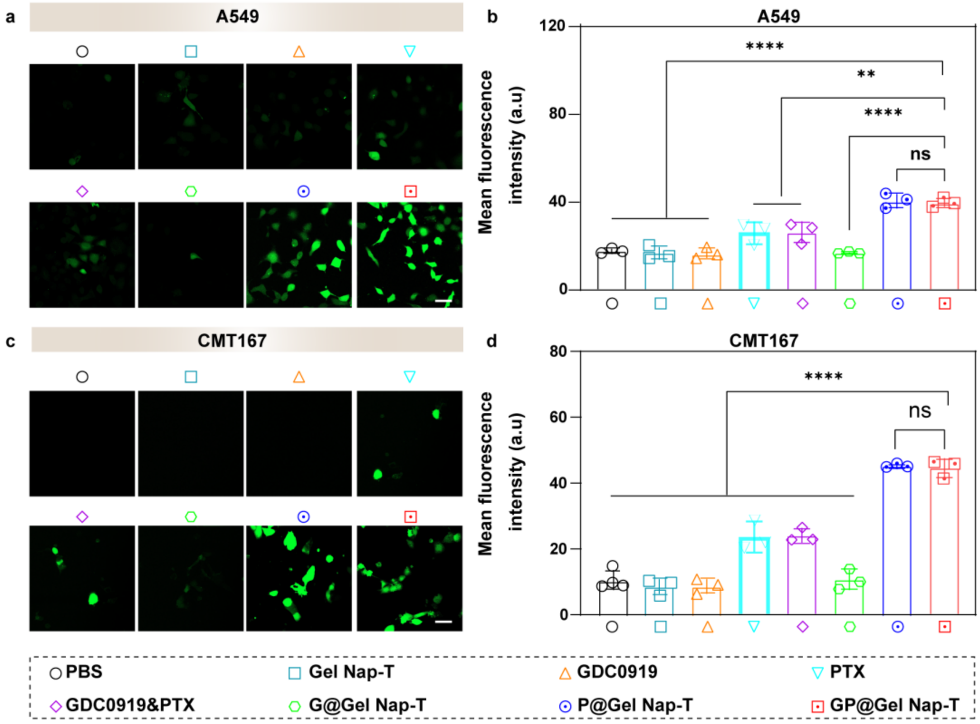

Figure S38. (a, c) Representative CLSM images and (b, d) corresponding quantification results of intracellular ROS levels in A549 and CMT167 cells after treatment with PBS, GDC0919, PTX, GDC0919&PTX, **Gel Nap-T**, **G@Gel Nap-T**, **P@Gel Nap-T** and **GP@Gel Nap-T** for 48 h. DCFH-DA (green). Scale bars, 50 μm (n = 3, biological independent samples). Results are presented as mean ± SD. Statistical significance was assessed using one-way ANOVA with Tukey's post-test. ns: no significant difference, **P < 0.01, ****P < 0.0001.

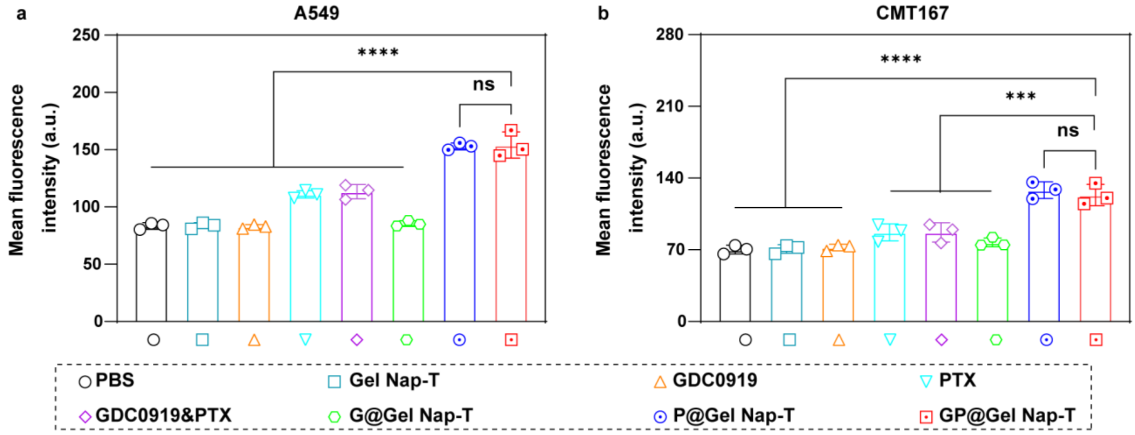

Figure S39. Quantitative FCM analysis of MitoSOX in (a) A549 and (b) CMT167 cells after different treatments in Figure 3d (n = 3, biological independent samples). Results are presented as mean ± SD. Statistical significance was assessed using one-way ANOVA with Tukey's post-test. ns: no significant difference, ***P < 0.001, ****P < 0.0001.

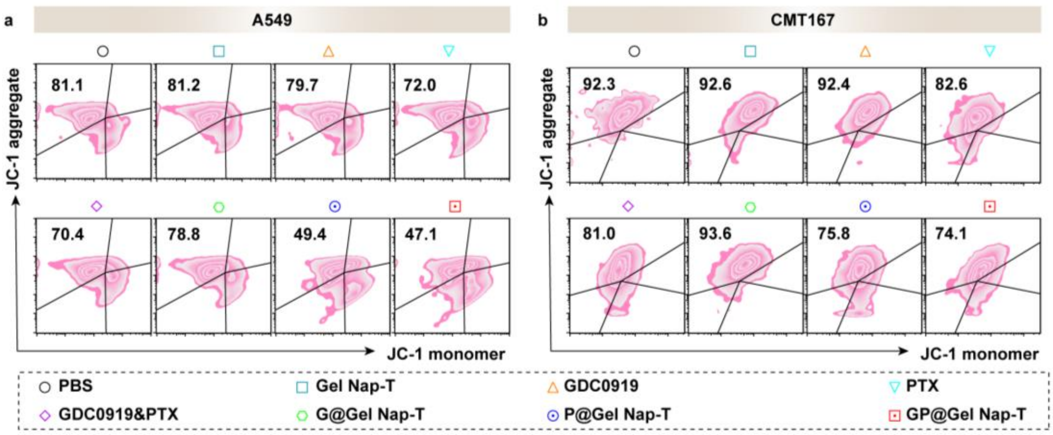

Figure S40. Representative FCM analysis of MMP in (a) A549 and (b) CMT167 cells after treatment with PBS, GDC0919, PTX, GDC0919&PTX, **Gel Nap-T**, **G@Gel Nap-T**, **P@Gel Nap-T** and **GP@Gel Nap-T** for 48 h.

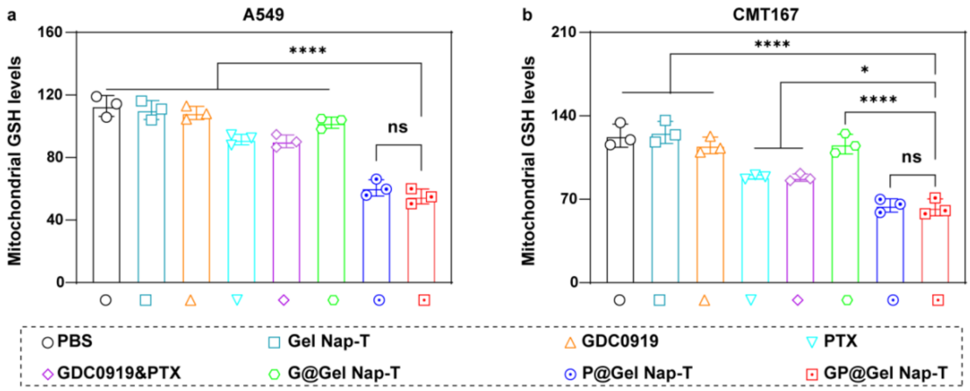

Figure S41. Quantitative data of mitochondrial GSH levels in (a) A549 and (b) CMT167 cells after treatment with PBS, GDC0919, PTX, GDC0919&PTX, **Gel Nap-T**, **G@Gel Nap-T**, **P@Gel Nap-T** and **GP@Gel Nap-T** for 48 h (n = 3, biological independent samples). Results are presented as mean ± SD. Statistical significance was assessed using one-way ANOVA with Tukey's post-test. ns: no significant difference, *P < 0.05, ****P < 0.0001.

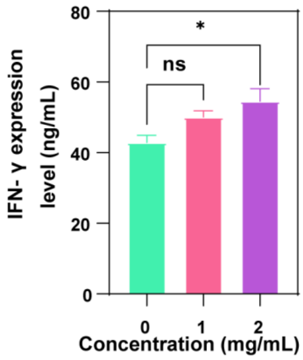

Figure S42. ELISA quantitative analysis of IFN-γ expression in CMT167-bearing tumor tissues following PTX treatment (n = 3, biological independent samples). Results are presented as mean ± SD. Statistical significance was assessed using one-way ANOVA with Tukey's post-test. ns: no significant difference, *P < 0.05.

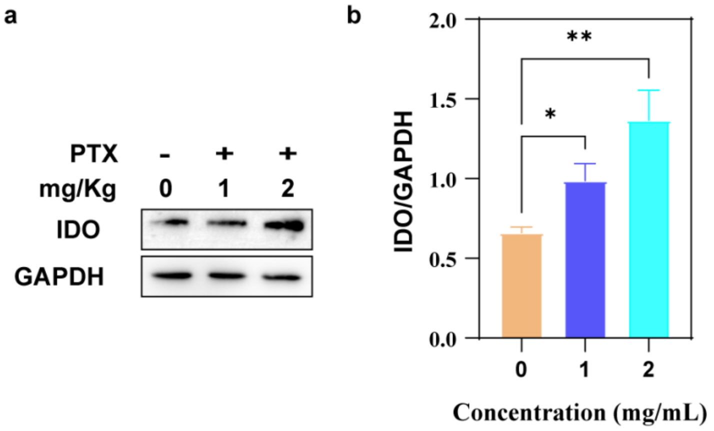

Figure S43. (a) WB results and (b) corresponding quantification results of the expression levels of IDO in CMT167 tumor tissues after PTX treatment. Control, GAPDH (n = 3, biological independent samples). Results are presented as mean ± SD. Statistical significance was assessed using one-way ANOVA with Tukey's post-test. *P < 0.05, **P < 0.01.

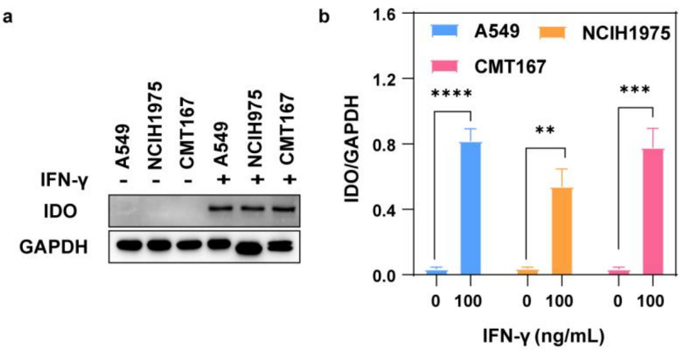

Figure S44. (a) WB results and (b) corresponding quantification results of the expression levels of IDO in A549, NCIH1975, and CMT167 cells after 48 h with or without recombinant murine IFN-γ treatment. Control, GAPDH (n = 3, biological independent samples). Results are presented as mean ± SD. Statistical significance was assessed using one-way ANOVA with Tukey's post-test. **P < 0.01, ***P < 0.001, ****P < 0.0001.

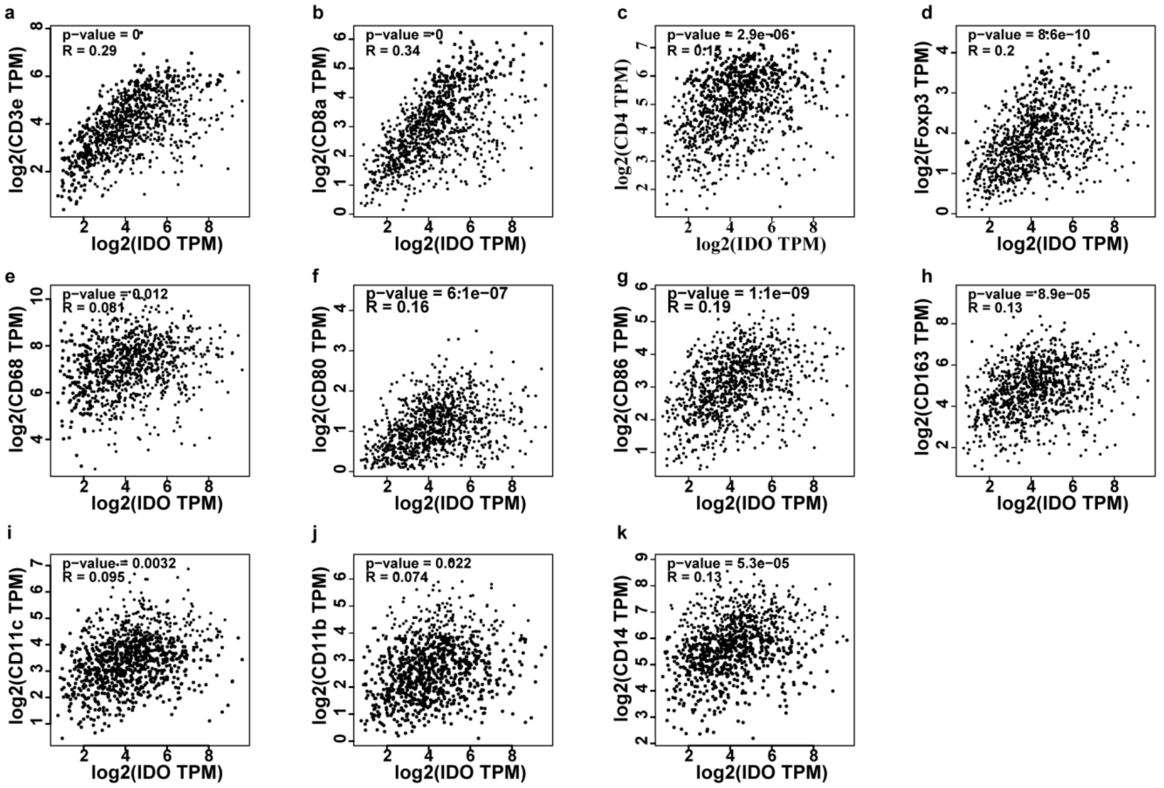

Figure S45. Correlation analysis of between IDO expression and (a) CD3e, (b) CD8α, (c) CD4, (d) Foxp3, (e) CD68, (f) CD80, (g) CD86, (h) CD163, (i) CD11c, (j) CD11b and (k) CD14 expressions sourced from the TCGA database are shown for NSCLC using Spearman's rank test.

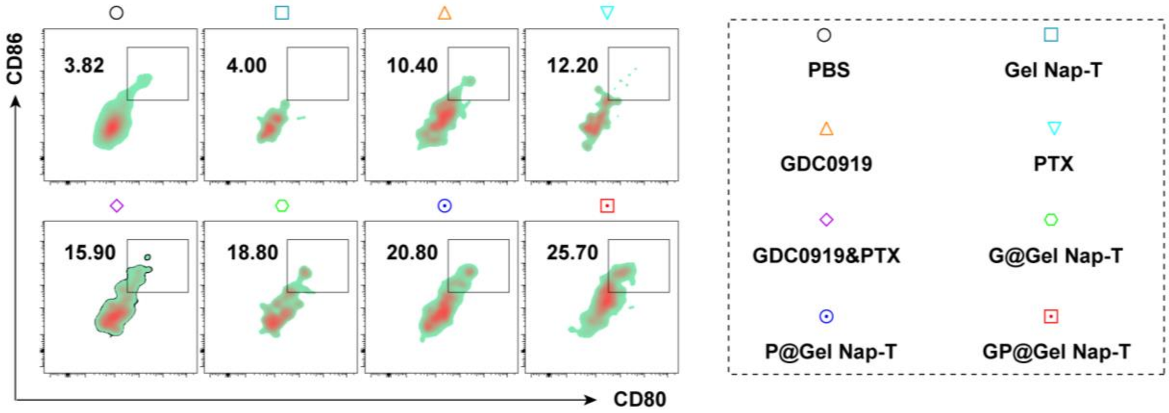

Figure S46. Representative FCM analysis of mature DCs. BMDCs stimulated with recombinant murine GM-CSF and recombinant murine IL-4 for 7 days to acquire immature DCs. Then, immature DCs were co-culture with CMT167 cells and treated with PBS, GDC0919, PTX, GDC0919&PTX, **Gel Nap-T**, **G@Gel Nap-T**, **P@Gel Nap-T** and **GP@Gel Nap-T** for 24 h.

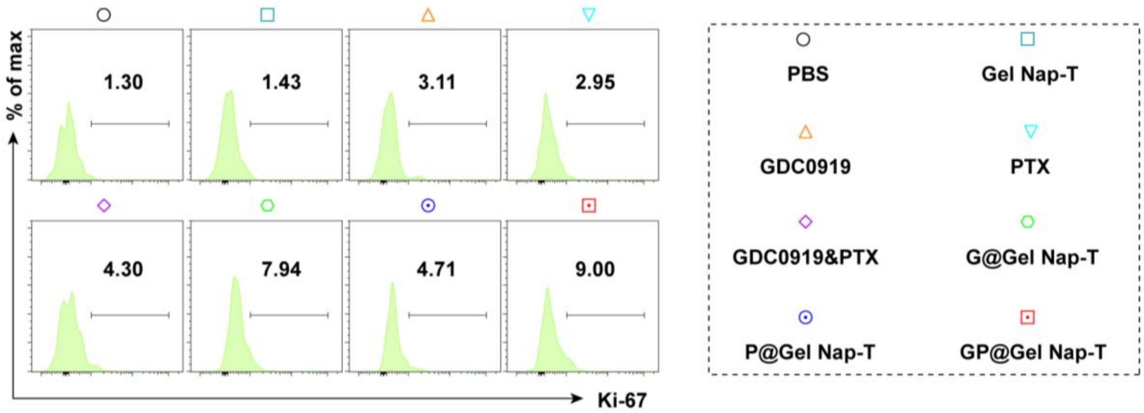

Figure S47. Representative FCM histograms of $CD4^+$ T cells proliferation. CMT167 cells were stimulated with IFN-γ for overnight and then treated with PBS, GDC0919, PTX, GDC0919&PTX, **Gel Nap-T**, **G@Gel Nap-T**, **P@Gel Nap-T** and **GP@Gel Nap-T** for 12 h. Pre-treated CMT167 cells and splenic lymphocytes were mixed, treated with soluble anti-CD3 antibody and recombinant murine IL-2 for another 48 h.

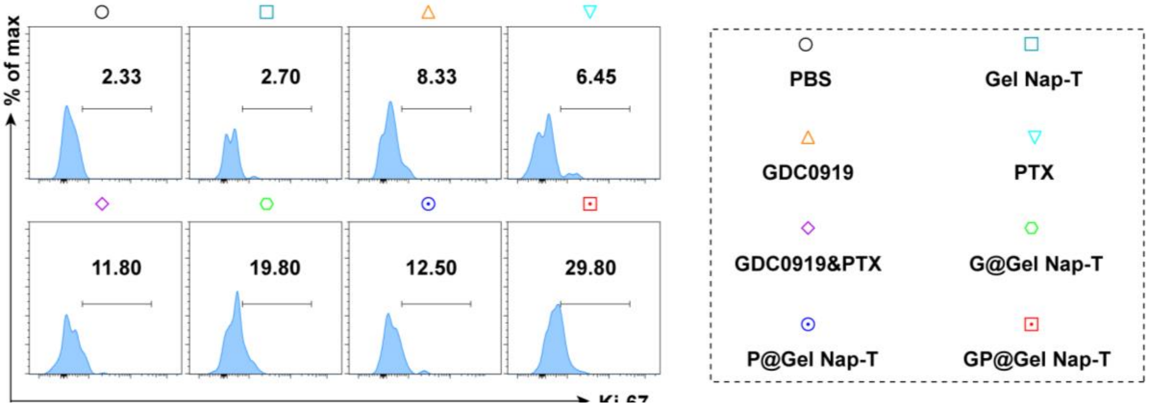

Figure S48. Representative FCM histograms of $CD8^+$ T cells proliferation. CMT167 cells were stimulated with IFN-γ for overnight and then treated with PBS, GDC0919, PTX, GDC0919&PTX, **Gel Nap-T**, **G@Gel Nap-T**, **P@Gel Nap-T** and **GP@Gel Nap-T** for 12 h. Pre-treated CMT167 cells and splenic lymphocytes were mixed, treated with soluble anti-CD3 antibody and recombinant murine IL-2 for another 48 h.

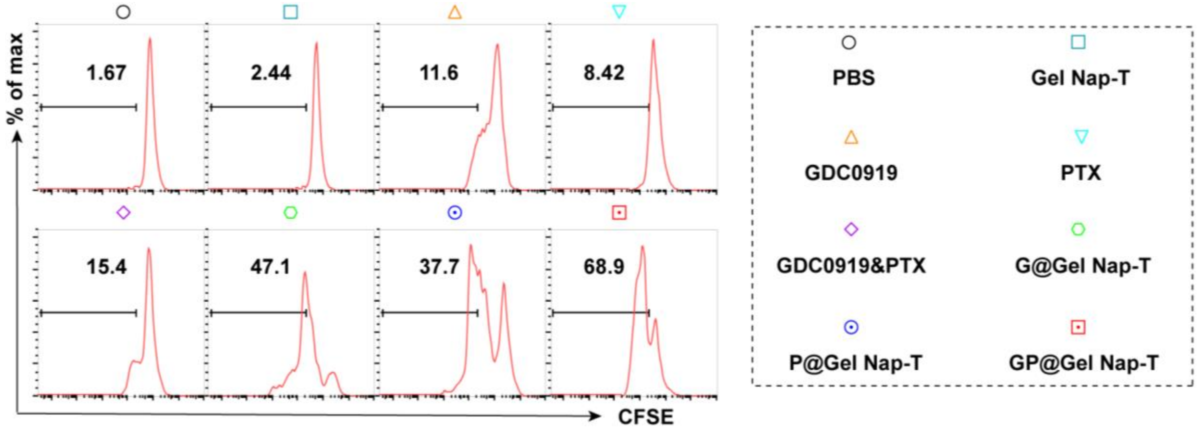

Figure S49. Representative FCM histograms of NK cells proliferation. CMT167 cells were

stimulated with IFN-γ for overnight and then treated with PBS, GDC0919, PTX, GDC0919&PTX, **Gel Nap-T**, **G@Gel Nap-T**, **P@Gel Nap-T** and **GP@Gel Nap-T** for 12 h. Pre-treated CMT167 cells and splenic lymphocytes stained with CFSE were mixed, treated with recombinant murine IL-2 and recombinant murine IL-15 for another 48 h.

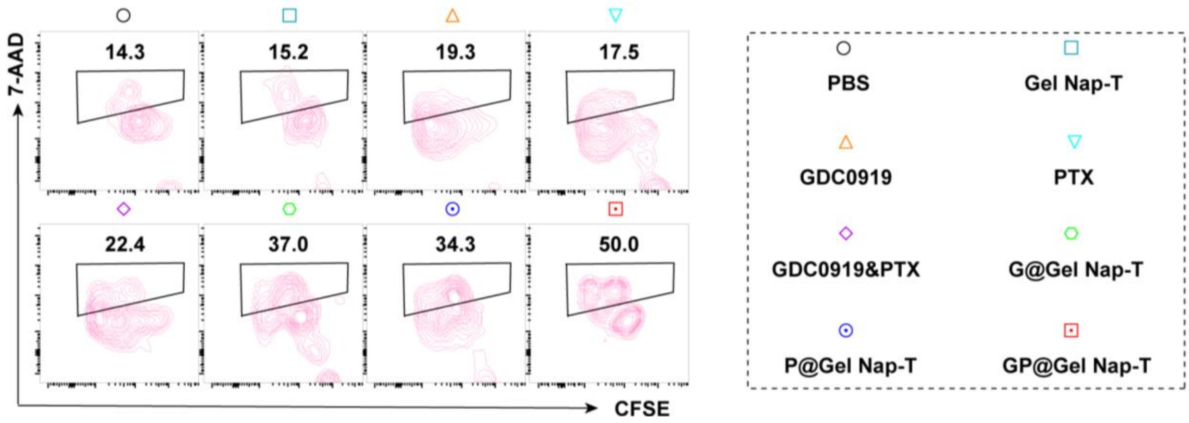

Figure S50. Representative FCM plots of dead CMT167 cells after T cell attacking. CMT167 cells were stimulated with recombinant murine IFN-γ for overnight and then treated with PBS, GDC0919, PTX, GDC0919&PTX, **Gel Nap-T**, **G@Gel Nap-T**, **P@Gel Nap-T** and **GP@Gel Nap-T** for 12 h. Afterward, CMT167 cells stained with CFSE and mixed with splenic lymphocytes, treated with soluble anti-CD3 antibody and recombinant murine IL-2 for 4 h.

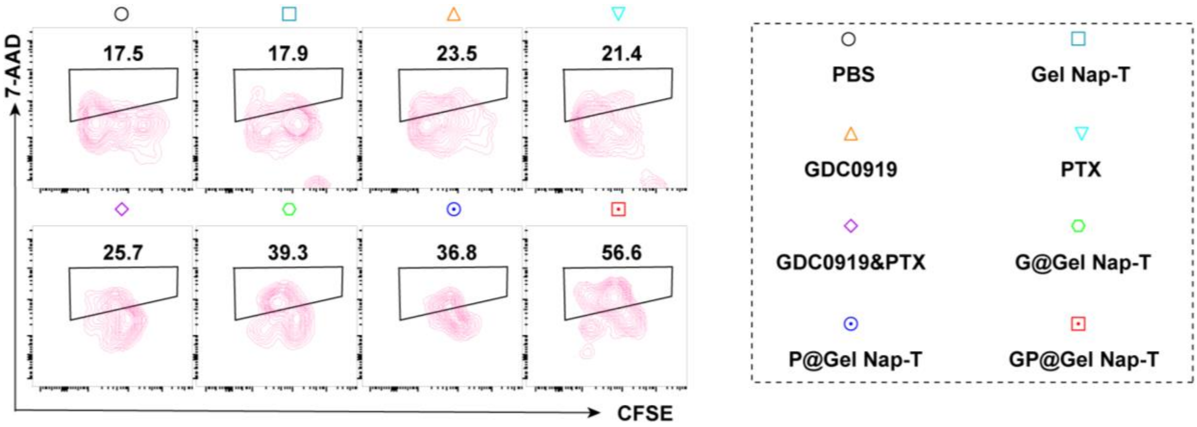

Figure S51. Representative FCM plots of dead CMT167 cells after NK cell attacking. CMT167 cells were stimulated with IFN-γ for overnight and then treated with PBS, GDC0919, PTX, GDC0919&PTX, **Gel Nap-T**, **G@Gel Nap-T**, **P@Gel Nap-T** and **GP@Gel Nap-T** for 12 h. Afterward, CMT167 cells stained with CFSE and mixed with splenic lymphocytes, treated with recombinant murine IL-2 and recombinant murine IL-15 for 4 h.

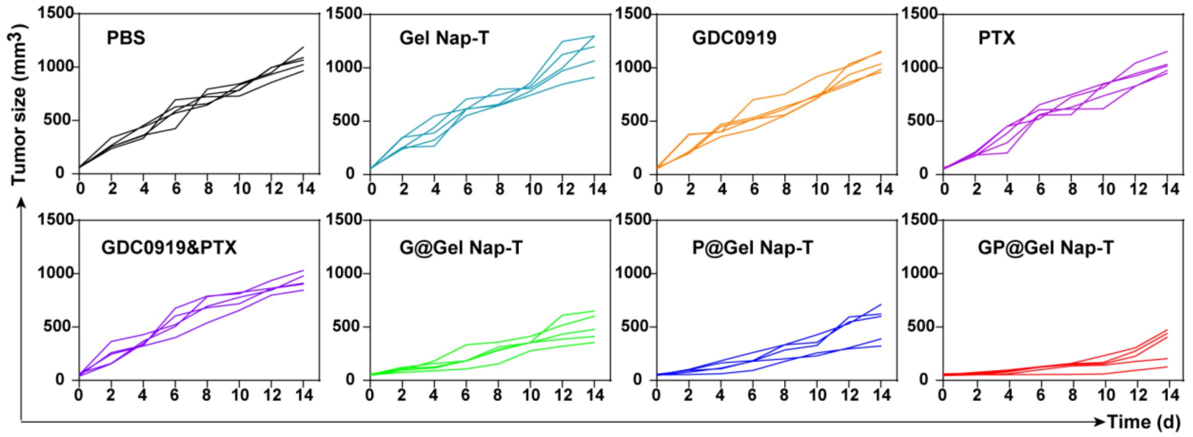

Figure S52. Individual tumor growth kinetics in various groups.

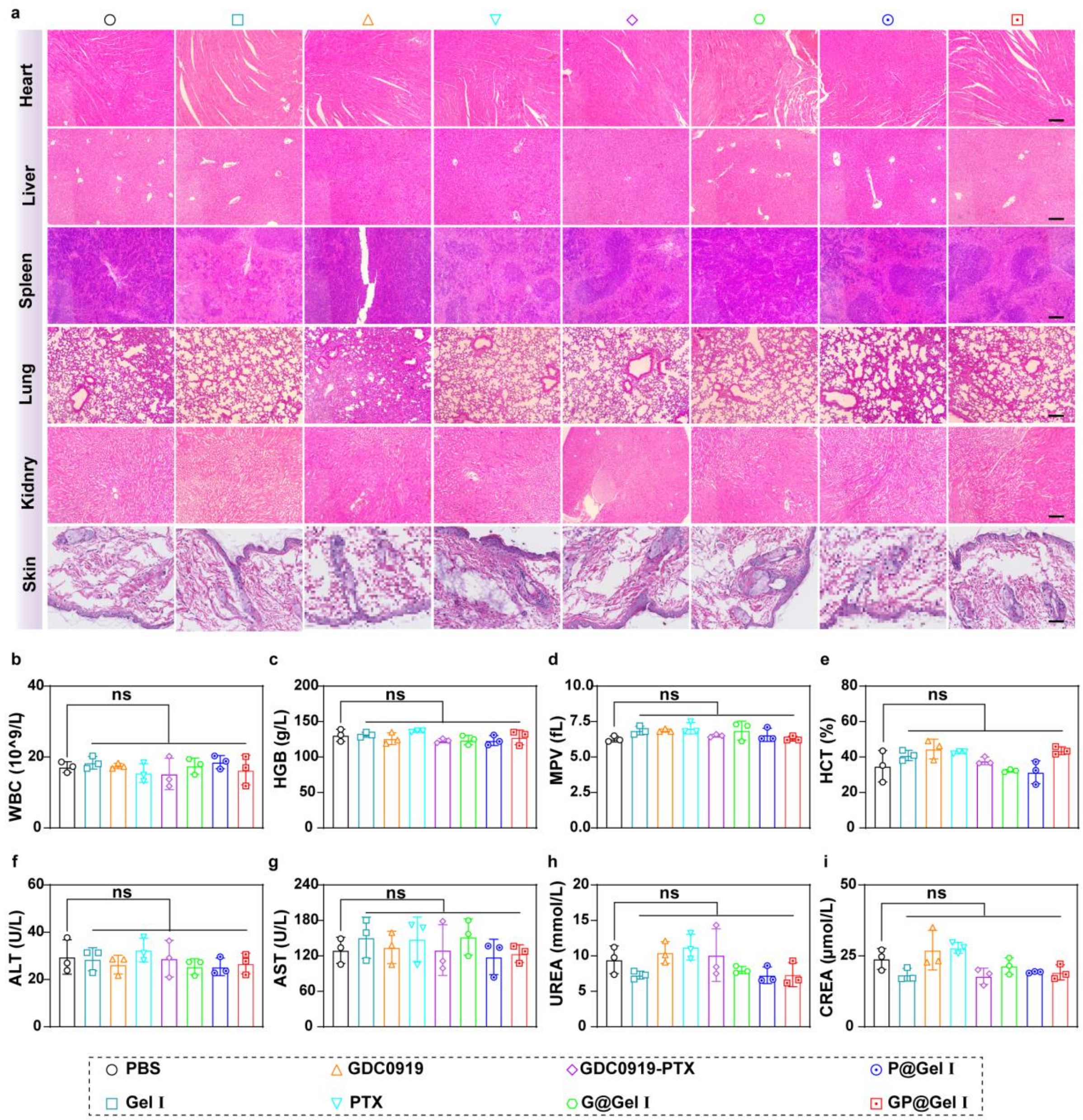

Figure S53. (a) H&E staining analysis of heart, liver, spleen, lung, kidney and skin from the mice in each treatment group on day 14. Scale bars, 50 μm . (b-e) Routine blood examinations:

(b) white blood cell (WBC), (c) hemoglobin (HGB), (d) mean platelet volume (MPV), (e) hematocrit (HCT) (n = 3, biological independent samples). (f-i) Biochemical analysis of serum: (f) alanine aminotransferase (ALT), (g) aspartate aminotransferase (AST), (h) blood urea nitrogen (BUN), (i) creatinine (CREA) (n = 3, biological independent samples). Results are presented as mean ± SD. Statistical significance was assessed using one-way ANOVA with Tukey's post-test. ns: no significant difference.

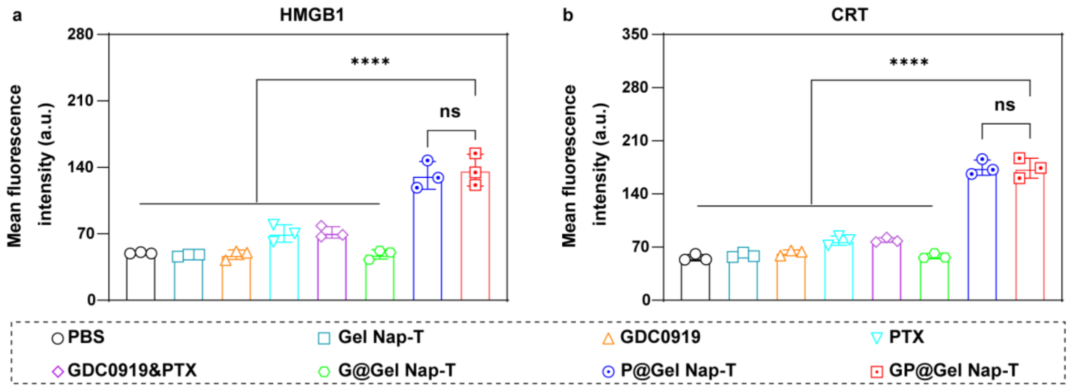

Figure S54. Corresponding quantitative data of (a) HMGB1 and (b) CRT in Figure 5a (n = 3, biological independent samples). Results are presented as mean ± SD. Statistical significance was assessed using one-way ANOVA with Tukey's post-test. ns: no significant difference, ****P< 0.0001.

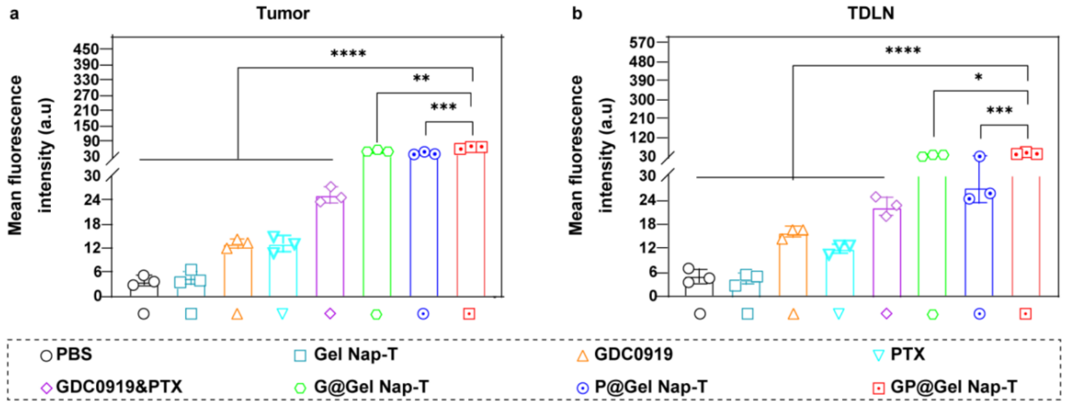

Figure S55. Corresponding quantitative data of DCs in (a) tumor and (b) TDLNs in Figure 5b (n = 3, biological independent samples). Results are presented as mean ± SD. Statistical significance was assessed using one-way ANOVA with Tukey's post-test. ns: no significant difference, *P < 0.05, **P < 0.01, ***P < 0.001, ****P < 0.0001.

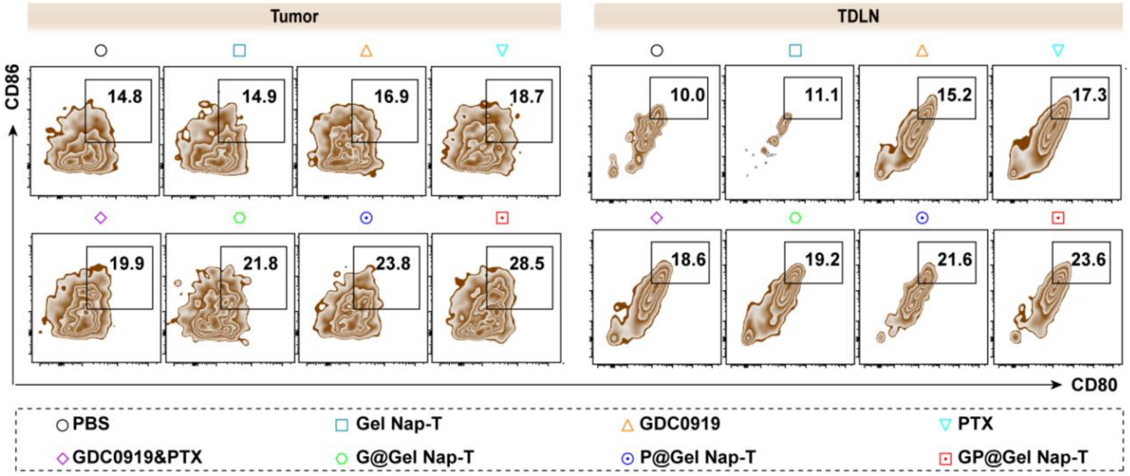

Figure S56. Representative FCM analysis of mature DCs in the tumor tissues and TDLNs from CMT167 tumor-bearing mice on day 14 after different treatments.

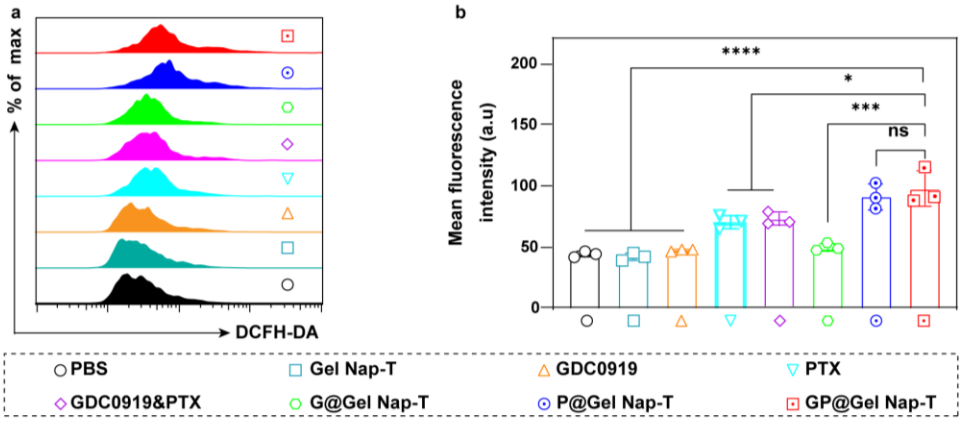

Figure S57. (a) Representative FCM analysis and (b) corresponding quantitative data of ROS generations in the tumor tissues from CMT167 tumor-bearing mice on day 14 after different treatments (n = 3, biological independent samples). Results are presented as mean ± SD. Statistical significance was assessed using one-way ANOVA with Tukey's post-test. ns: no significant difference, *P < 0.05, ***P < 0.001, ****P < 0.0001.

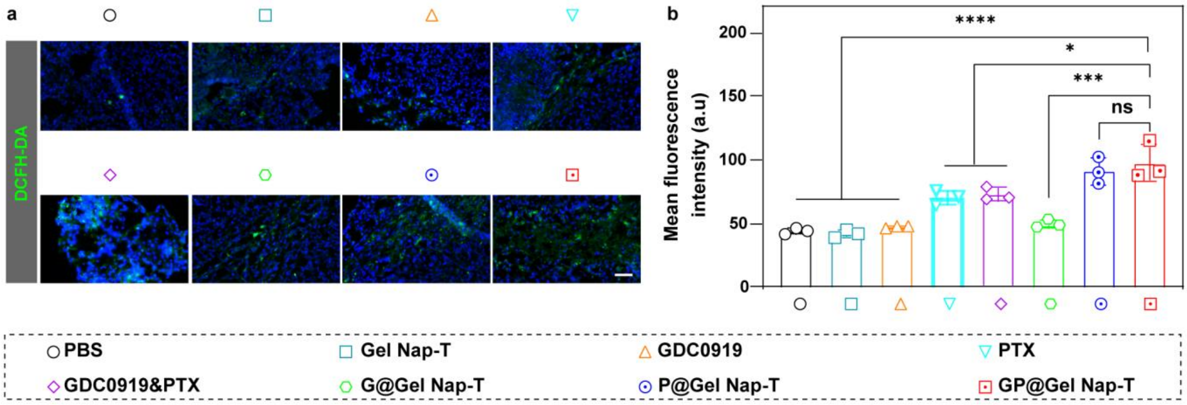

Figure S58. (a) Representative IF images and (b) corresponding quantitative data of ROS generations in the tumor tissues from CMT167 tumor-bearing mice on day 14 after different treatments. DAPI (blue), DCFH-DA (green). Scale bars, 50 μm (n = 3, biological independent

samples). Results are presented as mean ± SD. Statistical significance was assessed using one-way ANOVA with Tukey's post-test. ns: no significant difference, *P < 0.05, ***P < 0.001, ****P < 0.0001.

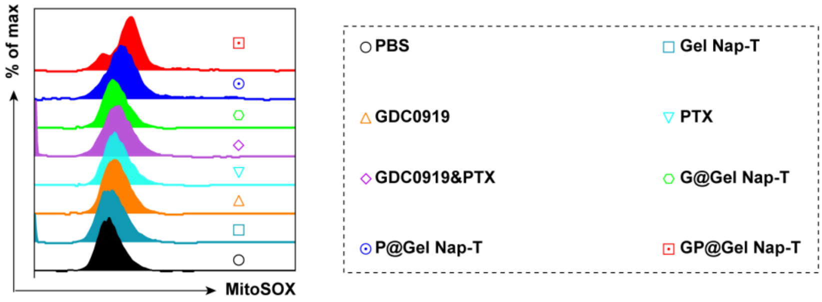

Figure S59.Representative FCM of MitoSOX in the tumor tissues from CMT167 tumor-bearing mice on day 14 after different treatments.

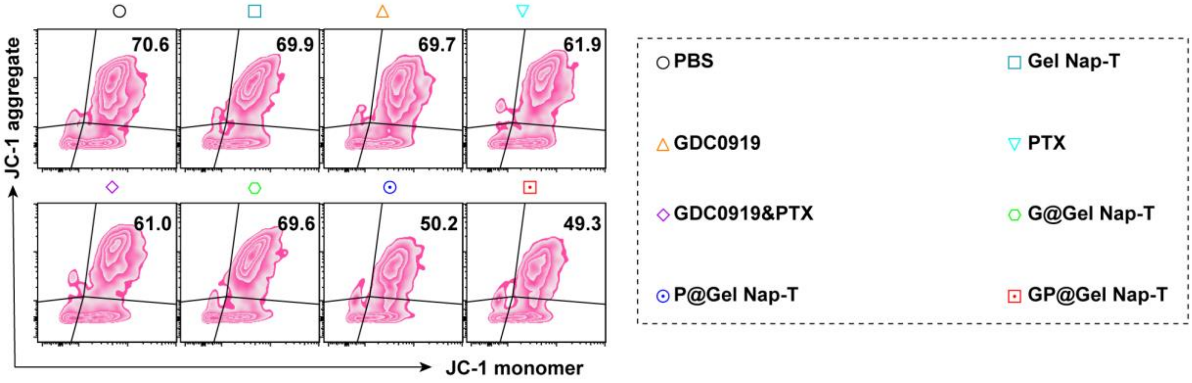

Figure S60. Representative FCM analysis of MMP of tumor cells from CMT167 tumor-bearing mice on day 14.

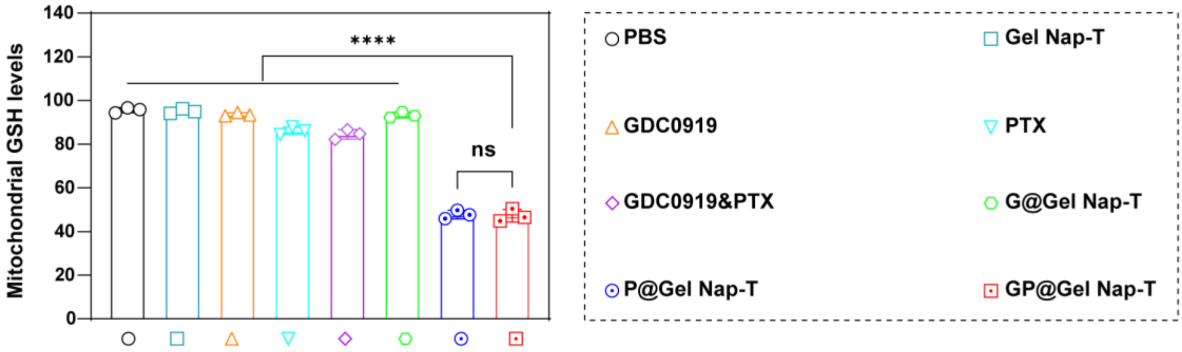

Figure S61. Quantitative data of mitochondrial GSH levels in in the tumor tissues from CMT167 tumor-bearing mice on day 14 after different treatments (n = 3, biological independent samples). Results are presented as mean ± SD. Statistical significance was assessed using one-way ANOVA with Tukey's post-test. ns: no significant difference, ****P < 0.0001.

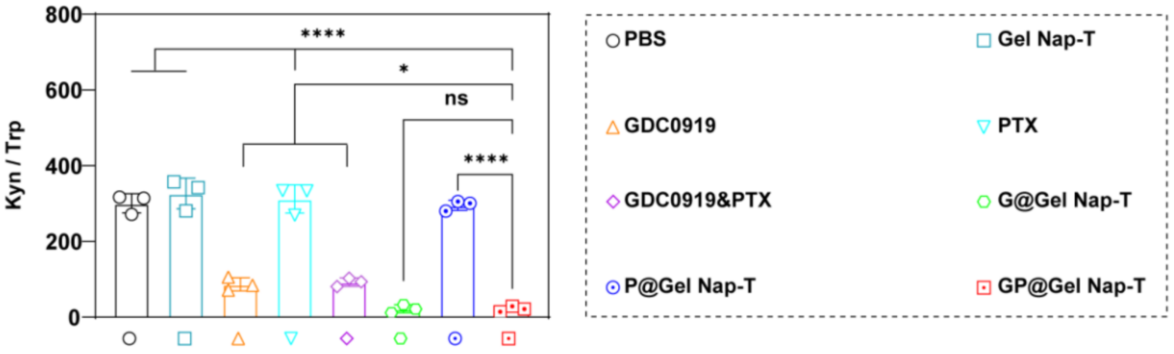

Figure S62. The ratio of Kyn/Trp concentrations in serum from CMT167 tumor-bearing mice on day 14 after different treatments (n = 3, biological independent samples). Results are presented as mean ± SD. Statistical significance was assessed using one-way ANOVA with Tukey's post-test. ns: no significant difference, *P < 0.05, ****P < 0.0001.

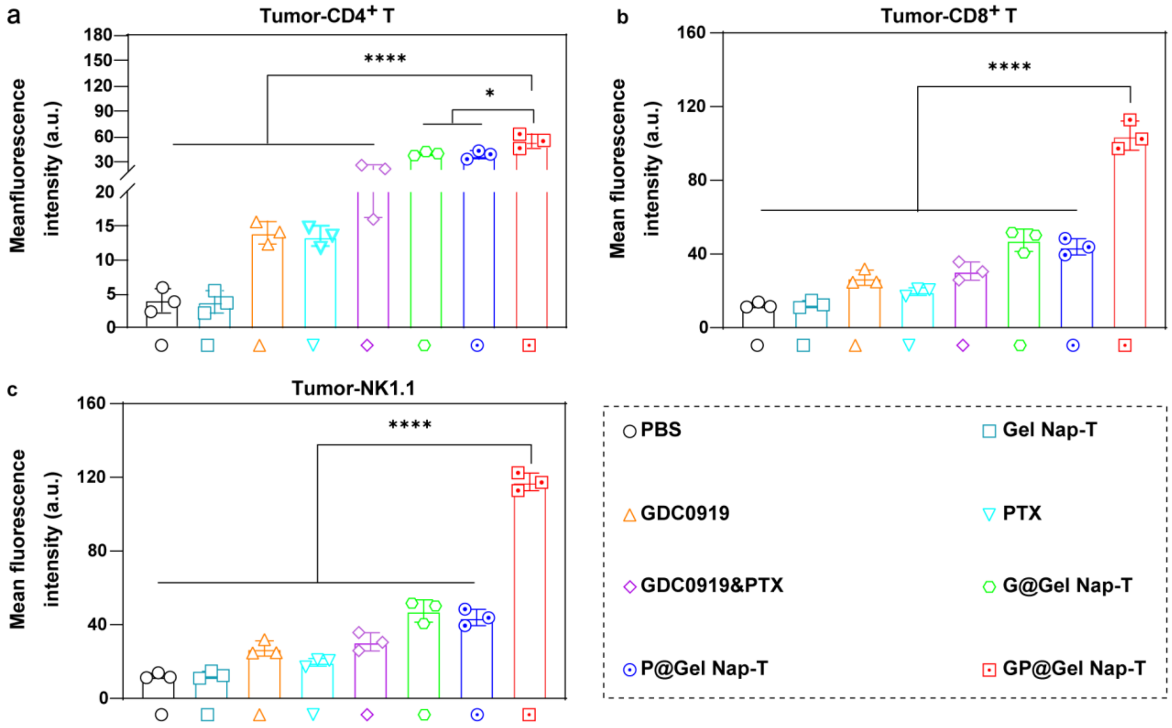

Figure S63.Corresponding quantitative data of (a) $CD4^+$ cells, (b) $CD8^+$ cells and (c) $NK1.1^+$ cells in Figure 6e (n = 3, biological independent samples). Results are presented as mean ± SD. Statistical significance was assessed using one-way ANOVA with Tukey's post-test. ns: no significant difference, *P < 0.05, ****P < 0.0001.

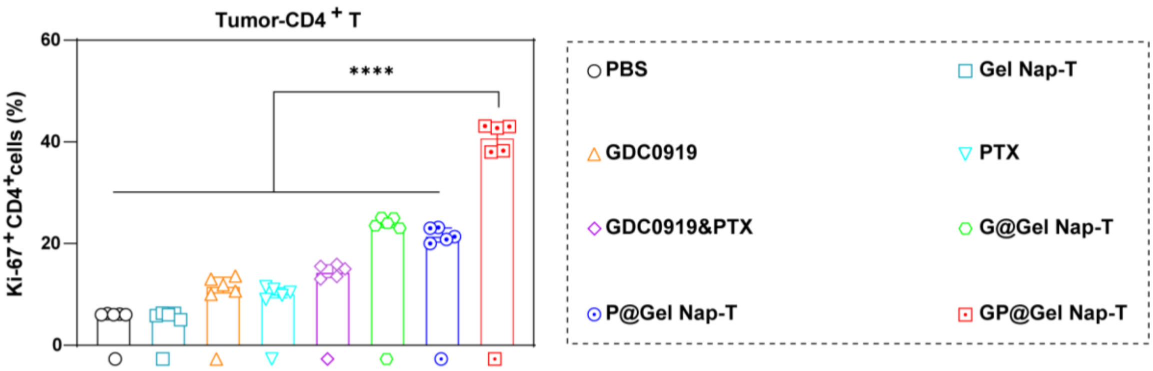

Figure S64. Quantitative analysis of the proportions of Ki-67$^+$CD4$^+$ T cells in the tumor tissues from CMT167 tumor-bearing mice on day 14 after different treatments (n = 5, biologically independent animals per group). Results are presented as mean ± SD. Statistical significance was assessed using one-way ANOVA with Tukey's post-test. ns: no significant difference, ****P < 0.0001.

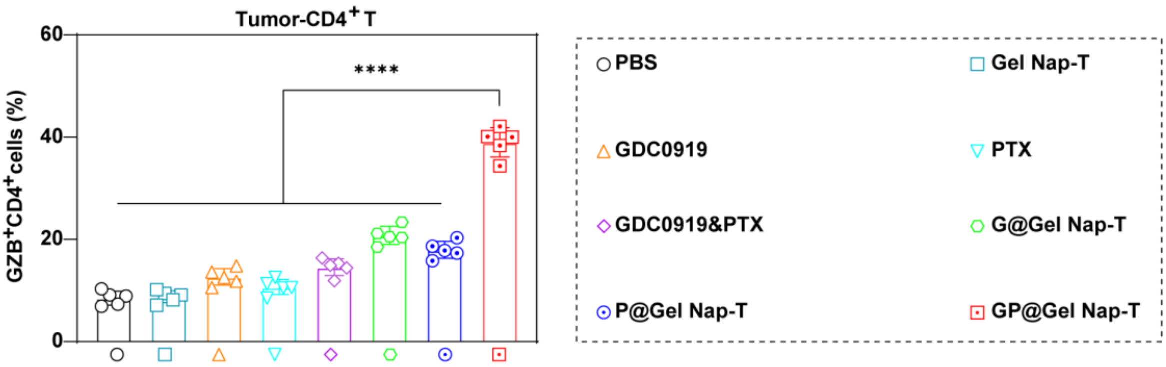

Figure S65. Quantitative analysis of the proportions of GZB$^+$CD4$^+$ T cells in the tumor tissues from CMT167 tumor-bearing mice on day 14 after different treatments (n = 5, biologically independent animals per group). Results are presented as mean ± SD. Statistical significance was assessed using one-way ANOVA with Tukey's post-test. ns: no significant difference, ****P < 0.0001.

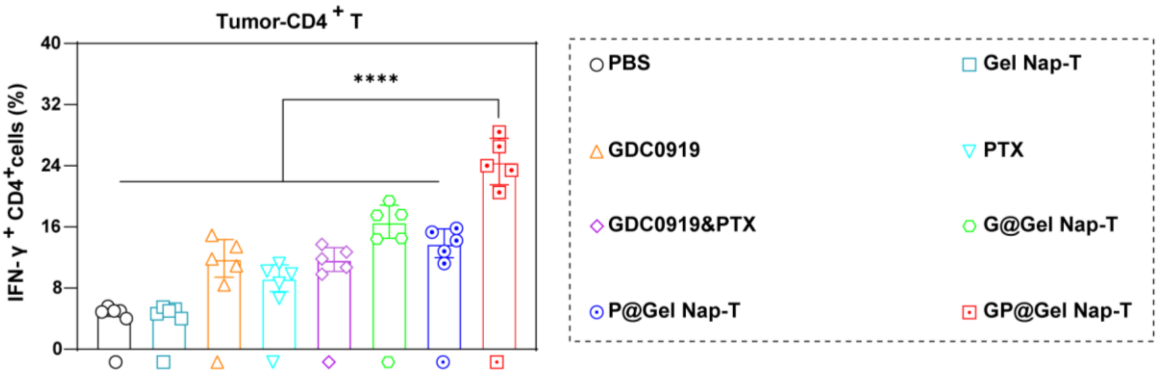

Figure S66. Quantitative analysis of the proportions of IFN-γ$^+$CD4$^+$ T cells in the tumor tissues from CMT167 tumor-bearing mice on day 14 after different treatments (n = 5, biologically independent animals per group). Results are presented as mean ± SD. Statistical significance

was assessed using one-way ANOVA with Tukey's post-test. ns: no significant difference, ****P < 0.0001.

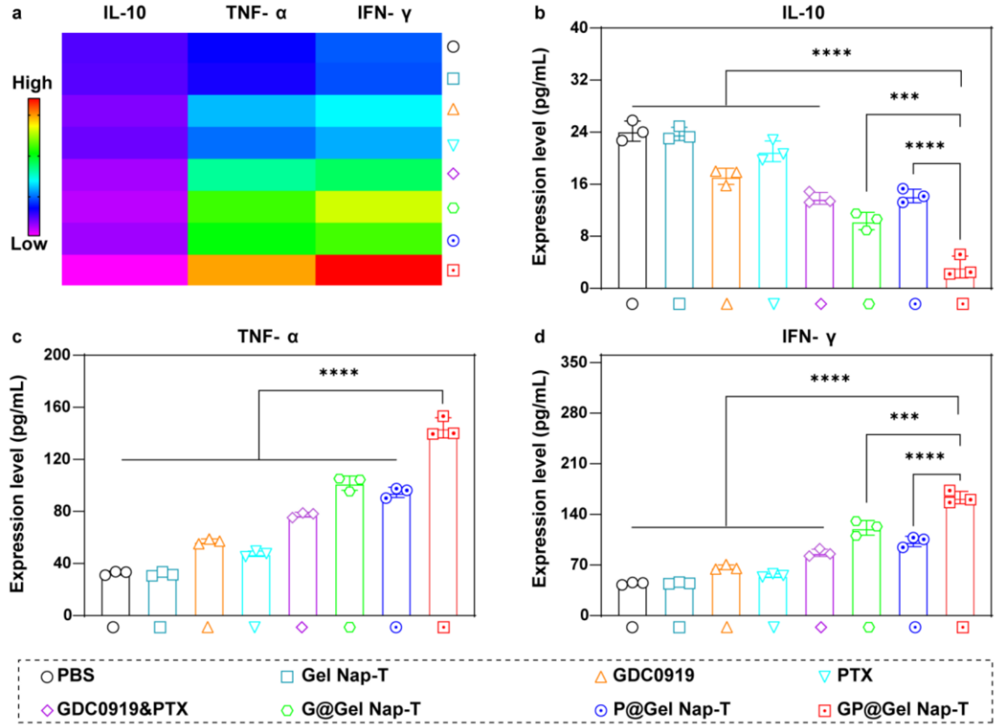

Figure S67. (a) Heatmap and (b-d) corresponding quantitative data of cytokine (IL-10, TNF-α and IFN-γ) expression levels in the tumor tissues from CMT167 tumor-bearing mice on day 14 after different treatments (n = 3). Results are presented as mean ± SD. Statistical significance was assessed using one-way ANOVA with Tukey's post-test. ns: no significant difference, ***P< 0.001, **** P< 0.0001.

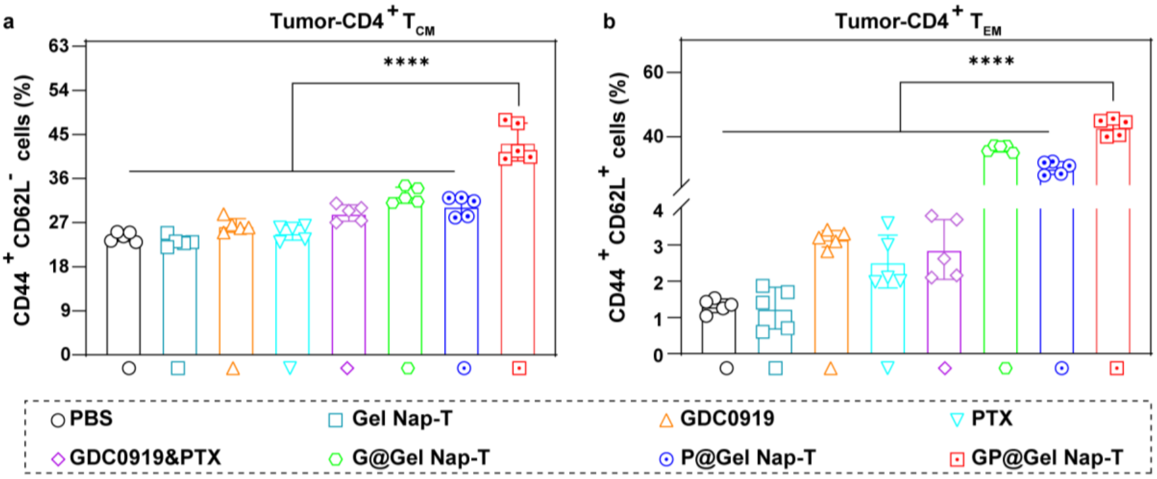

Figure S68. Quantitative analysis of the proportions of (a) $CD4^+$ T_{CM} ($CD44^+CD62L^+$) cells and (b) $CD4^+$ T_{EM} ($CD44^+CD62L^-$) cells in the tumor tissues from CMT167 tumor-bearing mice on day 14 after different treatments (n = 5). Results are presented as mean ± SD. Statistical significance was assessed using one-way ANOVA with Tukey's post-test. ns: no significant difference, ****P < 0.0001.

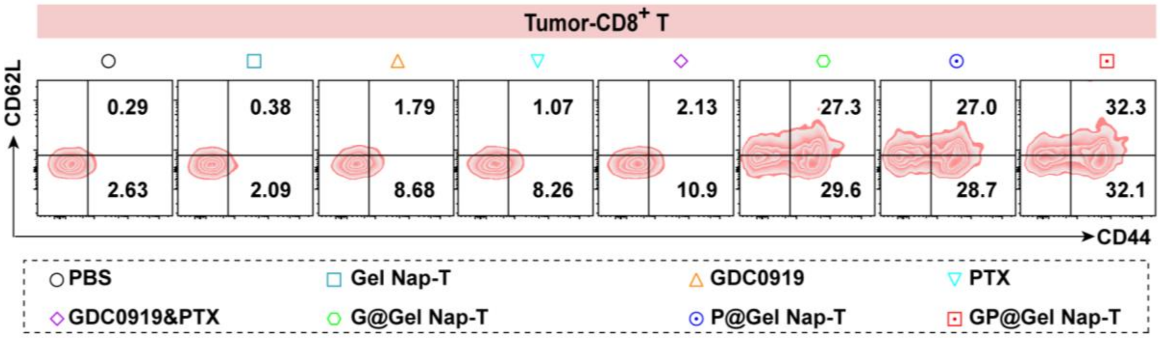

Figure S69. Representative FCM analysis of CD8$^+$ T$_{CM}$ (CD44$^+$CD62L$^+$) cells and CD8$^+$ T$_{EM}$ (CD44$^+$CD62L$^-$) cells in the tumor tissues from CMT167 tumor-bearing mice on day 14 after different treatments.

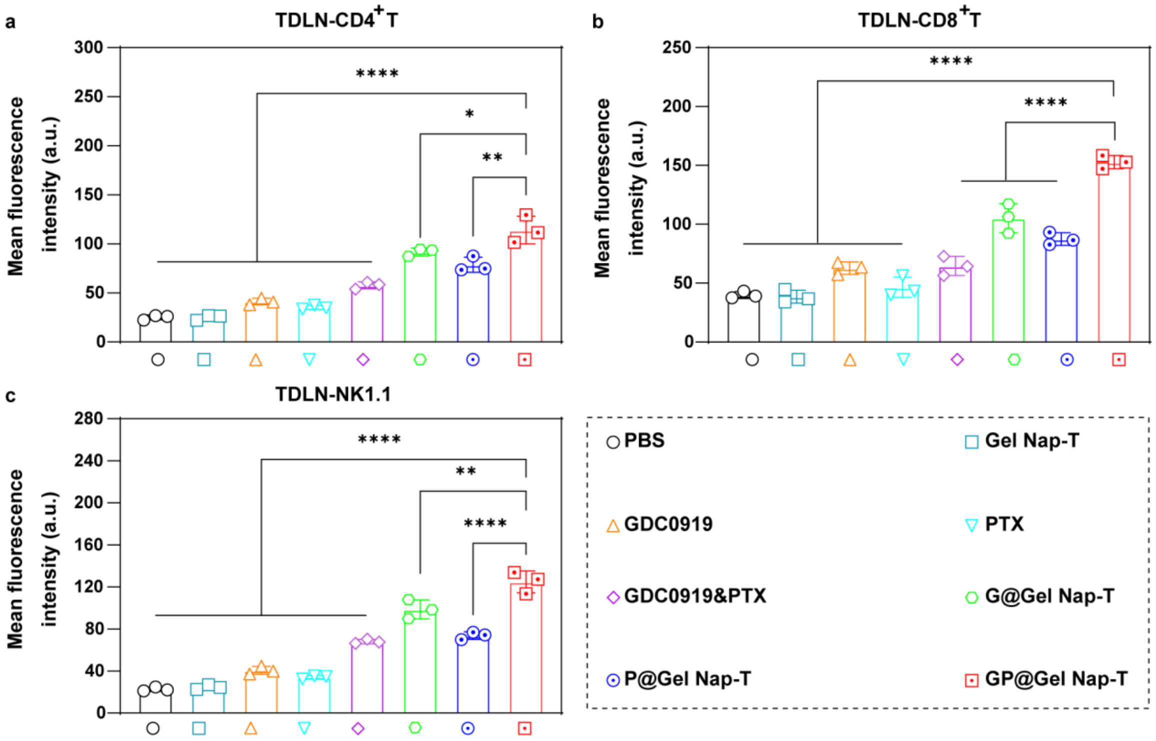

Figure S70. Corresponding quantitative data of (a) CD4$^+$ cells, (b) CD8$^+$ cells and (c) NK1.1$^+$ cells in Figure 7e (n = 3, biological independent samples). Results are presented as mean ± SD. Statistical significance was assessed using one-way ANOVA with Tukey's post-test. ns: no significant difference, *P < 0.05, **P < 0.01, ****P< 0.0001.

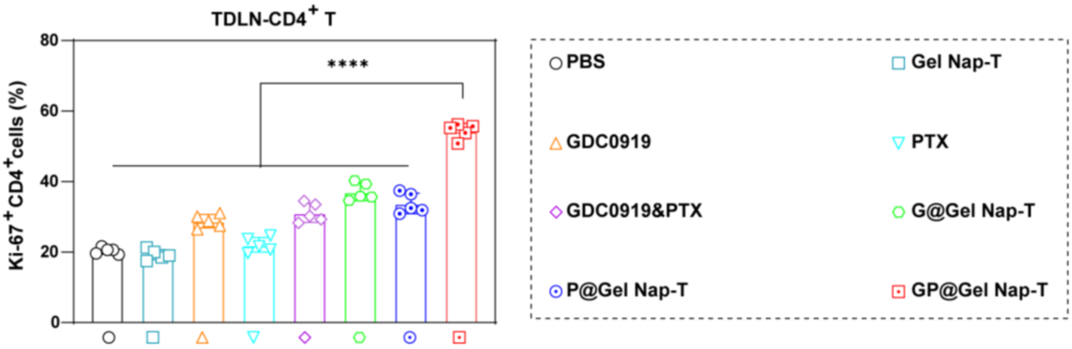

Figure S71. Quantitative analysis of the proportions of Ki-67^{+}CD4^{+} T cells in the TDLNs from CMT167 tumor-bearing mice on day 14 after different treatments (n = 5, biologically independent animals per group). Results are presented as mean ± SD. Statistical significance was assessed using one-way ANOVA with Tukey's post-test. ns: no significant difference, ****P < 0.0001.

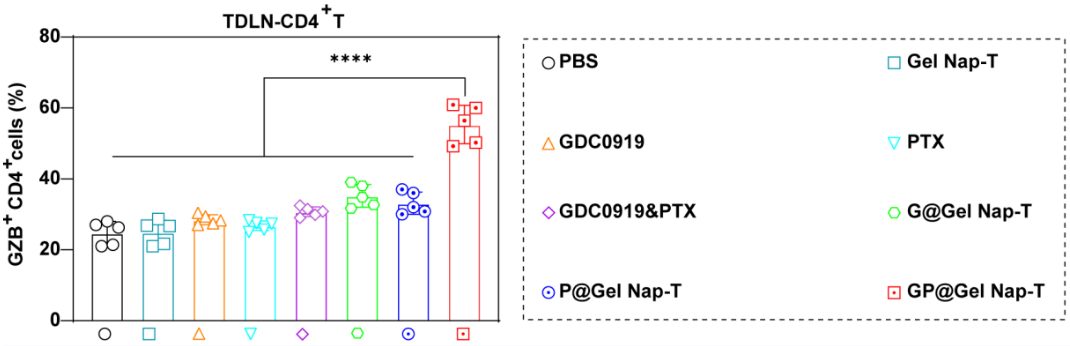

Figure S72. Quantitative analysis of the proportions of GZB^{+}CD4^{+} T cells in the TDLNs from CMT167 tumor-bearing mice on day 14 after different treatments (n = 5, biologically independent animals per group). Results are presented as mean ± SD. Statistical significance was assessed using one-way ANOVA with Tukey's post-test. ns: no significant difference, ****P < 0.0001.

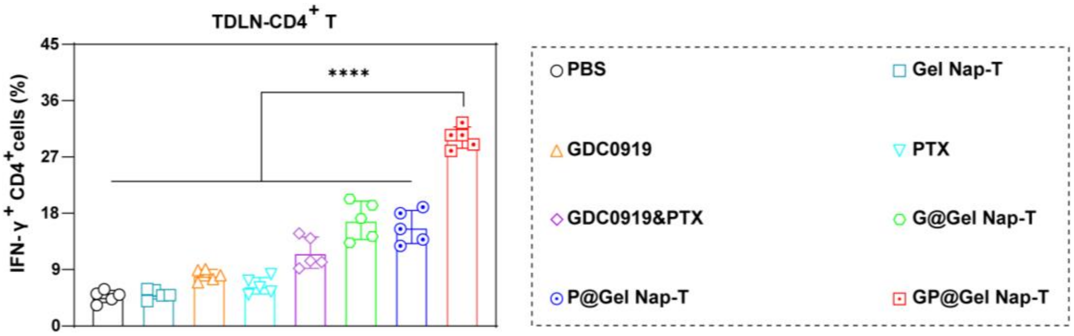

Figure S73. Quantitative analysis of the proportions of IFN-γ^{+}CD4^{+} T cells in the TDLNs from CMT167 tumor-bearing mice on day 14 after different treatments (n = 5, biologically independent animals per group). Results are presented as mean ± SD. Statistical significance was assessed using one-way ANOVA with Tukey's post-test. ns: no significant difference, ****P < 0.0001.

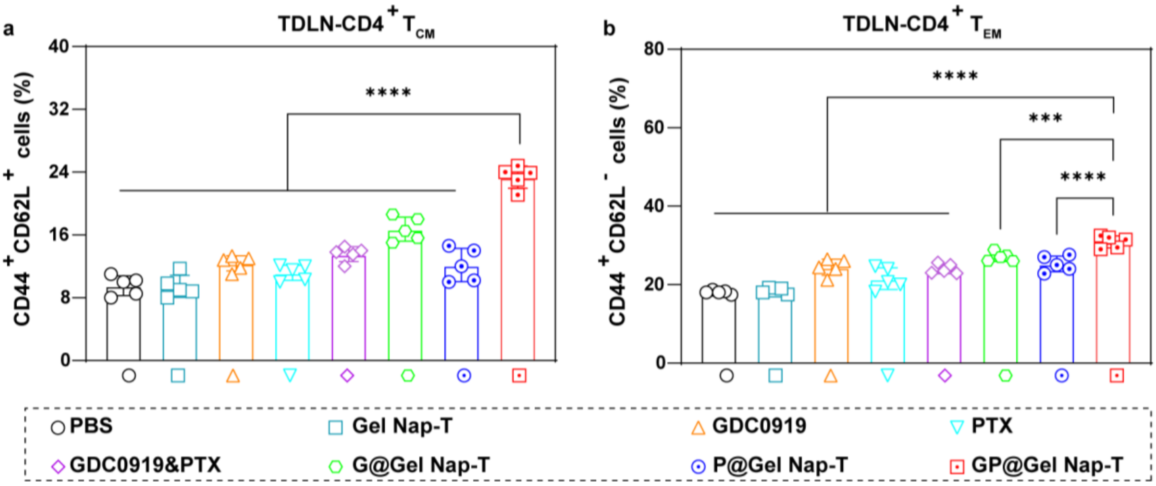

Figure S74. Quantitative analysis of the proportions of (a) $CD4^+$ T_{CM} ($CD44^+CD62L^+$) cells and (b) $CD4^+$ T_{EM} ($CD44^+CD62L^-$) cells in the TDLNs from CMT167 tumor-bearing mice on day 14 after different treatments (n = 5, biologically independent animals per group). Results are presented as mean ± SD. Statistical significance was assessed using one-way ANOVA with Tukey's post-test. ns: no significant difference, ***P < 0.001, ****P < 0.0001.

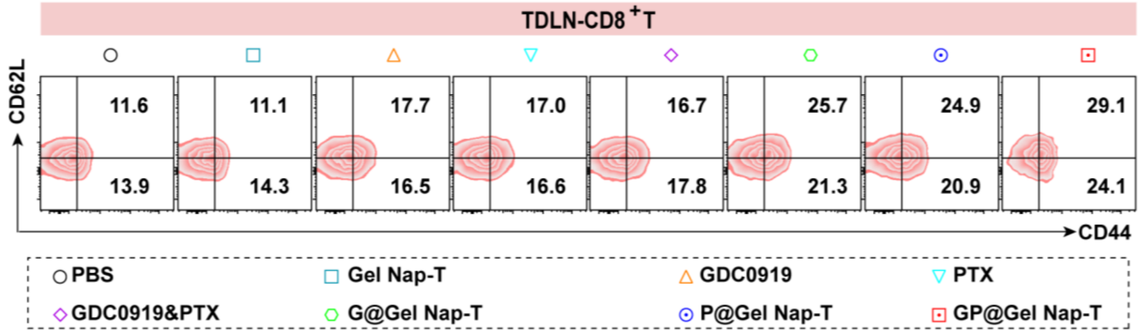

Figure S75. Representative FCM analysis of $CD8^+$ T_{CM} ($CD44^+CD62L^+$) cells and $CD8^+$ T_{EM} ($CD44^+CD62L^-$) cells in the TDLNs from CMT167 tumor-bearing mice on day 14 after different treatments.

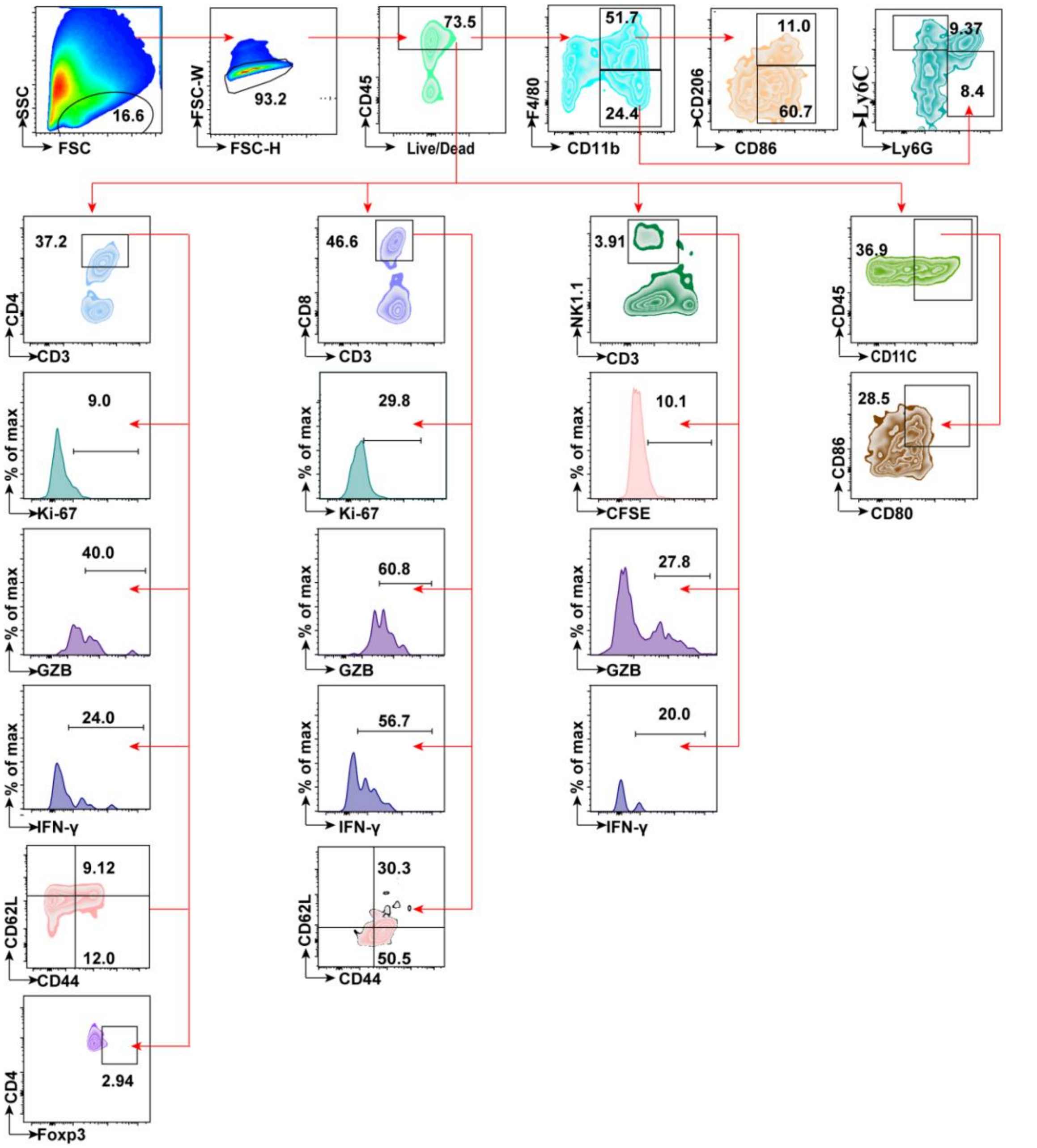

Figure S76. Gating scheme for FCM analysis for tumors and TDLNs.

3. Supporting Table

Table S1. Total drug loading capacities of **Gel Nap-T** hydrogels with different GDC0919/PTX/**Nap-T** molar ratios.

GDC0919: PTX: Nap-T (molar ratio)	Total drug loading content (wt %)
1: 1: 6.25	11.03
1: 1: 12.5	5.83
1: 1: 25	3.01
1: 1: 50	1.53
1: 1: 100	0.77
1: 1: 200	0.39
1: 1: 400	0.19

References

[1] B. Bacsa, C. O. Kappe, *Nat. Protoc.* **2007**, 2, 2222.
